# Supplementary material for: Developing outcome measures assessing wound management and patient experience: a mixed methods study
Source: BMJ Open. 2017 Nov 26;7(11):e016155. doi: 10.1136/bmjopen-2017-016155 (PMC5719294; doi:10.1136/bmjopen-2017-016155)
Supplement: Supplementary file 1 [file bmjopen-2017-016155supp001.pdf]

Table 1: Consolidated criteria for reporting qualitative research (COREQ)

	No	Item	Guide questions/description	Comment
**Domain 1: Research team and reflexivity**				
**Personal characteristics**	1	Interviewer/facilitator	Which author/s conducted the interview or focus group?	LR, DE, CMM and CM *(see pages 6 and 7)*
	2	Credentials	What were the researcher's credentials? e*.g. PhD, MD*	DE - BSc, PhD<br>LR - BSc, PhD<br>CMM - BA Hons, PgDip, MA PhD<br>CM – MB ChB BSc
	3	Occupation	What was their occupation at the time of the study?	DE - Qualitative Research Associate in Health Services Research<br>LR - Lecturer in Qualitative Health Science<br>CMM - Qualitative Research Fellow<br>CM - Research Fellow
	4	Gender	Was the researcher male or female?	Females
	5	Experience and training	What experience or training did the researcher have?	DE, LR and CMM have several years of experience conducting qualitative research. This has included completing many qualitative projects and attending training courses and workshops.
**Relationship with participants**	6	Relationship established	Was a relationship established prior to study commencement?	No
	7	Participant knowledge of the interviewer	What did the participants know about the researcher? *e.g. personal goals, reasons for doing the research*	The researchers introduced themselves, explained the purpose of the research and provided an information leaflet about the study
	8	Interviewer characteristics	What characteristics were reported about the interviewer/facilitator? *e.g. Bias, assumptions, reasons and interests in the research topic*	The researchers explained how qualitative research related to main Bluebelle trial
**Domain 2: study design**				
**Theoretical framework**	9	Methodological orientation<br>and theory	What methodological orientation was stated to underpin the study? *e.g. grounded theory, discourse analysis, ethnography, phenomenology, content analysis*	Data were analysed thematically using techniques of constant comparison derived from grounded theory methodology *(see page 6)*

**Participant selection**	10	Sampling	How were participants selected? *e.g. purposive, convenience, consecutive, snowball*	Purposeful *(see pages 6 and 7)*
	11	Method of approach	How were participants approached? e.*g. face-to-face, telephone, mail, email*	Patients were approached face to face by healthcare professionals. Healthcare professionals were contacted by the researchers via email. *(See pages 6 and 7)*
	12	Sample size	How many participants were in the study?	Sixty-four patients and 15 health care professionals from abdominal general surgical specialities and obstetrics *(See Table 1 on pages 7/8)*
	13	Non-participation	How many people refused to participate or dropped out? Reasons?	Two patients were unable to take part due to poor health.
**Setting**	14	Setting of data collection	Where was the data collected? *e.g. home, clinic, workplace*	Patient interviews were conducted whilst patients were in hospital. Health professionals chose a location that was convenient for them (their workplace or a nearby café) or opted to do the interview over the telephone. *(See pages 6 and 7)*
	15	Presence of non-participants	Was anyone else present besides the participants and researchers?	The partners of patients sometimes sat with the patients but spoke very little; their comments were not included in the final analysis.
	16	Description of sample	What are the important characteristics of the sample? *e.g. demographic data, date*	Participants' full details are provided in Table 1, and key information is provided in the results section *(See pages 7/8)*
**Data collection**	17	Interview guide	Were questions, prompts, guides provided by the authors? Was it pilot tested?	A topic guide was developed (based on literature and views of health care professionals in the Bluebelle study team) to ensure that discussions covered the same core issues but with sufficient flexibility to allow new issues of importance to the informants to emerge. Although not piloted, it was adapted as analysis progressed to enable exploration of emerging themes. *(Topic guides are included in Additional File)*
	18	Repeat interviews	Were repeat interviews carried out? *If yes, how many?*	No repeat interviews were carried out
	19	Audio/visual recording	Did the research use audio or visual recording to collect the data?	Interviews were audio-recorded *(see page 6)*
	20	Field notes	Were field notes made during and/or after the	The researchers kept notes throughout data

			interview or focus group?	collection and analysis *(See page 8)*
	21	Duration	What was the duration of the interviews or focus group?	Interviews lasted an average of 25 minutes (range = 15–50 minutes). *(See page 8)*
	22	Data saturation	Was data saturation discussed?	Data collection continued until the team were confident that saturation had been reached *(See page 7)*
	23	Transcripts returned	Were transcripts returned to participants for comment and/or correction?	Transcripts were not returned to participants for comments or corrections
**Domain 3: analysis and findings**				
**Data analysis**	24	Number of data coders	How many data coders coded the data?	All data were coded by DE or CMM. A subset of approximately half of the interviews (n = 19) was double coded by a third researcher (LR). *(See page 7)*
	25	Description of the coding tree	Did authors provide a description of the coding tree?	A list of issues from the analysis of the interviews and literature search was collated into an item tracking matrix. *(See additional File)*
	26	Derivation of themes	Were themes identified in advance or derived from the data?	Issues which were conceptually similar were organised into categories. For instance, issues such as ‘itchiness/irritation’, ‘presence of pulling sensation’, and ‘tightness of wound’ were mapped into a ‘wound comfort’ category. *(See Table 2)*
	27	Software	What software, if applicable, was used to manage the data?	NVivo (version 10) was used to analyse the data (See page 6)
	28	Participant checking	Did participants provide feedback on the findings?	Full results were not sent out to all participants to gain respondent validation.
**Reporting**	29	Quotations presented	Were participant quotations presented to illustrate the themes / findings? *Was each quotation identified? e.g. participant number*	The interpretation of each category is supported by illustrative quotes *(See Table 2)*
	30	Data and findings consistent	Was there consistency between the data presented and the findings?	There is consistency between the data presented and the measures developed. The item tracking matrix provides an overview of the key findings and how these were used to develop the initial measure *(See additional file)*
	31	Clarity of major themes	Were major themes clearly presented in the findings?	The themes are clearly presented in the

				findings *(See pages 8-11)*
	32	Clarity of minor themes	Is there a description of diverse cases or discussion of minor themes?	Yes. Differences between the findings of the interviews and the literature search are discussed, as are the differences in satisfaction between the dressing types *(See pages 9 and 10)*
