# Supplementary material for: Developing outcome measures assessing wound management and patient experience: a mixed methods study
Source: BMJ Open. 2017 Nov 26;7(11):e016155. doi: 10.1136/bmjopen-2017-016155 (PMC5719294; doi:10.1136/bmjopen-2017-016155)
Supplement: Supplementary file 2 [file bmjopen-2017-016155supp002.pdf]

**Interview Topic Guide: Phase 1: Generation of relevant issues**

**<u>Background, interviewee details and ice breaker</u>**

- Interviewee background and details of procedure that the interviewee has had (type of procedure elective or emergency? have you had before?, when, where, length of stay in hospital, recovery period).
  - *Just to give me some background, can you just tell me a little bit about the procedure that you had, the surgical procedure?*
  - *How have you been, in terms of recovery? Length of stay? When came home?*
  - *Is this your first?*

**<u>Expectations/experiences of wound care</u>**

- *Did/do you have any expectations about whether or not you would have a dressing? Where do you think these expectations came from?*
- *What kind of dressing? When did they take it off? [home/hospital?] Same one? How did you find having the dressing? Why do you think you had/did not have a dressing? Do you think it had any impact on your wound healing?*
- *Why do you think they do/don't use dressings?*
- *Thoughts/reactions to **alternative** wound management methods (i.e. dressing or no dressing in relation to what patient has experienced/what patient expects). (Explore patient thoughts on impact this may have on previously mentioned issues (e.g. recovery, symptoms, practicalities).*

**<u>Patient perspectives on a trial of dressing type</u>**

Okay, the basis of this study is like I said, we're trying to find out if dressing the wounds is helpful or not. In a study, patients would be randomly allocated by chance to either receive dressings or receive no dressings. The doctor wouldn't decide and the patient wouldn't decide. What we're trying to find out is, in patients who have had a similar type of surgery, if we have one group of patients that have them on so and another group of patients that don't, then can we compare them to see if there's any difference between the groups.

- *Do you think you would participate? (Reasons why/why not? Any reservations?)*
- *Can you imagine your family and/or friends would participate in a study like this?*
- *What kinds of things do you think you might consider, when deciding whether or not to take part? Do you think you would have any questions about the study? What kind of things might you want to know about in advance?*
- *How do you think you would feel about random allocation to dressing type, specifically the possibility of receiving no wound dressing? [explain randomisation to patients, then ask…] If you were in a group that didn't receive a dressing, do you think you would have thought about your care differently?*
- *In paediatrics, they don't use dressings. Changes impression?*
- *What about a glue dressing?*
- *Can you think of any potential problems we might come across if we were doing this study, any practical problems or any difficulties we might come across?*
- *Perspectives on important outcomes to include in a trial of dressing use. We would like this study to help us answer the question of whether dressings should be used in patients such as yourself (and if so, what type of dressing is best). What do you think are the important factors we should consider when making any future recommendations on dressing use? Satisfaction? Infection? Healing times?*

**Closing**

- Summarise key points
- Any further questions?
- Thank patient for their time and explain how they have helped.

**Interview Topic Guide: Phase 3 – Pre-testing**

**Introduction**

- As you know, we are going to audio-record you whilst you complete the questionnaire that we have developed. During this, we would like to understand your opinion of the questions, in terms of the language used and the relevance to your post-operative experience. It is important to remember that there are no right or wrong answers, but that this will help us to understand how the questionnaire should be developed. This should take no longer than an hour, although you can stop the discussion at any time. The information that you provide is not going to be fed back to your clinical team.
- Complete consent form and data collection form
- Any questions?

**Background (procedure and recovery)**

- Could you tell me a little bit about what you had done? *(Probe: When? Where? Elective/emergency?)*
- How has recovery been for you so far? *(Probe: How feeling? Length of stay? When expected to go home?)*
- How has the wound been healing? *(Probe: How does wound feel? Any problems?)*

**PWMQ: Can we work through the questionnaire as you complete it, with you explaining how you understand the items and what you will put for this question.**

- DT to not clarify meaning, but just repeat question.
- If P uncertain, try to understand why.
- Note any potential issues to be discussed after completion.

**PWMQ: Understanding responses to each question**

- Can you tell me in your own words what that question was asking? *(Probe: What does the word XX mean to you? Are there any other ways you would describe it?)*
- What does [not at all/a little/quite a bit/very much] mean to you?
- Was it easy to choose an answer?
- Explore where participants have indicated confusion

**After completion:**

- What did you think of the questionnaire?
- Were the questions relevant to your experience? *(Probe: Are there any others that should be included?)*
- Any difficulties filling in questions?
- Do you think it captures your experience of your wound in the past 24 hours?
- What does the word "practical wound management" mean to you?
- Do you have any other suggestions on how the questionnaire could be improved? *(Probe: alternative wording, additional questions, response categories, layout/presentation, length of questionnaire?)*
- You have given your answers based on the past 24 hours. Would your answers differ if you were thinking about your entire recovery from surgery? *(Probe: When do you think would be the best time to complete questionnaires like these?)*

**Closing**

- Summarise key points
- Any further questions?
- Thank patient for their time and explain how they have helped.
