# Supplementary material for: Developing outcome measures assessing wound management and patient experience: a mixed methods study
Source: BMJ Open. 2017 Nov 26;7(11):e016155. doi: 10.1136/bmjopen-2017-016155 (PMC5719294; doi:10.1136/bmjopen-2017-016155)
Supplement: Supplementary file 3 [file bmjopen-2017-016155supp003.pdf]

Table 1: Item tracking matrix of all issues identified									
**Category**	**Issue**	**Identified by**				**Additional comments**	**Included in questionnaire?**	
		**Interviews**			**Literature (n=26 RCTs)**		**If yes, questionnaire item**	**Why not included**
		**DE (n=28)**	**LR (n=19)**	**CM (n=11)**				
**Wound comfort**	Itchiness	✓	✓	✓	✓	CM: Described by patients as an outcome (looking at the wound to check if it is irritated, inflamed, etc)<br>DE: Similar to inflammation ('burning'	Q1: Has the wound been itchy?	
	Pain	✓	✓	✓	✓	DE: Described as 'sore', 'hurt', 'tender', 'uncomfortable'.<br>DE: Sometimes mentioned in the context of a numerical scale.<br>RM: Described as 'burning pain referring to a dressing-related sensation felt under the dressing' in one study<br>RM: Also 'tenderness'<br>CM: 'sore', 'painful' – discussed in terms of dressing removal	Q2: Has the wound been painful?	
	Presence of pulling sensation	✓			✓	LR: Described as wound 'being able to breathe', 'stuffiness'	Q3: Has the wound had a pulling sensation?	
	Tightness of wound	✓	✓		✓		Q4: Has the wound felt tight?	
	Wound comfort (overall or unspecified)				✓	RM: Also measured as 'Discomfort' in the literature<br>RM: Includes 'Discomfort with skin problems'		Excluded as covered in Q1 – Q4
**Exudate and its impact**	Whether there was any exudate	✓	✓	✓	✓		Q5: Has the wound leaked?	
	Type of exudate (blood, other)	✓	✓	✓	✓	DE: Described as 'mess', 'manky', 'leaking', 'gunge', 'oozing', 'soaking', 'brown mess'<br>LR: 'moistness', Ooziness', 'dampness'<br>RM: Described as 'discharge' 'fluid' 'oozing'<br>CM: 'Seeping'	Q5: If so, was it: clear fluid? cloudy fluid? Blood-stained fluid? thick and yellow/green fluid?	
	Whether exudate marks bedding/clothing	✓	✓	✓		DE: Described as 'stains', LR: 'manked up clothing'	Q6: Has the leakage resulted in changed bedding/ clothes?	
	Degree dressing absorbs exudate	✓	✓	✓	✓		Q7: How would you describe the wettest dressing?	
	Whether additional dressing required	✓	✓	✓	✓	RM: Includes reasons for dressing changes<br>CM: use two dressings when oozing is important, don't want to take original dressing off	Q8: Has a dressing or glue been put on the wound (or replaced)?	
	Anxiety associated with exudate	✓	✓	✓				Excluded as captured in Q15: "Have you felt any anxiety about your wound?"
**Allergic reactions to the dressing**	Any allergic reactions to dressing/blistering	✓	✓	✓	✓	RM: Also include skin damage/injury	b) has the wound blistered?	
**Dressing removal**	Whether dressing comes off	✓	✓	✓			Q9: Has the dressing or glue come off or been removed	
	Whether dressing needs to be taken off	✓	✓	✓		CM: patient or partner	Q9 Q9: Has the dressing or glue come off or been removed?	

	(if so, by patient or professional)						If "Yes", was it taken off by a doctor/nurse/other health specialist?	
	Whether travel is required to change/remove dressing (i.e. seeing nurse or GP/post op visits)	✓	✓					Not relevant to early post-operative period
	Any discomfort during removal	✓	✓	✓	✓		Q10 Was there any discomfort when removing the dressing?	
	Any pain during removal	✓	✓	✓		DE: Causes 'pain', skin is 'tender', 'sore', pulls hairs, sticks to skin)<br>RM: 'Pain on removal of the dressing'	Q11 Was there any pain when removing the dressing?	
**Wound protection**	Dressings protecting the wound				✓		Q12 Has the wound felt protected? (i.e. from catching on anything or being knocked)	
	Whether dressing/wound rubs on clothes	✓	✓	✓		LR: Awkwardness of wearing clothes over dressing		
	Whether dressing/wound catches on other things	✓	✓	✓		CM: bedsheets		
**Impact on daily activities**	Ability to get back to work	✓	✓				Q13: Have you been able to perform everyday tasks? (i.e. showering/ bathing, getting dressed)	
	Ability to shower/bathe	✓	✓	✓	✓	RM: Described as 'Ability to facilitate personal hygiene' in one study<br>RM: Described as 'Appreciation of possibility to shower' in one study. Also 'satisfaction with the possibility to wash oneself'		
	Ease of movement (e.g sitting, walking, stairs)	✓	✓		✓	DE: Standing up, walking<br>RM: Described as 'Ability to facilitate mobility' in the literature. Also 'Does the dressing limit you in movement?'<br>DE and LR: Includes sneezing/coughing		
	Ability to perform everyday tasks (e.g self-care)	✓	✓	✓		DE: Washing/self-care, driving, walking, housework, cooking, exercise		
	Ease of getting dressed	✓						
	Going to the toilet		✓	✓				
	Self-management of wound		✓	✓	✓	RM: 'Ease of managing wound' was a PRO measured in the first 3 weeks after surgery on a 1-10 scale in one study		
	Change to usual clothing		✓	✓				
	Overall recovery				✓	RM: not a PRO (surgeon rating)		
**Ease of movement**	(Dis)comfort when sitting	✓	✓	✓			Q14: Have you been able to move around easily?	
	(Dis)comfort when lying	✓	✓					

	(Dis)comfort whilst sleeping/sleep quality	✓	✓					
	(Dis)comfort whilst moving	✓	✓	✓		CM: related to tightness of dressing		
**Anxiety about the wound**	Feeling of security/safeness (in relation to the wound?)	✓	✓	✓			Q15: Have you felt any anxiety about your wound?	
	Feeling of vulnerability		✓	✓				
	Not having to worry about wound/dressing	✓	✓	✓		DE: ‘You can just forget about it, you don’t have to think about		
	Feeling protected	✓	✓	✓				
	Stress levels/psychological discomfort/anxiety	✓	✓					
	Feeling constricted by dressing	✓	✓					
	Cleanliness of environment	✓	✓	✓		DE: Described as ‘hygiene’, ‘coming into contact with bugs’		
	Fear of infection	✓	✓	✓				
	Anxiety about bodily contents spilling out/wound bursting open	✓	✓	✓		LR: Described as ‘coming open’, ‘split apart’, ‘rupture’		
**Satisfaction with dressing/ not having a dressing**	Satisfaction with (appearance of) dressing	✓	✓		✓	LR: ‘Neatness’, ‘prominence’<br>RM: Measured as ‘How satisfied overall do you feel with your dressing?’	Q16: Have you felt satisfied with having/not having a dressing?	
	Whether patient would prefer to see the wound	✓	✓			DE: Anxiety/reassurance/fear/discomfort<br>RM: Described as ‘how well the incision could be seen under the dressing’ and ‘transparency’		
	Degree dressing fits contours of the skin/clothing	✓	✓		✓	RM: Described as ‘Conformability of the dressing to the wound’ in one study		
	Dignity			✓		CM: Having a dressing gives more dignity		
	Confidence			✓		CM: Confidence to walk around without having to worry		
	Appreciation of absence of bandage				✓			
	How long dressing stays on	✓	✓	✓	✓	CM: ‘the longer you leave a dressing on the harder it is to get off’		
	Ease of removal	✓	✓	✓	✓	RM: ‘Ease of dressing application’ was measured in one study but this was rated by a surgeon not patient<br>LR: Any exudate from removal<br>DE, CM,: Any remnants remaining after removal (‘bits of glue’)		
	Degree of stickiness	✓	✓	✓	✓	CM: stitches stick to legs when sitting (gynae)		

						DE: Mostly considered to be positive, but sometimes makes removal tricky<br>DE: Described as: ‘It stays in place and doesn’t wriggle up’<br>RM: Described as ‘Dressing integrity’ in one study also ‘How much the dressing had loosened’<br>CM: dressings don’t stick well because of body hair		
	Awareness of dressing/wound	✓	✓	✓	✓			
	If patient reapplies, ease of reapplying dressing	✓	✓		✓			
	Whether additional support or materials provided	✓	✓	✓				
	Whether patient uses own dressing	✓	✓	✓				
	If patient reapplies, ease of reapplying dressing	✓	✓		✓			
	Overall satisfaction/satisfaction with overall experience				✓			
**Wound appearance**	Perceptions of healing	✓	✓	✓	✓	DE: Described as ‘healing very nicely’, ‘rate of healing’<br>LR: Sub-codes – ‘quality of healing’, ‘speed of healing’, ‘whether healed or not’, ‘suggested as main outcome’. Patients often discuss scab formation when talking about healing.<br>RM: Measured as ‘Effectiveness (wound healing)’ in one study 1= well healed, 3=poorly healed, not a PRO. Also measured as a PRO in another study ‘Has your wound healed?’<br>CM: Healing could be a standalone category (one of the outcomes): definition of healing, healing time		Relevant to longer term outcomes of wound healing (not relevant within first days of surgery)<br>Patients’ wounds not visible if dressing applied
	Bruising	✓	✓			DE: Described as ‘black and blue’		
	Colour	✓	✓	✓	✓	DE: Described as ‘purple’/’pink’/’grey’/’red’ (see inflammation’)<br>LR: ‘Black and bluish’ (also fit under ‘bruising’, as above)<br>RM: Redness<br>CM: Colour was talked about more in the context of healing, ie as an indicator (for the patients) of whether or not the wound was healing		
	Cosmesis/aesthetics		✓		✓	RM: Described as ‘cosmesis’, ‘cosmetic outcome’ and ‘cosmetic result’ in the literature		

	Scars	✓	✓	✓	✓	DE: A long term measure<br>RM: Pigmentation, scar colour, prescence of inflammation, suppleness or pliability, scar height or evenness with the surrounding skin, using modified Vancouver Burn Assessment Scale		
	Size	✓	✓	✓				
	Scabbing	✓	✓	✓		DE: Associated with healing		
	Inflammation/swellin g	✓	✓	✓		DE: Described as ‘burning’<br>CM: Described by patients as an outcome		
	Overall appearance of wound	✓	✓	✓		DE: Described as ‘unsightly’, ‘neat’, ‘tidy’, ‘messy’ LR: ‘ugly’<br>CM: ‘Smooth’		
	Maceration of the skin				✓	RM: not a PRO		
	Satisfaction with the appearance of the wound				✓			
	Whether patient would prefer to see the wound	✓	✓					
