# Supplementary material for: Developing outcome measures assessing wound management and patient experience: a mixed methods study
Source: BMJ Open. 2017 Nov 26;7(11):e016155. doi: 10.1136/bmjopen-2017-016155 (PMC5719294; doi:10.1136/bmjopen-2017-016155)
Supplement: Supplementary file 4 [file bmjopen-2017-016155supp004.pdf]

Outcome as described by author	Wording used to measure outcome (where reported)	Who reported the outcome	Rating/measurement scale	Assessment time point	Study reference	Study source *
Pain	-	Patient reported	0 to 10 cm VAS	Day 1, Day 10	Amin 2009	2,3
Cosmesis of wound	Cosmesis of wound	Patient reported	Poor 0 5 Excellent 10	3 months post-op	Amin 2009	2,3
Ability to shower same day	Ability to shower same day	Patient reported	Poor (0) Satisfactory (1) Excellent(2)	3 months post-op	Amin 2009	2,3
Need visit GP for wound care	Need visit GP for wound care	Patient reported	"Could have done without; Didn't mind; N/A"	3 months post-op	Amin 2009	2,3
Pain on removing clips	Pain on removing clips	Patient reported	Yes/No	3 months post-op	Amin 2009	2,3
Pain/Tightness of wound after 3 months	Pain/Tightness of wound after 3 months	Patient reported	Slight(0)Moderate(1)Significant(2)	3 months post-op	Amin 2009	2,3
Overall comfort with wound	Overall comfort with wound	Patient reported	Poor(0)Satisfactory(1)Excellent(2)	3 months post-op	Amin 2009	2,3
Allergic reactions	Allergic reactions	Patient reported	Yes(0)No(1)	3 months post-op	Amin 2009	2,3
Overall satisfaction	Overall satisfaction	Patient reported	Poor 0; 5; 10 Excellent	3 months post-op	Amin 2009	2,3
Cosmetic appearance	How would you evaluate your skin stitches after the operation?	Patient reported	1 (very poor) 2 (poor) 3 (medium) 4 (good) 5 (very good)	Day 40 post-op	Asvar 2009	2
Satisfaction	What is your satisfaction?	Patient reported	1 (very poor) 2 (poor) 3 (medium) 4 (good) 5 (very good)	Day 40 post-op	Asvar 2009	2
Perceived patient satisfaction	-	Physician reported	"Ratings"	Day 10	Blondeel 2004	2,3
Cosmesis	-	Patient reported	"Ratings" – unspecified categories but included "outstanding"	Day 10	Blondeel 2004	2,3
Overall comfort	-	Patient reported	"Ratings"	Day 10	Blondeel 2004	2,3

* 1= Cochrane 2011 dressings review; 2= Cochrane 2014 tissue adhesive review; 3=Chow 2010 tissue adhesive review; 4=additional studies provided by authors of the Cochrane dressings review update

Outcome as described by author	Wording used to measure outcome (where reported)	Who reported the outcome	Rating/measurement scale	Assessment time point	Study reference	Study source *
Ability to shower	-	Patient reported	"Ratings"	Day 10	Blondeel 2004	2,3
Dressing changes	-	Patient reported	"Ratings"	Day 10	Blondeel 2004	2,3
Tension at the wound	-	Patient reported	"Ratings"	Day 10	Blondeel 2004	2,3
Hygiene problems	-	Patient reported	"Ratings"	Day 10	Blondeel 2004	2,3
Allergic reaction	-	Patient reported	"Ratings"	Day 10	Blondeel 2004	2,3
Overall satisfaction	-	Patient reported	"Ratings"	Day 10	Blondeel 2004	2,3
Cosmesis	-	Physician reported	validated Modified Hollander Instrument	Day 30	Blondeel 2004	2,3
Dressing changes	-	Observer reported	n/a	unspecified	Burke 2012	4
Incidence of blistering	-	Observer reported	n/a	unspecified	Burke 2012	4
Cosmetic outcome	-	Unspecified	Scale, 1-10	Unclear when specific outcomes were recorded – several follow ups at Day 1, 3, 5-7 and 2 weeks, 1, 3, 6, 12 months	Chibbaro 2009	2,3
Wound management by the patients	-	Unspecified	Unspecified	Unclear when specific outcomes were recorded – several follow ups at Day 1, 3, 5-7 and 2 weeks, 1, 3, 6, 12 months	Chibbaro 2009	2,3
Satisfaction	-	Patient reported	Scale, 1-10	Unclear when specific outcomes were recorded – several follow ups at Day 1, 3,	Chibbaro 2009	2,3

* 1= Cochrane 2011 dressings review; 2= Cochrane 2014 tissue adhesive review; 3=Chow 2010 tissue adhesive review; 4=additional studies provided by authors of the Cochrane dressings review update

Outcome as described by author	Wording used to measure outcome (where reported)	Who reported the outcome	Rating/measurement scale	Assessment time point	Study reference	Study source *
				5-7 and 2 weeks, 1, 3, 6, 12 months		
Appreciation of possibility to shower	-	Unspecified	Unspecified	Unclear when specific outcomes were recorded – several follow ups at Day 1, 3, 5-7 and 2 weeks, 1, 3, 6, 12 months	Chibbaro 2009	2,3
Appreciation of absence of head bandage	-	Unspecified	Unspecified	Unclear when specific outcomes were recorded – several follow ups at Day 1, 3, 5-7 and 2 weeks, 1, 3, 6, 12 months	Chibbaro 2009	2,3
Number of dressing changes	-	Collected by surgeons, nurses and junior medical staff	n/a	during hospital stay	Cosker 2005	1
Blistering	-	Collected by surgeons, nurses and junior medical staff	n/a	during hospital stay	Cosker 2005	1
When the dressing required changing	-	Collected by surgeons, nurses and junior medical staff	n/a	during hospital stay	Cosker 2005	1
Reason for dressing change	-	Collected by surgeons, nurses and junior medical staff	n/a	during hospital stay	Cosker 2005	1
Number of post operative days	-	Collected by surgeons, nurses and junior medical staff	n/a	during hospital stay	Cosker 2005	1

* 1= Cochrane 2011 dressings review; 2= Cochrane 2014 tissue adhesive review; 3=Chow 2010 tissue adhesive review; 4=additional studies provided by authors of the Cochrane dressings review update

Outcome as described by author	Wording used to measure outcome (where reported)	Who reported the outcome	Rating/measurement scale	Assessment time point	Study reference	Study source *
Volume of wound exudate	-	Collected by surgeons, nurses and junior medical staff	Amount of seepage through the dressing and the apparent volume found on the wound after the dressing was removed	during hospital stay	Cosker 2005	1
Satisfaction with wound closure method	-	Patient reported	Satisfied / Dissatisfied	24 to 48 hrs, 4 to 6 weeks, 3 months post-op	Dowson 2006	2,3
Satisfaction with appearance of the wound	-	Patient reported	Satisfied / Dissatisfied	24 to 48 hrs, 4 to 6 weeks, 3 months post-op	Dowson 2006	2,3
Degree of pain	-	Patient reported	Scale, 1-10	First 3 weeks after surgery	Gennari 2004	3
Ease of managing wound	-	Patient reported	Scale, 1-10	First 3 weeks after surgery	Gennari 2004	3
Ability to take a shower	-	Patient reported	Scale, 1-10	First 3 weeks after surgery	Gennari 2004	3
Postoperative visits	-	Patient reported	Scale, 1-10	First 3 weeks after surgery	Gennari 2004	3
Use of dressings	-	Patient reported	Scale, 1-10	First 3 weeks after surgery	Gennari 2004	3
Comfort	-	Interview with physician	Unspecified	1, 2, & 4 weeks post-op	Greene 1999	2,3
Presence of a pulling sensation	-	Interview with physician	Unspecified	1, 2, & 4 weeks post-op	Greene 1999	2,3
Appreciation of the lack of suture removal	-	Interview with physician	Unspecified	1, 2, & 4 weeks post-op	Greene 1999	2,3
Discomfort in connection with removal of dressing	-	Unspecified	Unspecified	during hospital stay	Holm 1998	1
Number of dressing changes	-	Observer reported	n/a	during hospital stay	Holm 1998	1

* 1= Cochrane 2011 dressings review; 2= Cochrane 2014 tissue adhesive review; 3=Chow 2010 tissue adhesive review; 4=additional studies provided by authors of the Cochrane dressings review update

Outcome as described by author	Wording used to measure outcome (where reported)	Who reported the outcome	Rating/measurement scale	Assessment time point	Study reference	Study source *
Adhesion of dressing to the skin	-	Observer reported	Unspecified	daily inspection until discharge	Holm 1998	1
Cosmetic result	-	Observer reported	1 to 5 (higher=better)	3 months post-op	Holm 1998	1
Width of the scar	-	Observer reported	1 to 5 (higher=better)	3 months post-op	Holm 1998	1
Downbinding of the scar	-	Observer reported	1 to 5 (higher=better)	3 months post-op	Holm 1998	1
Colour of the scar	-	Observer reported	1 to 5 (higher=better)	3 months post-op	Holm 1998	1
Elevation of the scar	-	Observer reported	1 to 5 (higher=better)	3 months post-op	Holm 1998	1
Cosmetic outcome	-	Observer reported	1 to 5 (higher=better)	3 months post-op	Holm 1998	1
Supposed inconvenience of the scar	-	Observer reported	1 to 5 (higher=better)	3 months post-op	Holm 1998	1
Exudate	-	Observer reported	Unspecified	daily inspection until discharge	Holm 1998	1
Leakage	-	Observer reported	Unspecified	daily inspection until discharge	Holm 1998	1
Transparency	-	Observer reported	Unspecified, but included milky and slightly milky	daily inspection until discharge	Holm 1998	1
Dressing changes	-	Observer reported	No. of days stayed in place	unspecified	Holm 1998	1
Reasons for dressing changes	-	Observer reported	Unspecified	unspecified	Holm 1998	1
Maceration of the skin	-	Observer reported	Unspecified	unspecified	Holm 1998	1
Post operative wound infection	-	Observer reported	Unspecified	unspecified	Holm 1998	1
Comfort	-	Observer reported	Yes/No	1 week and 1 month	Keng 1989	2
Cosmesis	-	Observer reported	1 (poor) to 5 (excellent)	1 week and 1 month	Keng 1989	2

* 1= Cochrane 2011 dressings review; 2= Cochrane 2014 tissue adhesive review; 3=Chow 2010 tissue adhesive review; 4=additional studies provided by authors of the Cochrane dressings review update

Outcome as described by author	Wording used to measure outcome (where reported)	Who reported the outcome	Rating/measurement scale	Assessment time point	Study reference	Study source *
Satisfaction with the incision closure	-	Patient verbal report; response recorded by staff	Either “satisfied” or “dissatisfied”	3 month follow up	Kent 2014	2
Overall appearance	-	Patient verbal report; response recorded by staff	Either “satisfied” or “dissatisfied”	3 month follow up	Kent 2014	2
Satisfaction with the techniques of skin closure	-	Patient reported	VAS between 0 and 100, where 100 represented maximal satisfaction.	Between 8 and 12 weeks post-op	Khan 2006	2,3
Dressing or superficial wound discomfort	-	Unspecified	Linear analogue scale	5 days post -op	Law 1987	1
Dressing preference	-	Unspecified	Unspecified	unspecified	Law 1987	1
Wound infection	-	Unspecified	Discharge of purulent material	unspecified	Law 1987	1
Number of dressing changes	-	Unspecified	n/a	unspecified	Law 1987	1
Quality of the final scar	-	Unspecified	Unspecified	unspecified	Law 1987	1
Blisters	-	Observer reported	Defined as any lifting of the epidermis with underlying fluid	48 hours post-op and 5 days	Lawrentschu k 2002	1
Wound condition	-	Observer reported	Unspecified	5 days	Lawrentschu k 2002	1
Swelling	-	Observer reported	Measured thigh girth	48 hours post-op and 5 days	Lawrentschu k 2002	1
Wound infection	-	Observer reported	Unspecified	unspecified	Lawrentschu k 2002	1
Cosmetic appearance	-	Patient reported	100mm VAS (0 worst outcome, 100 best outcome)	3 months	Livesey 2009	2,3
Satisfaction with the scar	-	Patient reported	100mm VAS (0 extreme dissatisfaction, 100 complete satisfaction)	3 months	Livesey 2009	2,3

* 1= Cochrane 2011 dressings review; 2= Cochrane 2014 tissue adhesive review; 3=Chow 2010 tissue adhesive review; 4=additional studies provided by authors of the Cochrane dressings review update

Outcome as described by author	Wording used to measure outcome (where reported)	Who reported the outcome	Rating/measurement scale	Assessment time point	Study reference	Study source *
Appearance of the wound	-	Patient reported	5 point Likert scale (1=much better than expected, 2= better than expected, 3= as expected, 4= worse than expected, 5=much worse than expected)	3 months	Livesey 2009	2,3
Discomfort (pain in the past 48 hrs)	-	Patient reported	0 to 10 cm VAS	2-3 & 7-10 days post-op	Michie 1994	1
Discomfort (pain on removal of the dressing	-	Patient reported	0 to 10 cm VAS	2-3 & 7-10 days post-op	Michie 1994	1
Overall comfort	-	Patient reported	0 to 10 cm VAS	2-3 & 7-10 days post-op	Michie 1994	1
Wound itching (in the past 48hrs)	-	Patient reported	0 to 10 cm VAS	2-3, 7-10 days, 4 weeks, 7 months post-op	Michie 1994	1
Wound pulling (in the past 48 hrs)	-	Patient reported	0 to 10 cm VAS	2-3, 7-10 days, 4 weeks, 7 months post-op	Michie 1994	1
Conformability of the dressing to the wound	-	Surgeon reported	4 point rating scale (excellent, good, fair, poor)	7-10 days post-op	Michie 1994	1
Ability to contain exudate	-	Surgeon reported	4 point rating scale (excellent, good, fair, poor)	7-10 days post-op	Michie 1994	1
Ability to protect the wound	-	Surgeon reported	4 point rating scale (excellent, good, fair, poor)	7-10 days post-op	Michie 1994	1
Ability to facilitate mobility	-	Surgeon reported	4 point rating scale (excellent, good, fair, poor)	7-10 days post-op	Michie 1994	1
Ability to facilitate personal hygiene	-	Surgeon reported	4 point rating scale (excellent, good, fair, poor)	7-10 days post-op	Michie 1994	1
Overall impression of the incision	-	Patient reported	0 to 10 cm VAS	2-3 & 7-10 days post-op	Michie 1994	1
Evaluation of resulting scar	Pigmentation, scar colour, prescence of inflammation, suppleness/pliability,	Patient and surgeon reported	modified Vancouver Burn Assessment Scale (0 to 3 score)	4 weeks and 7 months post-op	Michie 1994	1

* 1= Cochrane 2011 dressings review; 2= Cochrane 2014 tissue adhesive review; 3=Chow 2010 tissue adhesive review; 4=additional studies provided by authors of the Cochrane dressings review update

Outcome as described by author	Wording used to measure outcome (where reported)	Who reported the outcome	Rating/measurement scale	Assessment time point	Study reference	Study source *
	scar height/evenness with the surrounding skin					
Ease of dressing application	-	Surgeon reported	Yes/No	2-3 & 7-10 days post-op	Michie 1994	1
Ease of dressing removal	-	Surgeon reported	Yes/somewhat difficult	2-3 & 7-10 days post-op	Michie 1994	1
Cosmetic result	-	Surgeon reported	4 point rating scale (excellent, good, fair, poor)	7-10 days post-op	Michie 1994	1
Infection	-	Surgeon reported	Unspecified	2-3 & 7-10 days post-op	Michie 1994	1
Pain upon palpation of the wound	-	Surgeon reported	3 point scale including Somewhat/no	7-10 days post-op	Michie 1994	1
Overall wound aspect	-	Surgeon reported	3 point scale including Excellent/good	7-10 days post-op	Michie 1994	1
Overall recovery	-	Surgeon reported	3 point scale including Excellent	7-10 days post-op	Michie 1994	1
Presence of small stitch abscess	-	Surgeon reported	Yes/no	7-10 days post-op	Michie 1994	1
Satisfaction with wound cosmesis	-	Parent reported	100mm VAS	2 to 3 weeks and 3 months follow up assessment (although unable to complete latter assessment as only 9 patients returned)	Ong 2002	2,3
Level of satisfaction with early postoperative management of the wound (regarding requirement of a return visit for medications, the possibility to wash oneself, the stuture removal)	-	Patient reported (verbal)	Numerical scale 0 to 10 (0-4, poor; 5-6, mild;7-8, good; 9-10, excellent)	15 days, 1, 3, 6 and 12 months	Pronio 2011	2

* 1= Cochrane 2011 dressings review; 2= Cochrane 2014 tissue adhesive review; 3=Chow 2010 tissue adhesive review; 4=additional studies provided by authors of the Cochrane dressings review update

Outcome as described by author	Wording used to measure outcome (where reported)	Who reported the outcome	Rating/measurement scale	Assessment time point	Study reference	Study source *
Discomfort	-	Unspecified	Unspecified	15 days, 1, 3, 6 and 12 months	Pronio 2011	2
Pain	-	Unspecified	Unspecified	15 days, 1, 3, 6 and 12 months	Pronio 2011	2
Pain	Pain resulting from dressing usage and not including mobilisation	Patient reported	Yes/no, 10 point VAS (0=no problems, 10=unbearable problems)	At dressing removal (mean 6 and 7 days)	Ravnskog 2001	4
Itching	-	Patient reported	Yes/no, 10 point VAS (0=no problems, 10=unbearable problems)	At dressing removal (mean 6 and 7 days)	Ravnskog 2001	4
Burning	Burning pain refering solely to a dressing-related sensation felt under the dressing	Patient reported	Yes/no, 10 point VAS (0=no problems, 10=unbearable problems)	At dressing removal (mean 6 and 7 days)	Ravnskog 2001	4
Discomfort during use of dressing	-	Patient reported	Yes/no, 10 point VAS (0=no problems, 10=unbearable problems)	At dressing removal (mean 6 and 7 days)	Ravnskog 2001	4
Pain at dressing removal	-	Patient reported	Yes/no, 10 point VAS (0=no problems, 10=unbearable problems)	At dressing removal (mean 6 and 7 days)	Ravnskog 2001	4
Skin damage (erythema, blisters or skin injury)		Observer reported	Small; 1-2cm, medium; 2-5cm, large<5cm	during hospital stay	Ravnskog 2001	4
Satisfaction with the cosmetic result	-	Patient reported	Dichotomous (satisfaied/dissatisfied)	Day 10 and day 90	Romero 2011	2
Pain at port sites	-	Surgeon reported	Unspecified	Day 10 and day 90	Romero 2011	2
Satisfaction with cosmetic result	-	Patients were asked by the senior dermatologist	1 (very satisfied) to 5 (not satisfied)	1 year post-op	Shamiyeh 2001	2,3
Degree of pain	-	Patient reported	Scale, 1-10	3 months post-op	Sniezek 2007	2,3
Ease of managing the surgical wound	-	Patient reported	Scale, 1-10	3 months post-op	Sniezek 2007	2,3

* 1= Cochrane 2011 dressings review; 2= Cochrane 2014 tissue adhesive review; 3=Chow 2010 tissue adhesive review; 4=additional studies provided by authors of the Cochrane dressings review update

Outcome as described by author	Wording used to measure outcome (where reported)	Who reported the outcome	Rating/measurement scale	Assessment time point	Study reference	Study source *
Ability to take a shower	-	Patient reported	Scale, 1-10	3 months post-op	Sniezek 2007	2,3
Overall satisfaction	-	Patient reported	Scale, 1-10	3 months post-op	Sniezek 2007	2,3
Cosmetic appearance	-	Patient reported	Scale, 1-10	3 months post-op	Sniezek 2007	2,3
Comfort of the dressing (discomfort at mobilization)	-	Patient reported	3 point scale (no discomfort at all, minor problems, severe discomfort)	Daily for 4 days after surgery	Vogt 2007	1
Comfort of the dressing (pain at dressing changes)	-	Patient reported	3 point scale (no discomfort at all, minor problems, severe discomfort)	Daily for 4 days after surgery	Vogt 2007	1
Comfort of the dressing (skin problems)	-	Patient reported	3 point scale (no discomfort at all, minor problems, severe discomfort)	Daily for 4 days after surgery	Vogt 2007	1
Signs of infection - redness, tenderness, swelling, exudates	-	Observer reported	Unspecified	2 weeks post-op	Vogt 2007	1
Wound complications - haematoma or persistent lymph oozing, surgical revision	-	Observer reported	Unspecified	2 weeks post-op	Vogt 2007	1
Length of hospital stay	-	Observer reported	n/a	n/a	Vogt 2007	1
Number of dressing changes	-	Observer reported	n/a	during postoperative stay	Vogt 2007	1
Patient comfort (difficulty in removing the dressings)	-	Nurse reported	Unspecified	Day 5	Wikblad 1995	1
Patient comfort (pain at dressing removal)	-	Nurse reported	3 point scale from "no pain at all" to "very painful"	Day 5	Wikblad 1995	1
Number of bandage changes	-	Nurse reported	n/a	Day 1 to day 5	Wikblad 1995	1
Reason for bandage changes	-	Nurse reported	n/a	Day 1 to day 5	Wikblad 1995	1

* 1= Cochrane 2011 dressings review; 2= Cochrane 2014 tissue adhesive review; 3=Chow 2010 tissue adhesive review; 4=additional studies provided by authors of the Cochrane dressings review update

Outcome as described by author	Wording used to measure outcome (where reported)	Who reported the outcome	Rating/measurement scale	Assessment time point	Study reference	Study source *
Effectiveness (wound healing)	-	Independent raters judging photograph	1=well healed (wound edges well together; a gap of <5% length of the incision allowed with no or slight redness), 2=partially healed (gaps >5% but <20% with slight to excessive redness), 3=poorly healed (gaps>20% with excessive redness)	Day 5 and 4 weeks after surgery	Wikblad 1995	1
Redness	-	Independent raters judging photograph	0=no redness, 1=slight redness, 2= excessive redness	Day 5 and 4 weeks after surgery	Wikblad 1995	1
Wound healing	Do you think the wound is well/partially/poorly healed?	Patient reported	3 point scale	Once a week after discharge for 3 weeks	Wikblad 1995	1
Skin changes	-	Patient reported	Unspecified	Once a week after discharge for 3 weeks	Wikblad 1995	1
Redness	Is the wound red?	Patient reported	Yes/No	Once a week after discharge for 3 weeks	Wikblad 1995	1
Swelling	Does the wound look swollen?	Patient reported	Yes/No	Once a week after discharge for 3 weeks	Wikblad 1995	1
Itching	Does the wound itch?	Patient reported	Yes/No	Once a week after discharge for 3 weeks	Wikblad 1995	1
Skin changes (erythema and blisters)	-	Independent raters judging photograph	n/a	Day 5	Wikblad 1995	1
Clinical utility (ability to allow ongoing evaluation of the incision)	How well the incision could be seen through the dressing	Nurse reported	1=good, 2=partially, 3=not atall	Day 1 to day 5	Wikblad 1995	1
How much the dressing had loosened	-	Nurse reported	graded scale from 1 to 3	Day 1 to day 5	Wikblad 1995	1
Treatment with antibiotics	-	Nurse reported	yes/no	4 weeks after surgery	Wikblad 1995	1

* 1= Cochrane 2011 dressings review; 2= Cochrane 2014 tissue adhesive review; 3=Chow 2010 tissue adhesive review; 4=additional studies provided by authors of the Cochrane dressings review update

Outcome as described by author	Wording used to measure outcome (where reported)	Who reported the outcome	Rating/measurement scale	Assessment time point	Study reference	Study source *
Safety (presence of infection - wound culture)	-	Clinical sample	n/a - lab sample	Day 5	Wikblad 1995	1
Dressing awareness	How aware are you of your dressing most of the time?	Patient reported	10 cm visual analogue scale with three anchors at 0,5 and 10cm	Day 1 to day 5	Wynne 2004	1
Movement limitation	Does the dressing limit you in moving about?	Patient reported	10 cm visual analogue scale with three anchors at 0,5 and 10cm	Day 1 to day 5	Wynne 2004	1
Comfort with removal	How comfortable do you feel during dressing changes?	Patient reported	10 cm visual analogue scale with three anchors at 0,5 and 10cm	Day 1 to day 5	Wynne 2004	1
Overall satisfaction	How satisfied overall do you feel with your dressing?	Patient reported	10 cm visual analogue scale with three anchors at 0,5 and 10cm	Day 1 to day 5	Wynne 2004	1
Wound healing - approximation	-	Observer reported	4 categories (total, partial;<2cm of superficial separation, moderate;>2cm of superficial separation, dehisced; complete separation of layers)	Day 1 to day 5	Wynne 2004	1
Wound healing - skin itegrity	-	Observer reported	3 categories (normal; pink no redness, inflammed; heat redness swelling, macerated within a 2.5cm border of the incision)	Day 1 to day 5	Wynne 2004	1
Wound infection	-	Observer reported	CDC criteria	unspecified	Wynne 2004	1
Dressing integrity	-	Observer reported	3 catergories - suture line exposed, poorly sealed, well sealed	unspecified	Wynne 2004	1
Experience with wound	Has your chest wound healed?	Patient reported	Yes/No	One month post-discharge	Wynne 2004	1
Antibiotic therapy	Has your doctor given you any antibiotics for	Patient reported	Yes/No	One month post-discharge	Wynne 2004	1

* 1= Cochrane 2011 dressings review; 2= Cochrane 2014 tissue adhesive review; 3=Chow 2010 tissue adhesive review; 4=additional studies provided by authors of the Cochrane dressings review update

Outcome as described by author	Wording used to measure outcome (where reported)	Who reported the outcome	Rating/measurement scale	Assessment time point	Study reference	Study source *
	your chest wound, since you left hospital?					
Experience with wound	Over the past month, has there been any fluid/discharge oozing from the chest wound? If so, how would you describe the fluid: watery, straw coloured; blood stained; pus (think yellow)	Patient reported	Yes/No	One month post-discharge	Wynne 2004	1
Experience with wound	Was a dressing required on your wound?	Patient reported	Yes/No	One month post-discharge	Wynne 2004	1
Experience with wound	Have you had any of the following problems with your chest wound? Redness, swelling, pain, tenderness	Patient reported	Yes/No	One month post-discharge	Wynne 2004	1
Experience with wound	Has your local doctor told you at any time your chest wound was infected?	Patient reported	Yes/No	One month post-discharge	Wynne 2004	1

* 1= Cochrane 2011 dressings review; 2= Cochrane 2014 tissue adhesive review; 3=Chow 2010 tissue adhesive review; 4=additional studies provided by authors of the Cochrane dressings review update
