# Supplementary material for: Developing outcome measures assessing wound management and patient experience: a mixed methods study
Source: BMJ Open. 2017 Nov 26;7(11):e016155. doi: 10.1136/bmjopen-2017-016155 (PMC5719294; doi:10.1136/bmjopen-2017-016155)
Supplement: Supplementary file 5 [file bmjopen-2017-016155supp005.pdf]

Note: Permission to use these measures must be obtained from the author (daisy.elliott@bristol.ac.uk). Work is being conducted to test the reliability, validity and sensitivity of these measures.

Bluebelle study

Bluebelle dressing allocation:	***Simple dressing***
Study ID:	
Participant name:	
Date of surgery:	
Date completed:	

Wound Experience Questionnaire

We are interested in how your wound(s) have healed since your operation and your experience of having a dressing, as part of the Bluebelle study. Please complete this short questionnaire yourself. You can complete the questionnaire as soon as you feel ready, but ideally this will be within four days of having your operation. If there is more than one wound, please respond **thinking about just one wound** – either the main one or another wound if there have been any concerns about how it has been healing.

When you have completed the questionnaire, please return it in the pre-paid envelope provided.

Section 1: Wound comfort

		Not at all	A little	Quite a bit	A lot
1.	Has your wound been itchy?	☐	☐	☐	☐
2.	Has your wound been painful?	☐	☐	☐	☐
3.	Has your wound had a pulling sensation?	☐	☐	☐	☐
4.	Has your wound felt tight?	☐	☐	☐	☐
5.	Has your wound been smelly?	☐	☐	☐	☐

Section 2: Removing the dressing

		Yes	No	
6.	Has the original dressing been removed/come off on its own?	☐	☐ →	*If "No" go to Section 3, Question 7*

If "Yes", how did it come off?

		Yes
a)	A doctor/nurse/other health professional	☐
b)	You/your partner/friend/family member	☐
c)	It came off on its own	☐

		Not at all	A little	Quite a bit	A lot
d)	Did you feel any pain when the dressing was removed?	☐	☐	☐	☐
e)	Did you feel any anxiety when the dressing was removed?	☐	☐	☐	☐

Section 3: Experience of having a dressing

		Not at all	A little	Quite a bit	A lot
7.	Has your dressing prevented you from showering or washing?	☐	☐	☐	☐
8.	Has your wound felt protected?	☐	☐	☐	☐
9.	Have you felt any anxiety about your wound in relation to your dressing(s)?	☐	☐	☐	☐
10.	Are you satisfied with your dressing(s)?	☐	☐	☐	☐

Additional comments:

……

……

……

Thank you for completing this questionnaire.

Note: Permission to use these measures must be obtained from the author (daisy.elliott@bristol.ac.uk). Work is being conducted to test the reliability, validity and sensitivity of these measures.

**Bluebelle dressing allocation:**	***Simple dressing***
**Study ID:**	
**Participant name:**	
**Date of surgery:**	
**Date completed:**	
**Completed by *(please tick)*:**	☐ Healthcare professional<br>☐ Participant<br>☐ Other (state):

Wound Management Questionnaire

To be completed by a healthcare professional up to 4 days after surgery

Or

To be completed by the participant up to 4 days after surgery if the participant is discharged before completion by a healthcare professional

If there is more than one wound, please respond **thinking about just one wound** – either the main one or another wound if there have been any concerns about how it has been healing. When you have completed the questionnaire, please return it in the pre-paid envelope provided.

Section 1: Wound leakage

In the past 24 hours…

		Not at all	A little	Quite a bit	A lot
1.	Has fluid from the wound leaked through the dressing?	☐	☐	☐	☐

If "Not at all", go to Section 2, Question 3

		Yes	No	If "Yes", how many times?
2.	Has the leakage required bedding or clothes to be changed?	☐	☐	____________

Section 2: Dressings

In the past 24 hours…

		Yes	No	
3.	Has the original dressing been replaced?	☐	☐	If "No", questionnaire is complete
	If "Yes", how many times?	__________		

4. Why was the dressing replaced?

		Yes (tick all that apply)	
a)	Routine change	☐	
b)	The dressing was saturated	☐	
c)	The wound was irritated	☐	
d)	The wound was blistered	☐	
e)	Another reason	☐	If "Yes", please specify what the reason was ________________________________

Additional comments:

……

……

……

Thank you for completing this questionnaire.
