# Supplementary material for: Developing outcome measures assessing wound management and patient experience: a mixed methods study
Source: BMJ Open. 2017 Nov 26;7(11):e016155. doi: 10.1136/bmjopen-2017-016155 (PMC5719294; doi:10.1136/bmjopen-2017-016155)
Supplement: Author's manuscript [file bmjopen-2017-016155.draft_revisions.pdf]

BMJ Open is committed to open peer review. As part of this commitment we make the peer review history of every article we publish publicly available.

When an article is published we post the peer reviewers' comments and the authors' responses online. We also post the versions of the paper that were used during peer review. These are the versions that the peer review comments apply to.

The versions of the paper that follow are the versions that were submitted during the peer review process. They are not the versions of record or the final published versions. They should not be cited or distributed as the published version of this manuscript.

BMJ Open is an open access journal and the full, final, typeset and author-corrected version of record of the manuscript is available on our site with no access controls, subscription charges or pay-per-view fees (http://bmjopen.bmj.com).

If you have any questions on BMJ Open's open peer review process please email info.bmjopen@bmj.com

BMJ Open

Development of outcome measures to assess practical wound management and symptoms associated with closed primary wounds after surgery

Journal:	*BMJ Open*
Manuscript ID	bmjopen-2017-016155
Article Type:	Research
Date Submitted by the Author:	06-Feb-2017
Complete List of Authors:	Elliott, Daisy; University of Bristol, School of Social and Community Medicine<br>Andronis, Lazaros; The University of Birmingham, Department of Health Economics<br>Blazeby, Jane; University of Bristol, Department of Social Medicine<br>Blencowe, Natalie; University of Bristol, Centre for Surgical Research, School of Social and Community Medicine<br>Calvert, Melanie; The University of Birmingham, Primary Care and General Practice<br>Coast, Joanna; University of Bristol, School of Social and Community Medicine<br>Draycott, Tim; Obstetrics & Gynaecology, North Bristol NHS Trust<br>Donovan, Jenny; University of Bristol, School of Social and Community Medicine<br>Gooberman-Hill, Rachael; University of Bristol, School of Clinical Sciences<br>Longman, Robert; University Hospitals Bristol, Dept of Coloproctology<br>Magill, Laura ; Queen Elizabeth Hospital<br>Mathers, Jonathan; University of Birmingham, Public Health<br>Pinkney, Tom; University of Birmingham, Centre for Patient Reported Outcomes Research<br>Reeves, Barnaby; University of Bristol, Bristol Heart Institute<br>rogers, chris; University of Bristol, Bristol Heart Institute<br>Rooshenas, Leila; University of Bristol, School of Social and Community Medicine<br>Torrance, Andrew; The University of Birmingham, Institute of Applied Health Research<br>Welton, Nicky; University of Bristol, School of Social and Community Medicine<br>Woodward, Mark; University Hospitals Bristol<br>Young, Trudie; Welsh Wound Innovation Centre<br>Dumville, Jo C.; University of Manchester, School of Nursing, Midwifery & Social Work<br>Flintoff, Louise; University Hospitals Bristol<br>Macefield, Rhiannon; University of Bristol, Department of Social Medicine<br>Messenger, David; University Hospitals Bristol<br>McMullan, Christel; University of Birmingham, Institute of Applied Health Research<br>Murkin, Charlotte; University of Bristol, School of Social and Community Medicine<br>van der Nelson, Helen; North Bristol NHS Trust, Obstetrics & Gynaecology

**Primary Subject Heading**:	Surgery
Secondary Subject Heading:	Qualitative research
Keywords:	QUALITATIVE RESEARCH, Wounds, Dressings, Outcome measures

SCHOLARONE™ Manuscripts

For peer review only

Development of outcome measures to assess practical wound management and symptoms associated with closed primary wounds after surgery

The Bluebelle Study Group*

Corresponding author: Daisy Elliott [daisy.elliott@bristol.ac.uk]

*The Bluebelle Study Group consists of the following sub-groups:

Bluebelle grant co-applicants: Lazaros Andronis[1], Jane Blazeby[2,3], Natalie Blencowe[2,3,] Melanie Calvert[4], Joanna Coast[2,7], Tim Draycott[6], Jenny L Donovan[2,7], Rachael Gooberman-Hill[8], Robert Longman[3], Laura Magill[9], Jonathan Mathers[4], Thomas Pinkney[5], [9], Barnaby C Reeves[10], Chris A Rogers[10], Leila Rooshenas[2,] Andrew Torrance[4], Nicky J Welton[2], Mark Woodward[3], Trudie Young[11]

Other members of the Bluebelle Study Group: Jo Dumville[12], Daisy Elliott[2], Louise Flintoff[3], Rhiannon Macefield[2], David Messenger[3], Christel McMullan[4], Charlotte Murkin[2,3], Helen van der Nelson[6]

1. Health Economics Unit, School of Health and Population Sciences, University of Birmingham, Birmingham, UK.

2. School of Social and Community Medicine, University of Bristol, Bristol, UK.

3. University Hospitals Bristol NHS Foundation Trust, Bristol, UK.

4. Institute of Applied Health Research, University of Birmingham, Birmingham, UK.

5. Centre for Patient Reported Outcomes Research, University of Birmingham

6. North Bristol NHS Trust, Bristol, UK.

7. NIHR Collaboration for Leadership in Applied Health Research and Care West at University Hospitals Bristol NHS Trust, Bristol, UK

8. Musculoskeletal Research Unit, School of Clinical Sciences, University of Bristol, UK.

9. Academic Department of Surgery, Queen Elizabeth Hospital, University of Birmingham, UK.

For peer review only

10. Clinical Trials and Evaluation Unit, School of Clinical Sciences, University of Bristol, Bristol, UK.

11. Welsh Wound Innovation Centre, Rhodfa Marics, Ynysmaerdy, Pontyclun, Rhondda Cynon Taf, Wales, UK.

12. School of Nursing, Midwifery & Social Work, University of Manchester, Manchester, UK.

Running title: Outcome measures for wound management and symptoms of primary surgical wounds

Keywords: Qualitative research; wounds; dressings; outcome measures

Word count: 2900 (excluding title page, abstract, tables and references)

For peer review only

ABSTRACT

Objectives: To develop outcome measures to assess practical wound management issues and symptoms associated with closed primary surgical wounds and dressings.

Design: Mixed methods, including cognitive interviews and literature searches.

Setting: Five UK hospitals.

Participants: Sixty-four patients and 15 health care professionals from abdominal general surgical specialities and obstetrics (caesarean section).

Methods: Measures were developed according to standard guidelines including a literature review and semi-structured interviews to identify issues relevant to patients' experiences of surgical wounds and dressings. These were written into provisional questionnaire items for a single outcome measure. Cognitive interviews with patients and health care professionals assessed face validity, acceptability and relevance. Findings from interviews were regularly shared with the study team who suggested amendments to modify and reword items to improve understanding before further iterative testing with patients and health care professionals.

Results: Analyses of existing literature and interviews produced a total of 69 issues related to practical issues and patient experiences of primary surgical wounds and dressings. Pre-testing and iterative revision established the need for two separate measures. One measure addresses health care professionals' experience of would management in two key areas: exudate and its impact, and allergic reactions to the dressing. The other measure addresses patients' experience of wounds in seven key areas: wound comfort, dressing removal, dressings to protect the wound, impact on daily activities, ease of movement, anxiety about the wound and satisfaction with dressing. Each measure took less than five minutes to complete and were understood and acceptable to patients and health care professionals.

Conclusion: This in-depth study has developed measures to assess practical wound management issues and symptoms and patient experience associated with primary surgical wounds to use for studies of wound dressings. Further work to test their validity and reliability and application to other settings is now required.

STRENGTHS AND LIMITATIONS OF THIS STUDY

- This is the first study to explore the important issues related to the practical management of primary surgical wounds and patient experience immediately following surgery.

For peer review only

- This study used robust methods to identify key issues of outcome that should be assessed in future randomised controlled trials of wound dressings. Interviews provided a rich account of the key factors that affected wound management and patient experience while a purposeful sampling strategy ensured that perspectives were captured from a range of participants. Data produced from the interviews were supplemented by an extensive literature search to ensure a comprehensive list of issues was considered.
- Future work is needed to test the reliability, validity and sensitivity to change of the new measures.

For peer review only

INTRODUCTION

An estimated 234 million major surgical procedures are undertaken every year worldwide [1]. It is common practice to apply dressings over the closed wound in adult surgery and many different dressing types are available [2]. Whilst the role of dressings in covering primary surgical wounds in reduction of rates surgical site infection (SSI) is unclear, it is recognised that they are important for patient comfort and management of wound exudate in the early post-operative period [3].

A recent Cochrane systematic review summarised data relating to wound dressings and risk of SSI in primary surgical wounds. No evidence was found to suggest that any type of dressing significantly reduced the risk of developing an SSI compared with leaving wounds exposed; neither was there any benefit associated with particular dressings [2]. The authors concluded that decision-making around dressings may need to be informed by costs and practical issues. The review highlighted that measures for assessment of wound cosmesis (in the longer term) are available [4, 5]. It reported, however, that measurement of wound issues such as exudate and patient experience is difficult because of a lack of well-developed and validated measurement tools.

In the past, outcome measures were often derived from clinicians' views, rather than patients', on issues of importance [6]. However, more recently, a patient-centred approach has been advocated [7]. This has resulted in the increasing use of patient-reported outcome measures (PROMs) in clinical trials and usual clinical practice [8]. The development of PROMs may include use of qualitative research methods that provide the opportunity to elicit and characterise patients' experiences of their health conditions and treatment. Qualitative methods can also define health care professionals' experiences of care and management. [9], which can be supplemented by expert input and studies in published literature [10]. This paper describes the development of measures to assess practical wound management issues, symptoms and patient experience associated with primary surgical wounds.

METHODS

Study design

Measures were developed according an existing framework for developing PROMs [11, 12], also incorporating guidance on eliciting health domain concepts using qualitative methodologies [9, 10]. The study is reported according to qualitative reporting guidelines

For peer review only

(See Additional File 1). Phase 1 aimed to produce a comprehensive list of potential issues relating to wound and dressing experience and practical management issues. Phase 2 developed issues identified from Phase 1 into questionnaire items. Phase 3 evaluated the measures for acceptability and relevance using cognitive interviews with patients and health care professionals. The final part of development (phase 4) consisted of psychometric testing and will be reported elsewhere. Phases 1 to 3 were overseen by a working group (DE, JB, LR, RM, CMM) that was part of the Bluebelle study management group. The management group met before and between each phase to discuss progress and make decisions about how the measures should be adapted. Ethical approval was provided by the NHS Health Research Authority NRES Committee London – Camden & Kings Cross (14/LO/0640). Written informed consent was provided by all participants.

Phase 1: Generation of relevant issues

1.1 Interviews

Participants were recruited as part of a wider feasibility study to explore whether a trial comparing different types of dressings, and dressing versus no dressings, is possible (The Bluebelle study: a feasibility study of three wound dressing strategies in eLective and unplanned surgery, HTA - 12/200/04 [14,15]. Eligible patients had undergone abdominal general surgery or caesarean section were identified and approached by research nurses and surgical trainees (NB, DM). DE, CMM and LR contacted interested patients to arrange interviews. A purposeful sampling strategy ensured that perspectives were captured from a range of participants [10]. Within this sampling approach, maximum variation was sought in relation to age, gender, ethnicity, type of surgery, dressing type and location.

Interviews were conducted with patients to explore and characterise experiences of wounds and dressings by LR, DE and CMM. A topic guide was developed (based on literature and views of health care professionals in the Bluebelle study team) to ensure that discussions covered the same core issues but with sufficient flexibility to allow new issues of importance to the informants to emerge (See Additional File 3).

Interviews were audio-recorded and transcribed in full. Transcripts were imported into NVivo (version 10). All data relating to outcomes and issues of importance to patients that were relevant to dressing use and the practical management of the wound in the initial time period post-surgery were assigned labels (coded) by DE or CMM. Data were analysed using techniques of constant comparison derived from grounded theory methodology, and

For peer review only

emerging codes across the dataset were then compared to look for shared or disparate views among participants [16]. A subset of approximately half of the interviews (n = 19) was double coded by a third researcher (LR) to highlight any differences in the interpretation of codes [9]. Data collection and analysis continued until the team were confident that saturation had been reached i.e. no more patterns or themes emerged from the data.

1.2 Literature search to identify existing tools

Three systematic reviews [17-19] were used to identify RCTs that included outcomes relating to wounds and dressings. Papers were scrutinised for outcomes relating to practical wound management or symptoms and patient experience. RM and JB extracted data including verbatim descriptions of these outcomes, and when and how they were measured.

1.3 Synthesis of literature and qualitative data

A list of issues from the analysis of the interviews and literature search was collated into an item tracking matrix [20]. DE, JB, LR, RM and CMM agreed on a set of words or phrases to reflect each issue and also noted additional phrasing made by participants in a subsequent column [9]. Issues which were conceptually similar were organised into categories. For instance, issues such as ‘itchiness/irritation’, ‘presence of pulling sensation’, and ‘tightness of wound’ were mapped into a ‘wound comfort’ category.

Phase 2: ‘Operationalisation’: Construction of a provisional measure

DE, JB, LR, RM and CMM used the item tracking matrix to agree which issues should be written into questionnaire items. Items featured words and phrases used by patients in the interviews to enhance content validity [10, 21].

Phase 3: Pre-testing

DE, CMM and CM conducted face-to-face cognitive interviews with a new sample of patients who had undergone surgery. Patients who had undergone abdominal general surgery or caesarean section at one of five hospitals in two cities in the UK were identified and approached by research nurses and surgical trainees (NB, DM). DE, CMM and CM contacted interested patients to arrange interviews. Health care professionals involved in post-surgical care from the participating hospitals trusts were approached directly by the qualitative team (DE, CMM, CM). As in Phase 1, sampling was purposeful to achieve maximum variation in relation to clinical role, age, gender and geographic location (for

For peer review only

health care professionals) and age, gender, ethnicity, type of surgery, dressing type and location (for patients).

Interviews explored the acceptability of the measure and coverage of patients and health care professionals' concerns (in terms of language, accuracy, and relevance) as well as layout [10]. During each interview, participants were asked to complete the measure by reading each item aloud and commenting on their understanding. Interviews were guided by a series of probes (e.g. 'What does this item mean to you?', 'Are there other ways you would describe it?'; [22]). Participants' body language was also observed and prompted further discussion about specific items (such as the participant nodding in agreement or frowning) [9]. A copy of the topic guide is available (See Additional File 3).

DE, CM and MMM maintained detailed field notes from each interview, describing suggestions for modifications and improvements to the provisional measures. Operationalisation and modification of the measures was an iterative process. Findings from cognitive interviews and suggestions for amendments were regularly disseminated to the Bluebelle Trial Management group (TMG), which consisted of a multidisciplinary group of health care professionals, including surgeons, health services researchers and research nurses. Each stage of feedback informed amendments to modify and reword items to improve understanding, which was repeated following efforts to revise questions and eliminate problems [22]. This process continued until no new issues were identified and no further refinements were believed to be necessary.

RESULTS

Phase 1: Generation of relevant issues

1.1 Interviews

Semi-structured face-to-face or telephone interviews were conducted between July 2014 and July 2015 (n= 39). Interviews lasted an average of 25 minutes (range = 15–50 minutes). The sample consisted of 24 women and 15 men, who mostly described themselves as white British (90%). They had a mean age of 56 years (range 41-88 years). Participants were recruited from five hospitals in two cities in the UK, and had either undergone abdominal general surgery (74%) or a caesarean section (26%). Participant demographics for Phase 1 interviews are shown in Table 1.

For peer review only

Table 1: Demographics of participants' interviews in Phases 1 and 3

		Phase 1: Generation of relevant issues	Phase 3: Pre-testing	
**Qualitative interviews (n = 79)**		39 patients	25 patients	15 health care professionals
**Age (years)**	Range	22-88	19-76	23-60
	Mean	56	54	41
**Sex**	Female	27	12	13
	Male	12	13	2
**Ethnicity**	White	35	22	14
	Asian	1	1	0
	African	2	1	1
	Indian	1	0	0
	Filipino	0	1	0
**Type of surgery**	Abdominal	32	25	15
	Obstetric	6	0	0
**Dressing type**	Tissue adhesive	7	5	-
	Adhesive	32	18	-
	No dressing	0	2	-
**Location**	Bristol	28	15	9
	Birmingham	11	10	6

1.2 Literature search to identify existing tools

Twenty six studies that included outcomes relating to wounds and dressings were identified [23-48]. Only two studies included a validated instrument to assess outcomes [25, 39]. These were for long term scarring [4] and cosmesis [5]. However, no studies reported using validated measures relating to issues associated with practical wound management and patient experiences in the early post-operative period. Overall, papers provided very little detail on the rationale for including each outcome and how these outcomes had been measured. Attempts were made to contact the authors for more information, with only one reply.

1.3 Synthesis of literature and qualitative data

When describing experiences in the interviews, patients commented on several factors that affected perceptions of how well their wound was healing, including how it felt (tightness, pain, and itchiness) and whether any fluid had leaked from the wound. The literature review showed these issues had briefly been captured in some previous (unvalidated) outcome measures.

All patients had at least one dressing applied after surgery, although this varied between adhesive coverings (absorptive or non-absorptive) and tissue adhesive as-a-dressing ('tissue glue'). Both the interviews and existing outcomes from the literature highlighted that

For peer review only

dressings multiple practical issues around dressing use (including ability to contain exudate and ease of removal). The interviews also demonstrated that there were psychological factors that affected dressing experience and satisfaction (i.e. anxiety about cleanliness of the wound). In particular, patients in the interviews described potential benefits to having tissue glue as a dressing, including that it was transparent, waterproof, did not require multiple applications and came off naturally.

The interviews and the literature search produced a total of 69 issues. These were grouped into ten broad categories: wound comfort, exudate and its impact, allergic reactions to the dressing, dressing key removal, dressings to protect the wound, impact on daily activities, ease of movement, anxiety about the wound, satisfaction with dressing and wound appearance. Table 2 provides illustrative quotes for the categories identified.

Table 2: Categories identified

Category	Example quote
**Wound comfort**	*"I've now got really itchy where the plaster goes. Which is uncomfortable."* (Patient, adhesive dressing)
**Exudate and its impact**	*"If I walked around it would get really damp. I mean it would soak my pyjamas and drip down my legs. It was quite manky really…. Then they would put a sort of big, well, like a big plaster on top of that, and then they put a kind of absorbent pad over that, to absorb some of that liquid."* (Patient, adhesive dressing)
**Reactions to the dressing**	*"I was allergic to the surgical tape."* (Patient, adhesive dressing)
**Dressing removal**	*"I just completely soaked it [adhesive dressing] in the shower then my husband just took it off for me. But it was, it was really easy. Much easier than I thought."* (Patient, adhesive dressing)
**Wound protection**	*"I'd be worried about catching it [the wound], knocking it, or something getting in so that it became infected."* (Patient, adhesive dressing)
**Impact on daily activities**	*"With the glue [dressing] it's easy to shower. With a [adhesive] dressing it wouldn't be so easy to shower and you'd be worried."* (Patient, tissue adhesive dressing)
**Ease of movement**	*"What I do find is the dressings are a bit constricting, especially as I get a bit better because they don't turn with your body so easily and then I feel that it makes me feel more constricted."* (Patient, tissue adhesive dressing)
**Anxiety about the wound**	*"You could catch things just from the air. That made me think, "Well, you'd need something to kind of protect it."* (Patient, adhesive dressing)
**Satisfaction with dressing**	*"Glue [as a dressing] requires no maintenance. I was very pleased. You don't have to change it you just leave it alone … I think that helps with the healing process physically and mentally."* (Patient, tissue adhesive dressing)
**Wound appearance**	*"If it was red and inflamed I would have thought, "Something has gone wrong with it."* (Patient, adhesive dressing)

Phase 2: 'Operationalisation': Construction of a provisional measure

A provisional measure was designed based on the findings from Phase 1. Nine key categories were included: wound comfort, exudate and its impact, allergic reactions to the dressing, dressing removal, dressings to protect the wound, impact on daily activities, ease

For peer review only

of movement, anxiety about the wound and satisfaction with dressing. The complete item tracking matrix (including the issues and categories identified) is available in the online appendix (see Additional File 2). Issues relating to the appearance of the wound were not included as they were only relevant to longer term outcomes of wound healing (not within first days of surgery). Additionally, since most patients reported having an adhesive dressing, many had not seen their wound within this timeframe. The first version of the measure included 16 items, and was provisionally called the Practical Wound Management Questionnaire.

Phase 3: Pre-testing

Cognitive interviews (n = 40) were conducted between July 2015 and March 2016 by DE, CMM and CM. This consisted of 25 patients who were in hospital and had undergone abdominal general surgery, and 15 health care professionals involved in surgical wound care. Participants were recruited from two hospitals in two UK cities. Demographics are shown in Table 1.

Interviews highlighted issues with content in the initial measure. For example, items regarding the colour of the wound exudate were removed. Questions were rephrased to focus on the experience of having a dressing rather than general recovery after surgery (i.e. '*Have you been able to perform everyday tasks? (i.e. showering/bathing)*' was changed to '*Has your dressing prevented you from showering/washing?*'). Additionally, since four patients commented that the smell of their wound was missing on the measure, an item was added to capture this.

The measure had intended to be administered two days after surgery, although feedback suggested that this needed to be completed up to day four as the patient may be disorientated from surgery in the first few days. However, since there were clear differences in recovery with caesarean section and abdominal surgery patients, a timeframe of within four days of surgery was set, and the measures recorded the date of surgery and date completed to determine context of responses.

Feedback from patients suggested it was difficult to respond to questions about exudate, since a healthcare professional cared for their wound whilst they were in hospital. If their dressing had been changed, they were also uncertain about the reason why (i.e. simply as part of standard practice or for other reasons). Therefore, the study team decided to separate the

For peer review only

measure into two separate measures. The first related to the practical aspects of wound management and the second related to the patient's experience of the wound/dressing and the psychological aspects (anxiety, satisfaction etc). The two measures were named the Wound Management and the Wound Experience Questionnaires respectively.

Seven versions of measures were modified throughout the pre-testing phase. Pre-testing continued until no new issues were identified and no further refinements were believed to be necessary. The final version of The Wound Management Questionnaire contains four items, whilst The Wound Experience Questionnaire contains 10 items. Overall, the final versions of the measures were well received. In addition, 96% of participants stated that each measure took less than five minutes to complete.

DISCUSSION

Studies evaluating the effectiveness of dressings on primary surgical wounds typically focus on SSI as a primary outcome. However, Cochrane reviews in this area have emphasised the need for assessment of other important outcomes such as practical wound management issues and patient experience of wound dressings. This study therefore has explored these important issues in patients with closed primary surgical wounds. The Wound Management Questionnaire assesses practical issues early after surgery for completion by health care professionals and The Wound Experience Questionnaire assesses symptoms and patient satisfaction with their wound and dressings. Final versions of the measures were easily completed and acceptable to patients and health care professionals and both are now ready for full psychometric testing to establish their measurement properties.

Analyses of three systematic reviews [16-18] identified RCTs that included outcomes relating to wounds and dressings. Two validated tools were identified although neither are relevant in the early post-operative phase and both focussed on cosmesis and scar appearance [4, 5]. No validated measures for assessing early wound issues or patient experience were identified. In addition, there was very little detail of how the items were developed and it was not apparent that patient input had been sought. True patient-centred outcome measures require full consideration of patients' experiences and views' [7, 8].

The main strength of this research, therefore, is the use of qualitative research methods to provide important insights into the under researched area of early issues related primary

For peer review only

surgical wounds relating to practical wound management and patient experience. Interviews provided a rich account of the key factors that affected wound recovery and satisfaction, and a purposeful sampling strategy ensured that perspectives were captured from a range of participants. To further enhance the reliability of the findings, the data were analysed by multiple researchers [9]. Data produced from the interviews were supplemented by an extensive literature search to ensure a comprehensive list of issues were initially generated, and therefore acted as a method of triangulation to increase the plausibility and dependability of the interview data [10].

It is important to note that these measures have only been pre-tested in relation to primary surgical wounds. Wounds that are intentionally left open or wounds that have developed problems are likely to require dressings that have advanced practical properties that are tailored to the wound requirements [14]. Although participants were purposefully sampled, most had had a dressing of some kind (94%). A prospective real-time survey of dressings has demonstrated that this reflects current practice [15]. In addition, these measures have only been pre-tested in relation to abdominal surgical wounds. However, characteristics of wound healing in this area are likely to be consistent with many other parts of the body. Furthermore, these measures focus specifically on the experience of dressings - methods of wound closure (i.e. potentially leading to differential ease of removal of sutures or staples) may also affect patient experience, although this would require further investigation.

In summary, the Wound Management Questionnaire and Wound Experience Questionnaire can be completed both by patients and by health care professionals responsible for post-operative wound care. Taken together, these developed measures provide important insights into wound management and patient experience in relation to primary surgical wounds. These measures will now be further developed to ensure they are appropriate and psychometrically-tested instruments, with a view to informing decision-making around dressings and use in clinical trials.

REFERENCES

1. Weiser TG, Regenbogen SE, Thompson KD, et al. An estimation of the global volume of surgery: a modelling strategy based on available data. Lancet 2008; 372: 139–44.
2. Dumville J, Gray T, Walter C, et al. Dressings for the prevention of surgical site infection. Cochrane Database of Systematic Reviews 2014, Issue 9. Art. No.: CD003091.

For peer review only

3. Walter CJ, Dumville JC, Sharp CA, et al. Systematic review and meta-analysis of wound dressings in the prevention of surgical-site infections in surgical wounds healing by primary intention. British Journal of Surgery 2012;9(9):1185–1194.
4. Sullivan T, Smith J, Kermode J, McIver E, Courtemanche DJ. Rating the burn scar. J Burn Care Rehabil 1990; 11: 256-60.
5. Hollander JE, Singer AJ, Valentine S, et al. Wound registry: development and validation Ann Emerg Med 1995. 25: 675–685.
6. Wilburn J, McKenna SP, Twiss J et al. Assessing quality of life in Crohn's disease: development and validation of the Crohn's Life Impact Questionnaire (CLIQ). Qual Life Res 2015. 24:2279–2288.
7. Trujols J, Portella MJ, Iraurgi I, et al. Patient-reported outcome measures: Are they patient-generated, patient-centred or patient-valued? J Ment Health 2013. 22:6, 555-562.
8. Bausewein C, Simon ST, Benalia H, et al. Implementing patient reported outcome measures (PROMs) in palliative care–users' cry for help. Health Qual Life Outcomes 2011. 9: 27.
9. Patrick DL, Burke L, Gwaltney, CJ et al. Content validity—establishing and reporting the evidence in newly-developed patient-reported outcomes (PRO) instruments for medical product evaluation: ISPOR PRO good research practices task force report: Part I – eliciting concepts for a new PRO instrument. Value Health 2011. 14: 967–977
10. Patrick DL, Burke L, Gwaltney CJ, et al. Content validity—establishing and reporting the evidence in newly developed patient-reported outcomes (PRO) instruments for medical product evaluation: ISPOR PRO good research practices task force report: Part 2—assessing respondent understanding. Value Health 2011;14:978–88.
11. Lasch KE, Marquis P, Vigneux M, et al. PRO development: rigorous qualitative research as the crucial foundation. Qual Life Res 2010. 19:1087–1096.
12. Sprangers MAG, Cull A, Bjordal K, et al. The European Organisation for Research and Treatment of Cancer approach to quality of life assessment: guidelines for developing questionnaire modules. Qual Life Res 1993, 2, 287–295.
13. Sprangers MAG, Cull A, Groenvold M, et al. The European Organisation for Research and Treatment of Cancer approach to developing questionnaire modules: an update and overview. Qual Life Res 1998, 7, 291–300.
14. Rooshenas, L.B.S.G., the Severn and Peninsula Audit and Research Collaborative for Surgeons, and the West Midlands Research Collaborative. Bluebelle study (phase A): a mixed-methods feasibility study to inform an RCT of surgical wound dressing strategies. BMJ Open, 2016. 6:e012635.
15. Severn, A. Peninsula, S. Research Collaborative for,G. West Midlands Research Collaborative on behalf of the Bluebelle Study. Feasibility work to inform the design of a randomized clinical trial of wound dressings in elective and unplanned abdominal surgery. Br J Surg, 2016. Aug 4.
16. Strauss A, Corbin J. Grounded theory methodology In: Denzin NK LY, editor. Handbook of Qualitative Research. London: Sage; 1994.
17. Dumville JC, Gray TA, Walter CJ, Sharp CA, Page T. Dressings for the prevention of surgical site infection. The Cochrane database of systematic reviews. 2014;9:CD003091.
18. Dumville JC, Coulthard P, Worthington HV, et al. Tissue adhesives for closure of surgical incisions. Cochrane Database Syst Rev 2014;11:CD004287.

19. Chow A, Marshall H, Zacharakis E, Paraskeva P, Purkayastha S. Useof tissue glue for surgical incision closure: a systematic meta-analysis of randomized controlled trials. J Am Coll Surg 2010. 211:114–25.
20. US Food and Drug Administration Guidance for Industry: Patient-Reported Outcome Measures: Use in Medical Product Development to Support Labeling Claims. Rockville, MD: Department of Health and Human Services, Food and Drug Administration, Center for Drug Evaluation and Research, 2009.
21. Leidy NK, Vernon M. Perspectives on patient-reported outcomes: content validity and qualitative research in a changing clinical trial environment. Pharmacoeconomics 2008;26:363–70.
22. Beatty PC, Willis GB. Research synthesis: The practice of cognitive interviewing. Public Opinion Quarterly 2007. 71:287–311.
23. Amin M, Glynn F, Timon C. Randomized trial of tissue adhesive vs staples in thyroidectomy integrating patient satisfaction and Manchester score. Otolaryngol Head Neck Surg 2009;140: 703–708.
24. Avsar A, Ustunnner I, Keskin L, Ozturk O, Tas E. 2-octylcyanoacrylate tissue adhesive versus polypropylene suture for skin closure of Pfannenestiel incision. Trk Jinekoloji ve Obstetrik Derne i Dergisi 2009;6:117–22.
25. Blondeel PN, Murphy JW, Debrosse D, et al. Closure of long surgical incisions with a new formulation of 2-octylcyanoacrylate tissue adhesive versus commercially available methods. Am J Surg 2004. 188:307–313.
26. Burke NG, Green C,McHugh G,McGolderick N, Kilcoyne C, Kenny P. A prospective randomised study comparing the jubilee dressing method to a standard adhesive dressing for total hip and knee replacements. J Tissue Viability 2012;21:84–87.
27. Chibbaro S, Tacconi L. Use of skin glue versus traditional wound closure methods in brain surgery: a prospective, randomized, controlled study. Journal of Clinical Neuroscience 2009;16(4):535–9.
28. Cosker T, Elsayed S, Gupta S, Mendonca AD, Tayton KJ. Choice of dressing has a major impact on blistering and healing outcomes in orthopaedic patients. Journal of Wound Care 2005;14(1):27–9.
29. Dowson C, Gilliam A, Speake W, Lobo D, Beckingham. A prospective, randomised controlled trial comparing n-butyl cyanoacrylate tissue adhesive (Liquiband) with sutures for skin closure after laparoscopic general surgical procedures. Surgical Laparoscopy, Endoscopy and Percutaneous Techniques 2006;16(3):146–50.
30. Gennari R, Rotmensz N, Ballardini B, et al. A prospective, randomized, controlled clinical trial of tissue adhesive (2-octylcyanoacrylate) versus standard wound closure in breast surgery. Surgery 2004;136:593–599.
31. Greene D, Koch RJ, Goode RL. Efficacy of octyl-2-cyanoacrylate tissue glue in blepharoplasty. A prospective controlled study of wound-healing characteristics. Arch Facial Plast Surg 1999;1:292–296.
32. Holm C, Petersen JS, Gronboek F, Gottrup F. Effects of occlusive and conventional gauze dressings on incisional healing after abdominal wounds. European Journal of Surgery 1998;164:179–83.
33. Haider J. A prospective randomised controlled double masked multi-center clinical trial of medical adhesives for the closure of laparoscopic incisions. Journal of Minimally Invasive Gynecology 2014;21:252–8.

34. Keng TM, Bucknall TE. A clinical trial of tissue adhesive (histoacryl) in skin closure of groin wounds. Medical Journal of Malaysia 1989;44(2):122–8.
35. Khan RJK, Fick D, Yao F, Tang K, Hurworth M, Nivbrant B, et al. A comparison of three methods of wound closure following arthroplasty: a prospective randomised controlled trial. The Journal of Bone and Joint Surgery 2006;88:238–42.
36. Law NH, Ellis H. Exposure of the wound - a safe economy in the NHS. Postgraduate Medical Journal 1987;63:27–8.
37. Lawrentschuk N, Falkenburg MP, Pirpiris M. Wound blisters post hip surgery: a prospective trial comparing dressings. Australian and New Zealand Journal of Surgery 2002;72:716–9.
38. Livesey C, Wylde V, Descamps S, et al. Skin closure after total hip replacement: a randomised controlled trial of skin adhesive versus surgical staples. J Bone Joint Surg Br 2009;91:725–759.
39. Michie DD, Hugill JV. Influence of occlusive and impregnated gauze dressings on incisional healing: a prospective randomized, controlled study. Annals of Plastic Surgery 1994;32:57–64.
40. Ong CC, Jacobsen AS, Joseph VT. Comparing wound closure using tissue glue versus subcuticular suture for pediatric surgical incisions: a prospective, randomised trial. Pediatr Surg Int 2002; 18:553–555.
41. Pronio A, Di Fillippo A, Narillii P, Caporilli D, Vestri A, Ciamberlaon B, et al. Closure of cutaneous incision after thyroid surgery: a comparison between metal clips and cutaneous octyl-2-cyanoacrylate adhesive. A prospective randomized clinical trial. European Journal of Plastic Surgery 2011;34:103–10.
42. Shamiyeh A, Schrenk P, Stelzer T,Wayand WU. Prospective randomised blind controlled trial comparing sutures, tape, and octylcyanoacrylate tissue adhesive for skin closure after phlebectomy. Dermatologic Surgery 2001;27(10):877–80.
43. Sniezek P, Walling H, DeBloom III J, Messingham M, VanBeek M, Kreiter C, et al. A randomised controlled trial of high-viscosity 2-octyl cyanoacrylate tissue adhesive versus sutures in repairing facial wounds following Mohs micrographic surgery. Dermatological Surgery 2007;33(8): 966–71.
44. Ravenscroft MJ, Harker J, Buch KA. A prospective, randomised, controlled trial comparing wound dressings used in hip and knee surgery: Aquacel and Tegaderm versus Cutiplast. Annals of the Royal Collage of Surgeons of England 2006;88(1):18–22.
45. Romero P, Frongia G, Wingerter S, Holland-Cunz S. Prospective, randomized,controlled trial comparing a tissue adhesive (Dermabond™) with adhesive strips (Steri-Strips™) for the closure of laparoscopic trocar wounds in children. European Journal of Pediatric Surgery 2011;21(3): 159–62.
46. Vogt KC, Uhlyarik M, Schroeder TV. Moist wound healing compared with standard care of treatment of primary closed vascular surgical wounds: a prospective randomized controlled trial. Wound Repair and Regeneration 2007;15: 624–7.
47. Wikblad K, Anderson B. A comparison of three wound dressings in patients undergoing heart surgery. Nursing Research 1995;44(5):312–6.
48. Wynne R, Botti M, Stedman H, Holsworth L, Harinos M, Flavell O, et al.Effect of three wound dressings on infection, healing comfort, and cost in patients with sternotomy wounds: a randomised trial. Chest 2004;125(1):43–9.

ACKNOWLEDGEMENTS

We would like to thank all participants for giving up their time to take part in this study, and members of the Bluebelle study team for their helpful comments on the earlier drafts of the measures.

CONTRUBITIONS

All members of the Bluebelle Study Group read and commented on the final version of the paper. Other roles are described below.

Lazaros Andronis co-applicant in the Bluebelle study grant (Health Economics); **Jane Blazeby** Chief investigator of the Bluebelle study grant (NIHR Bluebelle grant HTA - 12/200/04), led the whole study including chairing of management and executive meetings, supervised development of the questionnaires; **Natalie Blencowe** co-applicant in the Bluebelle study grant, provided feedback on earlier versions of the questionnaires and recruited patients/health care professionals for interviews; **Melanie Calvert** co-applicant in the Bluebelle study grant (patient-reported outcomes expertise); **Joanna Coast** co-applicant in the Bluebelle study grant (Health Economics lead); **Jenny L Donovan** co-applicant in the Bluebelle study grant (Qualitative research co-lead), co-designed and supervised the qualitative research; **Tim Draycott** co-applicant in the Bluebelle study grant (Obstetric surgical expertise and leadership) principal investigator and lead advisor for obstetric surgery; **Jo Dumville** provided feedback on earlier versions of the questionnaires; **Daisy Elliott** wrote the first draft of the manuscript and edited/finalised the paper, contributed to all aspects of qualitative data collection and qualitative data analysis and led development of the questionnaires; **Louise Flintoff** recruited patients/health care professionals for interviews; **Rachel Gooberman-Hill** co-applicant in the Bluebelle study grant (patient and public involvement lead); **Robert Longman** co-applicant in the Bluebelle study grant (Surgical expertise and leadership); **Rhiannon Macefield** conducted literature reviews and provided feedback on earlier versions of the questionnaires; **Laura Magill** co-applicant in the Bluebelle study grant, trial manager in Birmingham; **Jonathan Mathers** co-applicant in the Bluebelle study grant (Qualitative research co-lead); **Christel McMullan** contributed to all aspects of qualitative data collection and qualitative data analysis, provided feedback on earlier versions of the questionnaires; **David Messenger** recruited patients/health care

professionals for interviews; **Charlotte Murkin** conducted pre-testing interviews; **Helen van der Nelson** recruited patients/health care professionals for interviews; **Tom Pinkney** co-applicant in the Bluebelle study grant (Surgical expertise and leadership); **Barnaby C Reeves** co-applicant in the Bluebelle study grant (Methodological lead), contributed to overall study design, and provided feedback on earlier versions of the questionnaires; **Chris A Rogers** co-applicant in the Bluebelle study grant (Statistical lead); **Leila Rooshenas** contributed to all aspects of qualitative data collection and qualitative data analysis in Phase I and provided feedback on earlier versions of the questionnaires; **Andrew Torrance** co-applicant in the Bluebelle study grant; **Nicky J Welton** co-applicant in the Bluebelle study extension grant; **Mark Woodward** co-applicant in the Bluebelle study grant (Paediatric surgical expertise and leadership) and advisor on paediatric surgery; **Trudie Young** co-applicant in the Bluebelle study grant (Wound nursing specialist) and advisor on wound care.

COMPETING INTERESTS

None declared.

DISCLAIMER

The views expressed are those of the authors and not necessarily those of the MRC, NHS, NIHR or the Department of Health.

FUNDING

The Bluebelle study (Phase A) is funded by the National Institute for Health Research (NIHR) Health Technology Assessment (HTA) Programme (HTA - 12/200/04). JLD and JMB are NIHR Senior Investigators. JLD is also supported by the NIHR Collaboration for Leadership in Applied Health Research and Care (CLAHRC) West at University Hospitals Bristol NHS Foundation Trust. The Bluebelle study was undertaken with the support of the MRC ConDuCT-II Hub (Collaboration and innovation for Difficult and Complex randomised controlled Trials In Invasive procedures - MR/K025643/1).

ETHICAL APPROVAL

Ethical approval for this work was granted by the Camden and King’s Cross Research Ethics Committee (14/LR/0640) on the 10th April 2014.

For peer review only

DATA SHARING STATEMENT

No additional data are available.

For peer review only

Table 1: Consolidated criteria for reporting qualitative research (COREQ)

	No	Item	Guide questions/description	Comment
			**Domain 1: Research team and reflexivity**	
**Personal characteristics**	1	Interviewer/facilitator	Which author/s conducted the interview or focus group?	LR, DE, CMM and CM *(see pages 6 and 7)*
	2	Credentials	What were the researcher's credentials? e.g. *PhD, MD*	DE - BSc, PhD<br>LR - BSc, PhD<br>CMM - BA Hons, PgDip, MA PhD<br>CM – MB ChB BSc
	3	Occupation	What was their occupation at the time of the study?	DE - Qualitative Research Associate in Health Services Research<br>LR - Lecturer in Qualitative Health Science<br>CMM - Qualitative Research Fellow<br>CM - Research Fellow
	4	Gender	Was the researcher male or female?	Females
	5	Experience and training	What experience or training did the researcher have?	DE, LR and CMM have several years of experience conducting qualitative research. This has included completing many qualitative projects and attending training courses and workshops.
**Relationship with participants**	6	Relationship established	Was a relationship established prior to study commencement?	No
	7	Participant knowledge of the interviewer	What did the participants know about the researcher? *e.g. personal goals, reasons for doing the research*	The researchers introduced themselves, explained the purpose of the research and provided an information leaflet about the study
	8	Interviewer characteristics	What characteristics were reported about the interviewer/facilitator? *e.g. Bias, assumptions, reasons and interests in the research topic*	The researchers explained how qualitative research related to main Bluebelle trial
			**Domain 2: study design**	
**Theoretical framework**	9	Methodological orientation<br><br>and theory	What methodological orientation was stated to underpin the study? *e.g. grounded theory, discourse analysis, ethnography, phenomenology, content analysis*	Data were analysed thematically using techniques of constant comparison derived from grounded theory methodology *(see page 6)*

For peer review only

**Participant selection**	10	Sampling	How were participants selected? *e.g. purposive, convenience, consecutive, snowball*	Purposeful *(see pages 6 and 7)*
	11	Method of approach	How were participants approached? e.g. *face-to-face, telephone, mail, email*	Patients were approached face to face by healthcare professionals. Healthcare professionals were contacted by the researchers via email. *(See pages 6 and 7)*
	12	Sample size	How many participants were in the study?	Sixty-four patients and 15 health care professionals from abdominal general surgical specialities and obstetrics *(See Table 1 on pages 7/8)*
	13	Non-participation	How many people refused to participate or dropped out? Reasons?	Two patients were unable to take part due to poor health.
**Setting**	14	Setting of data collection	Where was the data collected? *e.g. home, clinic, workplace*	Patient interviews were conducted whilst patients were in hospital. Health professionals chose a location that was convenient for them (their workplace or a nearby café) or opted to do the interview over the telephone. *(See pages 6 and 7)*
	15	Presence of non-participants	Was anyone else present besides the participants and researchers?	The partners of patients sometimes sat with the patients but spoke very little; their comments were not included in the final analysis.
	16	Description of sample	What are the important characteristics of the sample? *e.g. demographic data, date*	Participants' full details are provided in Table 1, and key information is provided in the results section *(See pages 7/8)*
**Data collection**	17	Interview guide	Were questions, prompts, guides provided by the authors? Was it pilot tested?	A topic guide was developed (based on literature and views of health care professionals in the Bluebelle study team) to ensure that discussions covered the same core issues but with sufficient flexibility to allow new issues of importance to the informants to emerge. Although not piloted, it was adapted as analysis progressed to enable exploration of emerging themes. *(Topic guides are included in Additional File)*
	18	Repeat interviews	Were repeat interviews carried out? *If yes, how many?*	No repeat interviews were carried out
	19	Audio/visual recording	Did the research use audio or visual recording to collect the data?	Interviews were audio-recorded *(see page 6)*
	20	Field notes	Were field notes made during and/or after the	The researchers kept notes throughout data

For peer review only - http://bmjopen.bmj.com/site/about/guidelines.xhtml

			interview or focus group?	collection and analysis *(See page 8)*
	21	Duration	What was the duration of the interviews or focus group?	Interviews lasted an average of 25 minutes (range = 15–50 minutes). *(See page 8)*
	22	Data saturation	Was data saturation discussed?	Data collection continued until the team were confident that saturation had been reached *(See page 7)*
	23	Transcripts returned	Were transcripts returned to participants for comment and/or correction?	Transcripts were not returned to participants for comments or corrections
**Domain 3: analysis and findings**				
**Data analysis**	24	Number of data coders	How many data coders coded the data?	All data were coded by DE or CMM. A subset of approximately half of the interviews (n = 19) was double coded by a third researcher (LR). *(See page 7)*
	25	Description of the coding tree	Did authors provide a description of the coding tree?	A list of issues from the analysis of the interviews and literature search was collated into an item tracking matrix. *(See additional File)*
	26	Derivation of themes	Were themes identified in advance or derived from the data?	Issues which were conceptually similar were organised into categories. For instance, issues such as 'itchiness/irritation', 'presence of pulling sensation', and 'tightness of wound' were mapped into a 'wound comfort' category. *(See Table 2)*
	27	Software	What software, if applicable, was used to manage the data?	NVivo (version 10) was used to analyse the data (See page 6)
	28	Participant checking	Did participants provide feedback on the findings?	Full results were not sent out to all participants to gain respondent validation.
**Reporting**	29	Quotations presented	Were participant quotations presented to illustrate the themes / findings? *Was each quotation identified? e.g. participant number*	The interpretation of each category is supported by illustrative quotes *(See Table 2)*
	30	Data and findings consistent	Was there consistency between the data presented and the findings?	There is consistency between the data presented and the measures developed. The item tracking matrix provides an overview of the key findings and how these were used to develop the initial measure *(See additional file)*
	31	Clarity of major themes	Were major themes clearly presented in the findings?	The themes are clearly presented in the

For peer review only - http://bmjopen.bmj.com/site/about/guidelines.xhtml

				findings *(See pages 8-11)*
	32	Clarity of minor themes	Is there a description of diverse cases or discussion of minor themes?	Yes. Differences between the findings of the interviews and the literature search are discussed, as are the differences in satisfaction between the dressing types *(See pages 9 and 10)*

For peer review only

For peer review only - http://bmjopen.bmj.com/site/about/guidelines.xhtml

Category	Issue	Identified by				Additional comments	Included in questionnaire?	
		Interviews			Literature (n=26 RCTs)		If yes, questionnaire item	Why not included
		DE (n=28)	LR (n=19)	CM (n=11)				
Wound comfort	Itchiness	✓	✓	✓	✓	CM: Described by patients as an outcome (looking at the wound to check if it is irritated, inflamed, etc)<br>DE: Similar to inflammation ('burning'	Q1: Has the wound been itchy?	
	Pain	✓	✓	✓	✓	DE: Described as 'sore', 'hurt', 'tender', 'uncomfortable'.<br>DE: Sometimes mentioned in the context of a numerical scale.<br>RM: Described as 'burning pain referring to a dressing-related sensation felt under the dressing' in one study<br>RM: Also 'tenderness'<br>CM: 'sore', 'painful' – discussed in terms of dressing removal	Q2: Has the wound been painful?	
	Presence of pulling sensation	✓			✓	LR: Described as wound 'being able to breathe', 'stuffiness'	Q3: Has the wound had a pulling sensation?	
	Tightness of wound	✓	✓		✓		Q4: Has the wound felt tight?	
	Wound comfort (overall or unspecified)				✓	RM: Also measured as 'Discomfort' in the literature<br>RM: Includes 'Discomfort with skin problems'		Excluded as covered in Q1 – Q4
Exudate and its impact	Whether there was any exudate	✓	✓	✓	✓		Q5: Has the wound leaked?	
	Type of exudate (blood, other)	✓	✓	✓	✓	DE: Described as 'mess', 'manky', 'leaking', 'gunge', 'oozing', 'soaking', 'brown mess'<br>LR: 'moistness', Ooziness', 'dampness'<br>RM: Described as 'discharge' 'fluid' 'oozing'<br>CM: 'Seeping'	Q5: If so, was it: clear fluid? cloudy fluid? Blood-stained fluid? thick and yellow/green fluid?	
	Whether exudate marks bedding/clothing	✓	✓	✓		DE: Described as 'stains', LR: 'manked up clothing'	Q6: Has the leakage resulted in changed bedding/ clothes?	
	Degree dressing absorbs exudate	✓	✓	✓	✓		Q7: How would you describe the wettest dressing?	
	Whether additional dressing required	✓	✓	✓	✓	RM: Includes reasons for dressing changes<br>CM: use two dressings when oozing is important, don't want to take original dressing off	Q8: Has a dressing or glue been put on the wound (or replaced)?	
	Anxiety associated with exudate	✓	✓	✓				Excluded as captured in Q15: "Have you felt any anxiety about your wound?"
Allergic reactions to the dressing	Any allergic reactions to dressing/blistering	✓	✓	✓	✓	RM: Also include skin damage/injury	b) has the wound blistered?	
Dressing removal	Whether dressing comes off	✓	✓	✓			Q9: Has the dressing or glue come off or been removed	
	Whether dressing needs to be taken off (if so, by patient or	✓	✓	✓		CM: patient or partner	Q9 Q9: Has the dressing or glue come off or been removed?<br>If "Yes", was it taken off by a	

For peer review only

	professional)						doctor/nurse/other health specialist?	
	Whether travel is required to change/remove dressing (i.e. seeing nurse or GP/post op visits)	✓	✓					Not relevant to early post-operative period
	Any discomfort during removal	✓	✓	✓	✓		Q10 Was there any discomfort when removing the dressing?	
	Any pain during removal	✓	✓	✓		DE: Causes 'pain', skin is 'tender', 'sore', pulls hairs, sticks to skin)<br>RM: 'Pain on removal of the dressing'	Q11 Was there any pain when removing the dressing?	
**Wound protection**	Dressings protecting the wound				✓		Q12 Has the wound felt protected? (i.e. from catching on anything or being knocked)	
	Whether dressing/wound rubs on clothes	✓	✓	✓		LR: Awkwardness of wearing clothes over dressing		
	Whether dressing/wound catches on other things	✓	✓	✓		CM: bedsheets		
**Impact on daily activities**	Ability to get back to work	✓	✓				Q13: Have you been able to perform everyday tasks? (i.e. showering/ bathing, getting dressed)	
	Ability to shower/bathe	✓	✓	✓	✓	RM: Described as 'Ability to facilitate personal hygiene' in one study<br>RM: Described as 'Appreciation of possibility to shower' in one study. Also 'satisfaction with the possibility to wash oneself'		
	Ease of movement (e.g sitting, walking, stairs)	✓	✓		✓	DE: Standing up, walking<br>RM: Described as 'Ability to facilitate mobility' in the literature. Also 'Does the dressing limit you in movement?'<br>DE and LR: Includes sneezing/coughing		
	Ability to perform everyday tasks (e.g self-care)	✓	✓	✓		DE: Washing/self-care, driving, walking, housework, cooking, exercise		
	Ease of getting dressed	✓						
	Going to the toilet		✓	✓				
	Self-management of wound		✓	✓	✓	RM: 'Ease of managing wound' was a PRO measured in the first 3 weeks after surgery on a 1-10 scale in one study		
	Change to usual clothing		✓	✓				
	Overall recovery				✓	RM: not a PRO (surgeon rating)		
**Ease of movement**	(Dis)comfort when sitting	✓	✓	✓			Q14: Have you been able to move around easily?	
	(Dis)comfort when lying	✓	✓					
	(Dis)comfort whilst	✓	✓					

For peer review only

	sleeping/sleep quality							
	(Dis)comfort whilst moving	✓	✓	✓		CM: related to tightness of dressing		
**Anxiety about the wound**	Feeling of security/safeness (in relation to the wound?)	✓	✓	✓			Q15: Have you felt any anxiety about your wound?	
	Feeling of vulnerability		✓	✓				
	Not having to worry about wound/dressing	✓	✓	✓		DE: 'You can just forget about it, you don't have to think about		
	Feeling protected	✓	✓	✓				
	Stress levels/psychological discomfort/anxiety	✓	✓					
	Feeling constricted by dressing	✓	✓					
	Cleanliness of environment	✓	✓	✓		DE: Described as 'hygiene', 'coming into contact with bugs'		
	Fear of infection	✓	✓	✓				
	Anxiety about bodily contents spilling out/wound bursting open	✓	✓	✓		LR: Described as 'coming open', 'split apart', 'rupture'		
**Satisfaction with dressing/ not having a dressing**	Satisfaction with (appearance of) dressing	✓	✓		✓	LR: 'Neatness', 'prominence'<br>RM: Measured as 'How satisfied overall do you feel with your dressing?'	Q16: Have you felt satisfied with having/not having a dressing?	
	Whether patient would prefer to see the wound	✓	✓			DE: Anxiety/reassurance/fear/discomfort<br>RM: Described as 'how well the incision could be seen under the dressing' and 'transparency'		
	Degree dressing fits contours of the skin/clothing	✓	✓		✓	RM: Described as 'Conformability of the dressing to the wound' in one study		
	Dignity			✓		CM: Having a dressing gives more dignity		
	Confidence			✓		CM: Confidence to walk around without having to worry		
	Appreciation of absence of bandage				✓			
	How long dressing stays on	✓	✓	✓	✓	CM: 'the longer you leave a dressing on the harder it is to get off'		
	Ease of removal	✓	✓	✓	✓	RM: 'Ease of dressing application' was measured in one study but this was rated by a surgeon not patient<br>LR: Any exudate from removal<br>DE, CM,: Any remnants remaining after removal ('bits of glue')		
	Degree of stickiness	✓	✓	✓	✓	CM: stitches stick to legs when sitting (gynae)<br>DE: Mostly considered to be positive, but sometimes makes removal tricky		

For peer review only

						DE: Described as: 'It stays in place and doesn't wriggle up'<br>RM: Described as 'Dressing integrity' in one study also 'How much the dressing had loosened'<br>CM: dressings don't stick well because of body hair		
	Awareness of dressing/wound	✓	✓	✓	✓			
	If patient reapplies, ease of reapplying dressing	✓	✓		✓			
	Whether additional support or materials provided	✓	✓	✓				
	Whether patient uses own dressing	✓	✓	✓				
	If patient reapplies, ease of reapplying dressing	✓	✓		✓			
	Overall satisfaction/satisfaction with overall experience				✓			
**Wound appearance**	Perceptions of healing	✓	✓	✓	✓	DE: Described as 'healing very nicely', 'rate of healing'<br>LR: Sub-codes – 'quality of healing', 'speed of healing', 'whether healed or not', 'suggested as main outcome'. Patients often discuss scab formation when talking about healing.<br>RM: Measured as 'Effectiveness (wound healing)' in one study 1= well healed, 3=poorly healed, not a PRO. Also measured as a PRO in another study 'Has your wound healed?'<br>CM: Healing could be a standalone category (one of the outcomes): definition of healing, healing time		Relevant to longer term outcomes of wound healing (not relevant within first days of surgery)<br>Patients' wounds not visible if dressing applied
	Bruising	✓	✓			DE: Described as 'black and blue'		
	Colour	✓	✓	✓	✓	DE: Described as 'purple'/'pink'/'grey'/'red' (see inflammation')<br>LR: 'Black and bluish' (also fit under 'bruising', as above)<br>RM: Redness<br>CM: Colour was talked about more in the context of healing, ie as an indicator (for the patients) of whether or not the wound was healing		
	Cosmesis/aesthetics		✓		✓	RM: Described as 'cosmesis', 'cosmetic outcome' and 'cosmetic result' in the literature		
	Scars	✓	✓	✓	✓	DE: A long term measure<br>RM: Pigmentation, scar colour, prescence of		

For peer review only

						inflammation, suppleness or pliability, scar height or evenness with the surrounding skin, using modified Vancouver Burn Assessment Scale		
	Size	✓	✓	✓				
	Scabbing	✓	✓	✓		DE: Associated with healing		
	Inflammation/swelling	✓	✓	✓		DE: Described as ‘burning’<br>CM: Described by patients as an outcome		
	Overall appearance of wound	✓	✓	✓		DE: Described as ‘unsightly’, ‘neat’, ‘tidy’, ‘messy’ LR: ‘ugly’<br>CM: ‘Smooth’		
	Maceration of the skin				✓	RM: not a PRO		
	Satisfaction with the appearance of the wound				✓			
	Whether patient would prefer to see the wound	✓	✓					

For peer review only

Interview Topic Guide: Phase 1: Generation of relevant issues

Background, interviewee details and ice breaker

- Interviewee background and details of procedure that the interviewee has had (type of procedure elective or emergency? have you had before?, when, where, length of stay in hospital, recovery period).
  - *Just to give me some background, can you just tell me a little bit about the procedure that you had, the surgical procedure?*
  - *How have you been, in terms of recovery? Length of stay? When came home?*
  - *Is this your first?*

Expectations/experiences of wound care

- *Did/do you have any expectations about whether or not you would have a dressing? Where do you think these expectations came from?*
- *What kind of dressing? When did they take it off? [home/hospital?] Same one? How did you find having the dressing? Why do you think you had/did not have a dressing? Do you think it had any impact on your wound healing?*
- *Why do you think they do/don't use dressings?*
- *Thoughts/reactions to **alternative** wound management methods (i.e. dressing or no dressing in relation to what patient has experienced/what patient expects). (Explore patient thoughts on impact this may have on previously mentioned issues (e.g. recovery, symptoms, practicalities).*

Patient perspectives on a trial of dressing type

Okay, the basis of this study is like I said, we're trying to find out if dressing the wounds is helpful or not. In a study, patients would be randomly allocated by chance to either receive dressings or receive no dressings. The doctor wouldn't decide and the patient wouldn't decide. What we're trying to find out is, in patients who have had a similar type of surgery, if we have one group of patients that have them on so and another group of patients that don't, then can we compare them to see if there's any difference between the groups.

- *Do you think you would participate? (Reasons why/why not? Any reservations?)*
- *Can you imagine your family and/or friends would participate in a study like this?*
- *What kinds of things do you think you might consider, when deciding whether or not to take part? Do you think you would have any questions about the study? What kind of things might you want to know about in advance?*
- *How do you think you would feel about random allocation to dressing type, specifically the possibility of receiving no wound dressing? [explain randomisation to patients, then ask…] If you were in a group that didn't receive a dressing, do you think you would have thought about your care differently?*
- *In paediatrics, they don't use dressings. Changes impression?*
- *What about a glue dressing?*
- *Can you think of any potential problems we might come across if we were doing this study, any practical problems or any difficulties we might come across?*
- *Perspectives on important outcomes to include in a trial of dressing use. We would like this study to help us answer the question of whether dressings should be used in patients such as yourself (and if so, what type of dressing is best). What do you think are the important factors we should consider when making any future recommendations on dressing use? Satisfaction? Infection? Healing times?*

Closing

- Summarise key points
- Any further questions?
- Thank patient for their time and explain how they have helped.

For peer review only

Interview Topic Guide: Phase 3 – Pre-testing

Introduction

- As you know, we are going to audio-record you whilst you complete the questionnaire that we have developed. During this, we would like to understand your opinion of the questions, in terms of the language used and the relevance to your post-operative experience. It is important to remember that there are no right or wrong answers, but that this will help us to understand how the questionnaire should be developed. This should take no longer than an hour, although you can stop the discussion at any time. The information that you provide is not going to be fed back to your clinical team.
- Complete consent form and data collection form
- Any questions?

Background (procedure and recovery)

- Could you tell me a little bit about what you had done? *(Probe: When? Where? Elective/emergency?)*
- How has recovery been for you so far? *(Probe: How feeling? Length of stay? When expected to go home?)*
- How has the wound been healing? *(Probe: How does wound feel? Any problems?)*

PWMQ: Can we work through the questionnaire as you complete it, with you explaining how you understand the items and what you will put for this question.

- DT to not clarify meaning, but just repeat question.
- If P uncertain, try to understand why.
- Note any potential issues to be discussed after completion.

PWMQ: Understanding responses to each question

- Can you tell me in your own words what that question was asking? *(Probe: What does the word XX mean to you? Are there any other ways you would describe it?)*
- What does [not at all/a little/quite a bit/very much] mean to you?
- Was it easy to choose an answer?
- Explore where participants have indicated confusion

After completion:

- What did you think of the questionnaire?
- Were the questions relevant to your experience? *(Probe: Are there any others that should be included?)*
- Any difficulties filling in questions?
- Do you think it captures your experience of your wound in the past 24 hours?
- What does the word "practical wound management" mean to you?
- Do you have any other suggestions on how the questionnaire could be improved? *(Probe: alternative wording, additional questions, response categories, layout/presentation, length of questionnaire?)*
- You have given your answers based on the past 24 hours. Would your answers differ if you were thinking about your entire recovery from surgery? *(Probe: When do you think would be the best time to complete questionnaires like these?)*

Closing

- Summarise key points
- Any further questions?
- Thank patient for their time and explain how they have helped.

For peer review only

Developing outcome measures assessing wound management and patient experience: A mixed methods study

Journal:	*BMJ Open*
Manuscript ID	bmjopen-2017-016155.R1
Article Type:	Research
Date Submitted by the Author:	11-May-2017
Complete List of Authors:	Elliott, Daisy; University of Bristol, School of Social and Community Medicine<br>Andronis, Lazaros; The University of Birmingham, Health Economics Unit, Institute of Applied Health Research<br>Blazeby, Jane; University of Bristol, Department of Social Medicine<br>Blencowe, Natalie; University of Bristol, Centre for Surgical Research, School of Social and Community Medicine<br>Calvert, Melanie; The University of Birmingham, Primary Care and General Practice<br>Coast, Joanna; University of Bristol, School of Social and Community Medicine<br>Draycott, Tim; Obstetrics & Gynaecology, North Bristol NHS Trust<br>Donovan, Jenny; University of Bristol, School of Social and Community Medicine<br>Gooberman-Hill, Rachael; University of Bristol, School of Clinical Sciences<br>Longman, Robert; University Hospitals Bristol, Dept of Coloproctology<br>Magill, Laura ; Queen Elizabeth Hospital<br>Mathers, Jonathan; University of Birmingham, Public Health<br>Pinkney, Thomas ; University of Birmingham, Centre for Patient Reported Outcomes Research; Queen Elizabeth Hospital, Academic Department of Surgery<br>Reeves, Barnaby; University of Bristol, Bristol Heart Institute<br>rogers, chris; University of Bristol, Bristol Heart Institute<br>Rooshenas, Leila; University of Bristol, School of Social and Community Medicine<br>Torrance, Andrew; The University of Birmingham, Institute of Applied Health Research<br>Welton, Nicky; University of Bristol, School of Social and Community Medicine<br>Woodward, Mark; University Hospitals Bristol<br>Young, Trudie; Welsh Wound Innovation Centre<br>Dumville, Jo C.; University of Manchester, School of Nursing, Midwifery & Social Work<br>Flintoff, Louise; University Hospitals Bristol<br>Macefield, Rhiannon; University of Bristol, Department of Social Medicine<br>Messenger, David; University Hospitals Bristol<br>McMullan, Christel; University of Birmingham, Institute of Applied Health Research<br>Murkin, Charlotte; University of Bristol, School of Social and Community Medicine

	van der Nelson, Helen; North Bristol NHS Trust, Obstetrics & Gynaecology

**Primary Subject Heading**:	Surgery
Secondary Subject Heading:	Qualitative research
Keywords:	QUALITATIVE RESEARCH, Wounds, Dressings, Outcome measures, Patient experience, Questionnaire development

SCHOLARONE™ Manuscripts

For peer review only

Developing outcome measures assessing wound management and patient experience: A mixed methods study

The Bluebelle Study Group*

Corresponding author: Daisy Elliott [daisy.elliott@bristol.ac.uk]

*The Bluebelle Study Group consists of the following sub-groups:

Bluebelle grant co-applicants: Lazaros Andronis[1], Jane Blazeby[2,3], Natalie Blencowe[2,3,] Melanie Calvert[4], Joanna Coast[2,7], Tim Draycott[6], Jenny L Donovan[2,7], Rachael Gooberman-Hill[8], Robert Longman[3], Laura Magill[9], Jonathan Mathers[4], Thomas Pinkney[5], [9], Barnaby C Reeves[10], Chris A Rogers[10], Leila Rooshenas[2,] Andrew Torrance[4], Nicky J Welton[2], Mark Woodward[3], Trudie Young[11]

Other members of the Bluebelle Study Group: Jo Dumville[12], Daisy Elliott[2], Louise Flintoff[3], Rhiannon Macefield[2], David Messenger[3], Christel McMullan[4], Charlotte Murkin[2,3], Helen van der Nelson[6]

1. Health Economics Unit, Institute of Applied Health Research, University of Birmingham, Birmingham, UK.

2. School of Social and Community Medicine, University of Bristol, Bristol, UK.

3. University Hospitals Bristol NHS Foundation Trust, Bristol, UK.

4. Institute of Applied Health Research, University of Birmingham, Birmingham, UK.

5. Centre for Patient Reported Outcomes Research, University of Birmingham

6. North Bristol NHS Trust, Bristol, UK.

7. NIHR Collaboration for Leadership in Applied Health Research and Care West at University Hospitals Bristol NHS Trust, Bristol, UK

8. Musculoskeletal Research Unit, School of Clinical Sciences, University of Bristol, UK.

9. Academic Department of Surgery, Queen Elizabeth Hospital, University of Birmingham, UK.

For peer review only

10. Clinical Trials and Evaluation Unit, School of Clinical Sciences, University of Bristol, Bristol, UK.

11. Welsh Wound Innovation Centre, Rhodfa Marics, Ynysmaerdy, Pontyclun, Rhondda Cynon Taf, Wales, UK.

12. School of Nursing, Midwifery & Social Work, University of Manchester, Manchester, UK.

Running title: Outcome measures for wound management and patient experience

Keywords: Qualitative research; wounds; dressings; outcome measures; instrument development; patient-reported outcomes; questionnaire development; patient experience

Word count: 2735 (excluding title page, abstract, tables and references)

For peer review only

ABSTRACT

Objectives: To develop outcome measures to assess practical management of primary surgical wounds and patient experience.

Design: Mixed methods, including qualitative interviews and data extraction from published RCTs.

Setting: Two university-teaching NHS hospitals and three district NHS hospitals in the South West and Midlands regions of England.

Participants: Sixty-four patients and 15 health care professionals from abdominal general surgical specialities and obstetrics (caesarean section).

Methods: Measures were developed according to standard guidelines to identify issues relevant to patients' experiences of surgical wounds and dressings, including analysis of existing RCT outcomes and interviews. These were written into provisional questionnaire items for a single outcome measure. Cognitive interviews with patients and health care professionals assessed face validity, acceptability and relevance. Findings from interviews were regularly shared with the study team who suggested amendments to modify and reword items to improve understanding before further iterative testing with patients and health care professionals.

Results: Analyses of existing RCT outcomes and interviews produced a total of 69 issues. Pre-testing and iterative revision established the need for two separate measures. One measure addresses health care professionals' experience of wound management in two key areas: exudate and its impact, and allergic reactions to the dressing. The other measure addresses patients' experience of wounds in seven key areas: wound comfort, dressing removal, dressings to protect the wound, impact on daily activities, ease of movement, anxiety about the wound and satisfaction with dressing. Each measure took less than five minutes to complete and were understood and acceptable to patients and health care professionals.

Conclusion: This in-depth study has developed two measures to assess practical management of primary surgical wounds and patient experience. Further work to test their validity and reliability and application to other settings is now required.

STRENGTHS AND LIMITATIONS OF THIS STUDY

- This is the first study to explore the important issues related to the practical management of primary surgical wounds and patient experience immediately following surgery.

For peer review only

- This study used robust methods to identify key issues of outcome that could be used to inform decision-making around dressings. Interviews provided a rich account of the key factors that affected wound management and patient experience while a purposeful sampling strategy ensured that perspectives were captured from a range of participants. Data produced from the interviews were supplemented by analysis of existing RCT outcomes to ensure a comprehensive list of issues was considered.
- Future work is needed to test the reliability, validity and sensitivity of the new measures.

For peer review only

INTRODUCTION

An estimated 234 million major surgical procedures are undertaken worldwide every year (1). It is common practice to apply dressings over the closed wound in adult surgery and many different dressing types are available (2). A recent Cochrane systematic review summarised data relating to wound dressings and risk of surgical site infection (SSI) in primary surgical wounds. No evidence was found to suggest that any type of dressing significantly reduced the risk of developing an SSI compared with leaving wounds exposed; neither was there any benefit associated with particular dressings (2).

Decision-making around dressings may therefore need to be informed by other properties and qualities that dressings can offer, such as absorption of exudate, patient comfort, offering physical protection, facilitating wound observation, and meeting patients' desires for wound coverage (3). Whilst measures for assessment of wound cosmesis (in the longer term) are available (4, 5), there is a lack of well-developed and validated measurement tools relating to practical wound management or patient experience (2, 3, 6). Such an instrument could be used to monitor the care of individual patients (e.g. assessing the ability of dressings to manage specific symptoms), audits (e.g. quality assurance) and research (e.g. comparing patient satisfaction).

The development of patient-reported outcome measures (PROMs) increasingly includes the use of qualitative research methods that provide the opportunity to elicit and characterise patients' experiences of their health conditions and treatment (7, 8). Qualitative methods can also define health professionals' experiences of care and management (9). Data can be supplemented by expert input and studies in published literature (10, 11). This article describes the development of measures to assess practical wound management issues, symptoms and patient experience associated with primary surgical wounds.

METHODS

Study design

Measures were developed according an existing framework for developing PROMs (12, 13), also incorporating guidance on eliciting health domain concepts using qualitative methodologies (10, 11, 14). The study is reported according to qualitative reporting guidelines (See Supplementary File 1). Phase 1 aimed to produce a comprehensive list of potential issues relating to wound and dressing experience and practical management issues. Phase 2 developed issues identified from Phase 1 into questionnaire items. Phase 3

For peer review only

evaluated the measures for acceptability and relevance using cognitive interviews with patients and health care professionals. The final part of development (phase 4) consisted of psychometric testing and will be reported elsewhere. Ethical approval was provided by the NHS Health Research Authority NRES Committee London – Camden & Kings Cross (14/LO/0640). Written informed consent was provided by all participants.

Phase 1: Generation of relevant issues

1.1 Interviews

Interviews were conducted with patients to explore and characterise experiences of wounds and dressings. Participants were recruited as part of a wider feasibility study to explore whether a trial comparing different types of dressings, and dressing versus no dressings, is possible (The Bluebelle study: a feasibility study of three wound dressing strategies in elective and unplanned surgery, HTA - 12/200/04 (15, 16)). Participants were recruited from two University-teaching NHS hospitals and three district NHS hospitals in the South West and Midlands regions of England. Eligible patients had undergone, or were scheduled to undergo, an abdominal surgical procedure or caesarean section were identified and approached by research nurses and surgical trainees. The qualitative team contacted interested patients to arrange interviews. A purposeful sampling strategy ensured that perspectives were captured from a range of participants (11). Within this sampling approach, maximum variation was sought in relation to age, gender, ethnicity, type of surgery, dressing type and location. A topic guide was developed (based on literature and views of health care professionals in the Bluebelle study team) to ensure that discussions covered the same core issues but with sufficient flexibility to allow new issues of importance to the informants to emerge (See Supplementary File 2).

Interviews were audio-recorded and transcribed in full. Transcripts were imported into NVivo (version 10). All data relating to outcomes and issues of importance to patients that were relevant to dressing use and the practical management of the wound in the initial time period post-surgery were assigned labels (coded) by two experienced qualitative researchers. Data were analysed using techniques of constant comparison derived from grounded theory methodology, and emerging codes across the dataset were then compared to look for shared or disparate views among participants (17). A subset of approximately half of the interviews (n = 19) was double coded by third experienced researcher to highlight any differences in the interpretation of codes (10). Data collection and analysis continued until the team were

For peer review only

confident that saturation had been reached i.e. no more patterns or themes emerged from the data (18).

1.2 Extraction of information from three systematic reviews

Three systematic reviews were used to identify RCTs which included outcomes relating to patient experience and management of wound healing. Published papers reporting the studies were obtained where possible. Relevant data were extracted on outcome (as described by the authors), verbatim wording to measure outcome, who reported the outcome, measurement scale and assessment time point. Attempts were made to contact the authors for more information.

1.3 Synthesis of findings from interviews and data extraction

A list of issues from the analysis of the interviews and analysis of existing RCT outcomes was collated into an item tracking matrix, in line with guidance for developing PROMs (19). This is available in the online appendix (see Supplementary File 3). The study team agreed on a set of words or phrases to reflect each issue and also noted additional phrasing made by participants in a subsequent column (10). Issues which were conceptually similar were organised into categories. For instance, issues such as 'itchiness/irritation', 'presence of pulling sensation', and 'tightness of wound' were mapped into a 'wound comfort' category.

Phase 2: 'Operationalisation': Construction of a provisional measure

The item tracking matrix was used to determine which issues should be written into questionnaire items. Items featured words and phrases used by patients in the interviews to enhance content validity (11, 20).

Phase 3: Pre-testing

Participants were recruited from two University-teaching NHS hospitals in the South West and Midlands regions of England. Patients who had undergone abdominal general surgery or caesarean section and health care professionals involved in post-surgical care were approached. As in Phase 1, sampling was purposeful to achieve maximum variation in relation to clinical role, age, gender and geographic location (for health care professionals) and age, gender, ethnicity, type of surgery, dressing type and location (for patients).

Cognitive interviews are used widely in questionnaire development (10) and involves asking respondents to verbalise their thoughts while answering questions (21). This methodology

For peer review only

enabled us to explore the acceptability of the measure and coverage of patients and health care professionals' concerns (in terms of language, accuracy, and relevance) as well as layout (11). During each interview, participants were asked to complete the measure by reading each item aloud and commenting on their understanding. Interviews were guided by a series of probes (e.g. 'What does this item mean to you?', 'Are there other ways you would describe it?'; (21)). Participants' body language was also observed and prompted further discussion about specific items (such as the participant nodding in agreement or frowning) (10). A copy of the topic guide is available (See Supplementary File 2).

The qualitative team maintained detailed field notes from each interview, describing suggestions for modifications and improvements to the provisional measures. Operationalisation and modification of the measures was an iterative process. Findings from the interviews and suggestions for amendments were regularly disseminated to the Bluebelle Study Group, which consisted of a multidisciplinary group of health care professionals, including surgeons, health services researchers and research nurses. Each stage of feedback informed amendments to modify and reword items to improve understanding, which was repeated following efforts to revise questions and eliminate problems (21). This process continued until no new issues were identified and no further refinements were believed to be necessary.

RESULTS

Phase 1: Generation of relevant issues

1.1 Interviews

A total of 39 interviews were conducted between July 2014 and July 2015. Interviews were conducted in person (n=10), unless patients preferred to be interviewed via telephone (n=29). Interviews lasted an average of 25 minutes (range = 15–50 minutes). The sample consisted of 24 women and 15 men, who mostly described themselves as white British (90%). They had a mean age of 56 years (range 41-88 years). Thirty-seven of the 39 participants had either undergone abdominal general surgery (74%) or a caesarean section (26%), with an average of 18 days since their surgery (range =6-40 days). Two of the 39 patients were scheduled to undergo abdominal general surgery and discussed issues that they anticipated would be important to them. Participant demographics for Phase 1 interviews are shown in Table 1.

For peer review only

Table 1: Demographics of participants' interviews in Phases 1 and 3

		Phase 1: Generation of relevant issues	Phase 3: Pre-testing	
**Qualitative interviews (n = 79)**		39 patients	25 patients	15 health care professionals
**Age (years)**	Range	22-88	19-76	23-60
	Mean	56	54	41
**Sex**	Female	27	12	13
	Male	12	13	2
**Ethnicity**	White	35	22	14
	Asian	1	1	0
	African	2	1	1
	Indian	1	0	0
	Filipino	0	1	0
**Type of surgery**	Abdominal	32	25	15
	Obstetric	6	0	0
**Dressing type**	Tissue adhesive	7	5	-
	Adhesive	32	18	-
	No dressing	0	2	-
**Location**	Bristol	28	15	9
	Birmingham	11	10	6

1.2 Extraction of information from three systematic reviews

Published papers for twenty six studies that included outcomes relating to patient experience and management of wound healing were identified from the three systematic reviews (22-47). Only two studies included a validated instrument, or modification of a validated instrument, to assess outcomes (24, 38). These were for long term scarring and cosmesis (4, 5). However, no studies reported using validated measures relating to issues associated with practical wound management and patient experiences in the early post-operative period. Descriptions of outcomes were heterogeneous and often poorly defined. The most common reported outcomes related broadly to cosmetic result (reported in 15/26 studies), dressing changes (e.g. frequency, comfort, ease of application and removal; reported in 11/26 studies), and skin reactions (e.g. itching, blistering; reported in 10/26 studies). Full data extraction from the 26 studies is included in Supplementary File 4.

1.3 Synthesis of findings from interviews and data extraction

When describing experiences in the interviews, patients commented on several factors that affected perceptions of how well their wound was healing, including how it felt (tightness, pain, and itchiness) and whether any fluid had leaked from the wound. Analysis of existing RCT outcomes showed these issues had been captured in some previous (unvalidated) outcomes.

For peer review only

All patients had at least one dressing applied after surgery, although this varied between adhesive coverings (absorptive or non-absorptive) and tissue adhesive as-a-dressing ('tissue glue'). Both the interviews and the analysis of existing RCT outcomes highlighted that multiple practical advantages of dressing use (including ability to contain exudate and ease of removal). The interviews also demonstrated that there were psychological factors that affected dressing experience and satisfaction (i.e. anxiety about cleanliness of the wound). Patients with tissue glue as a dressing commented that they had been surprised that their wounds had been dressed this way (rather than adhesive dressings which they had had in the past for other wounds). However, these patients stated that compared to past experiences of adhesive dressings, they liked how glue was transparent, waterproof, did not require multiple applications and came off naturally.

The interviews and the analysis of existing RCT outcomes produced a total of 69 issues. These were grouped into ten broad categories: wound comfort, exudate and its impact, allergic reactions to the dressing, dressing removal, dressings to protect the wound, impact on daily activities, ease of movement, anxiety about the wound, satisfaction with dressing and wound appearance. Table 2 provides illustrative quotes for the categories identified.

Table 2: Categories identified

Category	Example quote
**Wound comfort**	*"I've now got really itchy where the plaster goes. Which is uncomfortable."* (Patient, adhesive dressing)
**Exudate and its impact**	*"If I walked around it would get really damp. I mean it would soak my pyjamas and drip down my legs. It was quite manky really…Then they would put a sort of big, well, like a big plaster on top of that, and then they put a kind of absorbent pad over that, to absorb some of that liquid."* (Patient, adhesive dressing)
**Reactions to the dressing**	*"I was allergic to the surgical tape."* (Patient, adhesive dressing)
**Dressing removal**	*"I just completely soaked it [adhesive dressing] in the shower then my husband just took it off for me. But it was, it was really easy. Much easier than I thought."* (Patient, adhesive dressing)
**Wound protection**	*"I'd be worried about catching it [the wound], knocking it, or something getting in so that it became infected."* (Patient, adhesive dressing)
**Impact on daily activities**	*"With the glue [dressing] it's easy to shower. With a [adhesive] dressing it wouldn't be so easy to shower and you'd be worried."* (Patient, tissue adhesive dressing)
**Ease of movement**	*"What I do find is the dressings are a bit constricting, especially as I get a bit better because they don't turn with your body so easily and then I feel that it makes me feel more constricted."* (Patient, tissue adhesive dressing)
**Anxiety about the wound**	*"You could catch things just from the air. That made me think, "Well, you'd need something to kind of protect it."* (Patient, adhesive dressing)
**Satisfaction with dressing**	*"Glue [as a dressing] requires no maintenance. I was very pleased. You don't have to change it you just leave it alone … I think that helps with the healing process physically and mentally."* (Patient, tissue adhesive dressing)
**Wound appearance**	*"If it was red and inflamed I would have thought, "Something has gone wrong with it."* (Patient, adhesive dressing)

For peer review only

Phase 2: 'Operationalisation': Construction of a provisional measure

A provisional measure was designed based on the findings from Phase 1. Nine key categories were included: wound comfort, exudate and its impact, allergic reactions to the dressing, dressing removal, dressings to protect the wound, impact on daily activities, ease of movement, anxiety about the wound and satisfaction with dressing. Issues relating to the appearance of the wound were not included as they were only relevant to longer term outcomes of wound healing (not within first days of surgery). Additionally, since most patients reported having an adhesive dressing, many had not seen their wound within this timeframe. The first version of the measure included 16 items, and was provisionally called the Practical Wound Management Questionnaire.

Phase 3: Pre-testing

Cognitive interviews (n = 40) were conducted between July 2015 and March 2016. All interviews were conducted face-to-face. This consisted of 25 patients who were in hospital and had undergone abdominal general surgery, and 15 health care professionals involved in surgical wound care. Demographics are shown in Table 1.

Interviews highlighted issues with content in the initial measure. For example, items regarding the colour of the wound exudate were removed. Questions were rephrased to focus on the experience of having a dressing rather than general recovery after surgery (i.e. '*Have you been able to perform everyday tasks? (i.e. showering/bathing)*' was changed to '*Has your dressing prevented you from showering/washing?*'). Additionally, since four patients commented that the smell of their wound was missing on the measure, an item was added to capture this.

The measure had intended to be administered two days after surgery, although feedback suggested that this needed to be completed up to day four as the patient may be disorientated from surgery in the first few days. However, since there were clear differences in recovery with caesarean section and abdominal surgery patients, a timeframe of within four days of surgery was set, and the measures recorded the date of surgery and date completed to determine context of responses.

Feedback from patients suggested it was difficult to respond to questions about exudate, since a health care professional cared for their wound whilst they were in hospital. If their dressing

For peer review only

had been changed, they were also uncertain about the reason why (i.e. simply as part of standard practice or for other reasons). Therefore, the study team decided to separate the measure into two separate measures. The first related to the practical aspects of wound management and the second related to the patient's experience of the wound/dressing and the psychological aspects (anxiety, satisfaction etc). The two measures were named the Wound Management Questionnaire and the Wound Experience Questionnaires.

Seven versions of measures were modified throughout the pre-testing phase. Pre-testing continued until no new issues were identified and no further refinements were believed to be necessary. The final version of The Wound Management Questionnaire contains four items, whilst The Wound Experience Questionnaire contains 10 items (See Supplementary File 5). Overall, the final versions of the measures were well received. In addition, 96% of participants stated that each measure took less than five minutes to complete.

DISCUSSION

This paper describes the development of two measures for assessing wound management and experience. The Wound Management Questionnaire assesses practical issues early after surgery for completion by health care professionals and The Wound Experience Questionnaire assesses symptoms and patient satisfaction with their wound and dressings. These measures were developed using a mixed methods approach, including data extraction from 26 published RCTs and interviews with 64 patients and 15 health care professionals. Final versions of the measures were easily completed and acceptable to patients and health care professionals. Further work is needed to examine their reliability and validity in a wider group of patients.

Given the absence of evidence supporting the effectiveness of dressings for the prevention of SSIs, decision-making around dressings needs to be informed by issues such as managing wound exudate, offering physical protection and meeting patients' desires for wound coverage. However, systematic reviews have highlighted a lack of meaningful outcome data on wound symptom management and patient experiences of primary surgical wounds and acceptability of dressings (2, 3, 6). To our knowledge, this is the first study to explore these important issues in patients with closed primary surgical wounds. The measures are intended to be used in future studies including a wide variety of primary abdominal wounds such as those created during elective or acute surgery, surgery for benign or malignant disease and

For peer review only

bowel resection of obstetric procedures. Such studies will always record the patient group which will be important to consider when looking at the results of the measures.

True patient-centred outcome measures require full consideration of patients' experiences and views' (7, 8). The main strength of this research, therefore, is the use of qualitative research methods to provide important insights into the under researched area of early issues related primary surgical wounds relating to practical wound management and patient experience. We adopted a purposeful sampling strategy to ensure that perspectives were captured from a range of participants in relation to their primary surgical abdominal wound (10). Data produced from the interviews were supplemented by an analysis of existing RCT outcomes to ensure a comprehensive list of issues were initially generated, and therefore acted as a method of triangulation to increase the plausibility and dependability of the interview data (11).

However, it is important to note that these measures have only been pre-tested in relation to primary surgical wounds. Wounds that are intentionally left open or wounds that have developed problems are likely to require dressings that have advanced practical properties that are tailored to the wound requirements (15). Although participants were purposefully sampled, most had had a dressing of some kind (94%). A prospective real-time survey of dressings has demonstrated that this reflects current practice (16). In addition, these measures have only been pre-tested in relation to abdominal surgical wounds. However, characteristics of wound healing in this area are likely to be consistent with many other parts of the body. Furthermore, these measures focus specifically on the experience of dressings - methods of wound closure (i.e. potentially leading to differential ease of removal of sutures or staples) may also affect patient experience, although this would require further investigation.

In summary, our measures can be completed both by patients and by health care professionals responsible for post-operative wound care. These measures will now be further developed to ensure they are appropriate and psychometrically-tested instruments, with a view to informing decision-making around dressings.

ACKNOWLEDGEMENTS

We would like to thank all participants for giving up their time to take part in this study, and members of the Bluebelle study team for their helpful comments on the earlier drafts of the measures.

For peer review only

CONTRUBITIONS

All members of the Bluebelle Study Group read and commented on the final version of the paper. Other roles are described below.

Lazaros Andronis co-applicant in the Bluebelle study grant (Health Economics); **Jane Blazeby** Chief investigator of the Bluebelle study grant (NIHR Bluebelle grant HTA - 12/200/04), led the whole study including chairing of management and executive meetings, supervised development of the questionnaires; **Natalie Blencowe** co-applicant in the Bluebelle study grant, provided feedback on earlier versions of the questionnaires and recruited patients/health care professionals for interviews; **Melanie Calvert** co-applicant in the Bluebelle study grant (patient-reported outcomes expertise); **Joanna Coast** co-applicant in the Bluebelle study grant (Health Economics lead); **Jenny L Donovan** co-applicant in the Bluebelle study grant (Qualitative research co-lead), co-designed and supervised the qualitative research; **Tim Draycott** co-applicant in the Bluebelle study grant (Obstetric surgical expertise and leadership) principal investigator and lead advisor for obstetric surgery; **Jo Dumville** provided feedback on earlier versions of the questionnaires; **Daisy Elliott** wrote the first draft of the manuscript and edited/finalised the paper, contributed to all aspects of qualitative data collection and qualitative data analysis and led development of the questionnaires; **Louise Flintoff** recruited patients/health care professionals for interviews; **Rachel Gooberman-Hill** co-applicant in the Bluebelle study grant (patient and public involvement lead); **Robert Longman** co-applicant in the Bluebelle study grant (Surgical expertise and leadership); **Rhiannon Macefield** conducted literature reviews and provided feedback on earlier versions of the questionnaires; **Laura Magill** co-applicant in the Bluebelle study grant, trial manager in Birmingham; **Jonathan Mathers** co-applicant in the Bluebelle study grant (Qualitative research co-lead); **Christel McMullan** contributed to all aspects of qualitative data collection and qualitative data analysis, provided feedback on earlier versions of the questionnaires; **David Messenger** recruited patients/health care professionals for interviews; **Charlotte Murkin** conducted pre-testing interviews; **Helen van der Nelson** recruited patients/health care professionals for interviews; **Tom Pinkney** co-applicant in the Bluebelle study grant (Surgical expertise and leadership); **Barnaby C Reeves** co-applicant in the Bluebelle study grant (Methodological lead), contributed to overall study design, and provided feedback on earlier versions of the questionnaires; **Chris A Rogers** co-

applicant in the Bluebelle study grant (Statistical lead); **Leila Rooshenas** contributed to all aspects of qualitative data collection and qualitative data analysis in Phase I and provided feedback on earlier versions of the questionnaires; **Andrew Torrance** co-applicant in the Bluebelle study grant; **Nicky J Welton** co-applicant in the Bluebelle study extension grant; **Mark Woodward** co-applicant in the Bluebelle study grant (Paediatric surgical expertise and leadership) and advisor on paediatric surgery; **Trudie Young** co-applicant in the Bluebelle study grant (Wound nursing specialist) and advisor on wound care.

COMPETING INTERESTS

None declared.

DISCLAIMER

The views expressed are those of the authors and not necessarily those of the MRC, NHS, NIHR or the Department of Health.

FUNDING

The Bluebelle study (Phase A) is funded by the National Institute for Health Research (NIHR) Health Technology Assessment (HTA) Programme (HTA - 12/200/04). JLD and JMB are NIHR Senior Investigators. JLD is also supported by the NIHR Collaboration for Leadership in Applied Health Research and Care (CLAHRC) West at University Hospitals Bristol NHS Foundation Trust. The Bluebelle study was undertaken with the support of the MRC ConDuCT-II Hub (Collaboration and innovation for Difficult and Complex randomised controlled Trials In Invasive procedures - MR/K025643/1).

ETHICAL APPROVAL

Ethical approval for this work was granted by the Camden and King's Cross Research Ethics Committee (14/LR/0640) on the 10th April 2014.

DATA SHARING STATEMENT

No additional data are available.

REFERENCES

For peer review only

1. Weiser TG, Regenbogen SE, Thompson KD, Haynes AB, Lipsitz SR, Berry WR, et al. An estimation of the global volume of surgery: a modelling strategy based on available data. The Lancet.372(9633):139-44.
2. Dumville JC, Gray TA, Walter CJ, Sharp CA, Page T. Dressings for the prevention of surgical site infection. The Cochrane Library. 2014.
3. Dumville JC, Coulthard P, Worthington HV, Riley P, Patel N, Darcey J, et al. Tissue adhesives for closure of surgical incisions. The Cochrane Library. 2014.
4. Sullivan Ta, Smith J, Kermode J, Mclver E, Courtemanche D. Rating the burn scar. Journal of Burn Care & Research. 1990;11(3):256-60.
5. Hollander JE, Singer AJ, Valentine S, Henry MC. Wound registry: development and validation. Ann Emerg Med. 1995;25(5):675-84.
6. Walter C, Dumville J, Sharp C, Page T. Systematic review and meta-analysis of wound dressings in the prevention of surgical-site infections in surgical wounds healing by primary intention. Br J Surg. 2012;99(9):1185-94.
7. Wilburn J, McKenna SP, Twiss J, Kemp K, Campbell S. Assessing quality of life in Crohn's disease: development and validation of the Crohn's Life Impact Questionnaire (CLIQ). Qual Life Res. 2015;24(9):2279-88.
8. Bausewein C, Simon ST, Benalia H, Downing J, Mwangi-Powell FN, Daveson BA, et al. Implementing patient reported outcome measures (PROMs) in palliative care-users' cry for help. Health and quality of life outcomes. 2011;9(1):27.
9. Trujols J, Portella MJ, Iraurgi I, Campins MJ, Siñol N, Cobos JPdL. Patient-reported outcome measures: Are they patient-generated, patient-centred or patient-valued? Journal of Mental Health. 2013;22(6):555-62.
10. Patrick DL, Burke LB, Gwaltney CJ, Leidy NK, Martin ML, Molsen E, et al. Content Validity—Establishing and Reporting the Evidence in Newly Developed Patient-Reported Outcomes (PRO) Instruments for Medical Product Evaluation: ISPOR PRO Good Research Practices Task Force Report: Part 1—Eliciting Concepts for a New PRO Instrument. Value Health. 2011;14(8):967-77.
11. Patrick DL, Burke LB, Gwaltney CJ, Leidy NK, Martin ML, Molsen E, et al. Content Validity—Establishing and Reporting the Evidence in Newly Developed Patient-Reported Outcomes (PRO) Instruments for Medical Product Evaluation: ISPOR PRO Good Research Practices Task Force Report: Part 2—Assessing Respondent Understanding. Value Health. 2011;14(8):978-88.
12. Sprangers M, Cull A, Bjordal K, Groenvold M, Aaronson NK. The European Organization for Research and Treatment of Cancer approach to quality of life assessment: guidelines for developing questionnaire modules. Qual Life Res. 1993;2(4):287-95.
13. Sprangers M, Cull A, Groenvold M, Bjordal K, Blazeby J, Aaronson NK. The European Organization for Research and Treatment of Cancer approach to developing questionnaire modules: an update and overview. Qual Life Res. 1998;7(4):291-300.
14. Lasch KE, Marquis P, Vigneux M, Abetz L, Arnould B, Bayliss M, et al. PRO development: rigorous qualitative research as the crucial foundation. Qual Life Res. 2010;19(8):1087-96.
15. Andronis L, Blazeby J, Blencowe N, Calvert M, Coast J, Draycott T, et al. Bluebelle study (phase A): a mixed-methods feasibility study to inform an RCT of surgical wound dressing strategies. BMJ open. 2016;6(9):e012635.
16. Andronis L, Calvert M, Magill L, Mathers J, Pinkney T, Torrance A, et al. Feasibility work to inform the design of a randomized clinical trial of wound dressings in elective and unplanned abdominal surgery. The British Journal of Surgery. 2016;103(12):1738.
17. Glaser BG, Strauss AL. The Discovery of Grounded Theory. Aldine Transaction: USA; 1967.
18. Braun V, Clarke V. Using thematic analysis in psychology. Qualitative Research in Psychology. 2006;3(2):77-101.
19. Health UDo, Evaluation HSFCfD, Research, Health UDo, Evaluation HSFCfB, Research, et al. Guidance for industry: patient-reported outcome measures: use in medical product development to support labeling claims: draft guidance. Health and Quality of Life Outcomes. 2006;4:1-20.

For peer review only

20. Leidy NK, Vernon M. Perspectives on patient-reported outcomes. Pharmacoeconomics. 2008;26(5):363-70.
21. Beatty PC, Willis GB. Research Synthesis: The Practice of Cognitive Interviewing. Public Opin Q. 2007;71(2):287-311.
22. Amin M, Glynn F, Timon C. Randomized trial of tissue adhesive vs staples in thyroidectomy integrating patient satisfaction and Manchester score. Otolaryngology--Head and Neck Surgery. 2009;140(5):703-8.
23. Avşar AF, Üstüner I, Keskin HL, Öztürk Ö, Taş EE. 2-octylcyanoacrylate tissue adhesive versus polypropylene suture for skin closure of Pfannenstiel incision. J Turk Soc Obstet Gynecol. 2009;6:117-22.
24. Blondeel PN, Murphy JW, Debrosse D, Nix JC, Puls LE, Theodore N, et al. Closure of long surgical incisions with a new formulation of 2-octylcyanoacrylate tissue adhesive versus commercially available methods. The American journal of surgery. 2004;188(3):307-13.
25. Burke NG, Green C, McHugh G, McGolderick N, Kilcoyne C, Kenny P. A prospective randomised study comparing the jubilee dressing method to a standard adhesive dressing for total hip and knee replacements. Journal of tissue viability. 2012;21(3):84-7.
26. Chibbaro S, Tacconi L. Use of skin glue versus traditional wound closure methods in brain surgery: A prospective, randomized, controlled study. J Clin Neurosci. 2009;16(4):535-9.
27. Cosker T, Elsayed S, Gupta S, Mendonca A, Tayton K. Choice of dressing has a major impact on blistering and healing outcomes in orthopaedic patients. J Wound Care. 2005;14(1):27-9.
28. Dowson CC, Gilliam AD, Speake WJ, Lobo DN, Beckingham IJ. A prospective, randomized controlled trial comparing n-butyl cyanoacrylate tissue adhesive (LiquiBand) with sutures for skin closure after laparoscopic general surgical procedures. Surgical Laparoscopy Endoscopy & Percutaneous Techniques. 2006;16(3):146-50.
29. Gennari R, Rotmensz N, Ballardini B, Scevola S, Perego E, Zanini V, et al. A prospective, randomized, controlled clinical trial of tissue adhesive (2-octylcyanoacrylate) versus standard wound closure in breast surgery. Surgery. 2004;136(3):593-9.
30. Greene D, Koch RJ, Goode RL. Efficacy of octyl-2-cyanoacrylate tissue glue in blepharoplasty: a prospective controlled study of wound-healing characteristics. Arch Facial Plast Surg. 1999;1(4):292-6.
31. Holm C, Petersen JS, Grønbœk F, Gottrup F. Effects of occlusive and conventional gauze dressings on incisional healing after abdominal operations. Eur J Surg. 1998;164(3):179-83.
32. Kent A, Liversedge N, Dobbins B, McWhinnie D, Jan H. A prospective, randomized, controlled, double-masked, multi-center clinical trial of medical adhesives for the closure of laparoscopic incisions. J Minim Invasive Gynecol. 2014;21(2):252-8.
33. Keng TM, Bucknall TE. A clinical trial of tissue adhesive (histoacryl) in skin closure of groin wounds. The Medical journal of Malaysia. 1989;44(2):122-8.
34. Khan R, Fick D, Yao F, Tang K, Hurworth M, Nivbrant B, et al. A comparison of three methods of wound closure following arthroplasty. Bone & Joint Journal. 2006;88(2):238-42.
35. Law N, Ellis H. Exposure of the wound-a safe economy in the NHS. Postgrad Med J. 1987;63(735):27-8.
36. Lawrentschuk N, Falkenberg MP, Pirpiris M. Wound blisters post hip surgery: A prospective trial comparingdressings. ANZ J Surg. 2002;72(10):716-9.
37. Livesey C, Wylde V, Descamps S, Estela C, Bannister G, Learmonth I, et al. Skin closure after total hip replacement. Bone & Joint Journal. 2009;91(6):725-9.
38. Michie DD, Hugill JV. Influence of occlusive and impregnated gauze dressings on incisional healing: a prospective, randomized, controlled study. Ann Plast Surg. 1994;32(1):57-64.
39. Ong C, Jacobsen A, Joseph V. Comparing wound closure using tissue glue versus subcuticular suture for pediatric surgical incisions: a prospective, randomised trial. Pediatr Surg Int. 2002;18(5-6):553-5.

For peer review only

40. Pronio A, Di Filippo A, Narilli P, Caporillli D, Vestri A, Ciamberlano B, et al. Closure of cutaneous incision after thyroid surgery: a comparison between metal clips and cutaneous octyl-2-cyanoacrylate adhesive. A prospective randomized clinical trial. Eur J Plast Surg. 2011;34(2):103-10.
41. Shamiyeh A, Schrenk P, Stelzer T, Wayand WU. Prospective Randomized Blind Controlled Trial Comparing Sutures, Tape, and Octylcyanoacrylate Tissue Adhesive for Skin Closure After Phlebectomy. Dermatol Surg. 2001;27(10):877-80.
42. Sniezek PJ, Walling HW, Debloom JR, Messingham MJ, Vanbeek MJ, Kreiter CD, et al. A Randomized Controlled Trial of High-Viscosity 2-Octyl Cyanoacrylate Tissue Adhesive versus Sutures in Repairing Facial Wounds Following Mohs Micrographic Surgery. Dermatol Surg. 2007;33(8):966-71.
43. Ravenscroft M, Harker J, Buch K. A prospective, randomised, controlled trial comparing wound dressings used in hip and knee surgery: Aquacel and Tegaderm versus Cutiplast. The Annals of The Royal College of Surgeons of England. 2006;88(1):18-22.
44. Romero P, Frongia G, Wingerter S, Holland-Cunz S. Prospective, randomized, controlled trial comparing a tissue adhesive (Dermabond™) with adhesive strips (Steri-Strips™) for the closure of laparoscopic trocar wounds in children. Eur J Pediatr Surg. 2011;21(03):159-62.
45. Vogt KC, Uhlyarik M, Schroeder TV. Moist wound healing compared with standard care of treatment of primary closed vascular surgical wounds: A prospective randomized controlled study. Wound Repair Regen. 2007;15(5):624-7.
46. Wikblad K, Anderson B. A comparison of three wound dressings in patients undergoing heart surgery. Nurs Res. 1995;44(5):312.
47. Wynne R, Botti M, Stedman H, Holsworth L, Harinos M, Flavell O, et al. Effect of three wound dressings on infection, healing comfort, and cost in patients with sternotomy wounds: a randomized trial. CHEST Journal. 2004;125(1):43-9.

For peer review only

Interview Topic Guide: Phase 1: Generation of relevant issues

Background, interviewee details and ice breaker

- Interviewee background and details of procedure that the interviewee has had (type of procedure elective or emergency? have you had before?, when, where, length of stay in hospital, recovery period).
    - *Just to give me some background, can you just tell me a little bit about the procedure that you had, the surgical procedure?*
    - *How have you been, in terms of recovery? Length of stay? When came home?*
    - *Is this your first?*

Expectations/experiences of wound care

- *Did/do you have any expectations about whether or not you would have a dressing? Where do you think these expectations came from?*
- *What kind of dressing? When did they take it off? [home/hospital?] Same one? How did you find having the dressing? Why do you think you had/did not have a dressing? Do you think it had any impact on your wound healing?*
- *Why do you think they do/don't use dressings?*
- *Thoughts/reactions to* ***alternative*** *wound management methods (i.e. dressing or no dressing in relation to what patient has experienced/what patient expects). (Explore patient thoughts on impact this may have on previously mentioned issues (e.g. recovery, symptoms, practicalities).*

Patient perspectives on a trial of dressing type

Okay, the basis of this study is like I said, we're trying to find out if dressing the wounds is helpful or not. In a study, patients would be randomly allocated by chance to either receive dressings or receive no dressings. The doctor wouldn't decide and the patient wouldn't decide. What we're trying to find out is, in patients who have had a similar type of surgery, if we have one group of patients that have them on so and another group of patients that don't, then can we compare them to see if there's any difference between the groups.

- *Do you think you would participate? (Reasons why/why not? Any reservations?)*
- *Can you imagine your family and/or friends would participate in a study like this?*
- *What kinds of things do you think you might consider, when deciding whether or not to take part? Do you think you would have any questions about the study? What kind of things might you want to know about in advance?*
- *How do you think you would feel about random allocation to dressing type, specifically the possibility of receiving no wound dressing? [explain randomisation to patients, then ask…] If you were in a group that didn't receive a dressing, do you think you would have thought about your care differently?*
- *In paediatrics, they don't use dressings. Changes impression?*
- *What about a glue dressing?*
- *Can you think of any potential problems we might come across if we were doing this study, any practical problems or any difficulties we might come across?*
- *Perspectives on important outcomes to include in a trial of dressing use. We would like this study to help us answer the question of whether dressings should be used in patients such as yourself (and if so, what type of dressing is best). What do you think are the important factors we should consider when making any future recommendations on dressing use? Satisfaction? Infection? Healing times?*

Closing

- Summarise key points
- Any further questions?
- Thank patient for their time and explain how they have helped.

For peer review only - http://bmjopen.bmj.com/site/about/guidelines.xhtml

Interview Topic Guide: Phase 3 – Pre-testing

Introduction

- As you know, we are going to audio-record you whilst you complete the questionnaire that we have developed. During this, we would like to understand your opinion of the questions, in terms of the language used and the relevance to your post-operative experience. It is important to remember that there are no right or wrong answers, but that this will help us to understand how the questionnaire should be developed. This should take no longer than an hour, although you can stop the discussion at any time. The information that you provide is not going to be fed back to your clinical team.
- Complete consent form and data collection form
- Any questions?

Background (procedure and recovery)

- Could you tell me a little bit about what you had done? *(Probe: When? Where? Elective/emergency?)*
- How has recovery been for you so far? *(Probe: How feeling? Length of stay? When expected to go home?)*
- How has the wound been healing? *(Probe: How does wound feel? Any problems?)*

PWMQ: Can we work through the questionnaire as you complete it, with you explaining how you understand the items and what you will put for this question.

- DT to not clarify meaning, but just repeat question.
- If P uncertain, try to understand why.
- Note any potential issues to be discussed after completion.

PWMQ: Understanding responses to each question

- Can you tell me in your own words what that question was asking? *(Probe: What does the word XX mean to you? Are there any other ways you would describe it?)*
- What does [not at all/a little/quite a bit/very much] mean to you?
- Was it easy to choose an answer?
- Explore where participants have indicated confusion

After completion:

- What did you think of the questionnaire?
- Were the questions relevant to your experience? *(Probe: Are there any others that should be included?)*
- Any difficulties filling in questions?
- Do you think it captures your experience of your wound in the past 24 hours?
- What does the word "practical wound management" mean to you?
- Do you have any other suggestions on how the questionnaire could be improved? *(Probe: alternative wording, additional questions, response categories, layout/presentation, length of questionnaire?)*
- You have given your answers based on the past 24 hours. Would your answers differ if you were thinking about your entire recovery from surgery? *(Probe: When do you think would be the best time to complete questionnaires like these?)*

Closing

- Summarise key points
- Any further questions?
- Thank patient for their time and explain how they have helped.

For peer review only

Table 1: Item tracking matrix of all issues identified

Category	Issue	Identified by				Additional comments	Included in questionnaire?	
		Interviews			Literature			
		DE (n=28)	LR (n=19)	CM (n=11)	(n=26 RCTs)		If yes, questionnaire item	Why not included
Wound comfort	Itchiness	✓	✓	✓	✓	CM: Described by patients as an outcome (looking at the wound to check if it is irritated, inflamed, etc)<br>DE: Similar to inflammation ('burning'	Q1: Has the wound been itchy?	
	Pain	✓	✓	✓	✓	DE: Described as 'sore', 'hurt', 'tender', 'uncomfortable'.<br>DE: Sometimes mentioned in the context of a numerical scale.<br>RM: Described as 'burning pain referring to a dressing-related sensation felt under the dressing' in one study<br>RM: Also 'tenderness'<br>CM: 'sore', 'painful' – discussed in terms of dressing removal	Q2: Has the wound been painful?	
	Presence of pulling sensation	✓			✓	LR: Described as wound 'being able to breathe', 'stuffiness'	Q3: Has the wound had a pulling sensation?	
	Tightness of wound	✓	✓		✓		Q4: Has the wound felt tight?	
	Wound comfort (overall or unspecified)				✓	RM: Also measured as 'Discomfort' in the literature<br>RM: Includes 'Discomfort with skin problems'		Excluded as covered in Q1 – Q4
Exudate and its impact	Whether there was any exudate	✓	✓	✓	✓		Q5: Has the wound leaked?	
	Type of exudate (blood, other)	✓	✓	✓	✓	DE: Described as 'mess', 'manky', 'leaking', 'gunge', 'oozing', 'soaking', 'brown mess'<br>LR: 'moistness', Ooziness', 'dampness'<br>RM: Described as 'discharge' 'fluid' 'oozing'<br>CM: 'Seeping'	Q5: If so, was it: clear fluid? cloudy fluid? Blood-stained fluid? thick and yellow/green fluid?	
	Whether exudate marks bedding/clothing	✓	✓	✓		DE: Described as 'stains', LR: 'manked up clothing'	Q6: Has the leakage resulted in changed bedding/ clothes?	
	Degree dressing absorbs exudate	✓	✓	✓	✓		Q7: How would you describe the wettest dressing?	
	Whether additional dressing required	✓	✓	✓	✓	RM: Includes reasons for dressing changes<br>CM: use two dressings when oozing is important, don't want to take original dressing off	Q8: Has a dressing or glue been put on the wound (or replaced)?	
	Anxiety associated with exudate	✓	✓	✓				Excluded as captured in Q15: "Have you felt any anxiety about your wound?"
Allergic reactions to the dressing	Any allergic reactions to dressing/blistering	✓	✓	✓	✓	RM: Also include skin damage/injury	b) has the wound blistered?	
Dressing removal	Whether dressing comes off	✓	✓	✓			Q9: Has the dressing or glue come off or been removed	
	Whether dressing needs to be taken off	✓	✓	✓		CM: patient or partner	Q9 Q9: Has the dressing or glue come off or been removed?	

For peer review only

	(if so, by patient or professional)						If "Yes", was it taken off by a doctor/nurse/other health specialist?	
	Whether travel is required to change/remove dressing (i.e. seeing nurse or GP/post op visits)	✓	✓					Not relevant to early post-operative period
	Any discomfort during removal	✓	✓	✓	✓		Q10 Was there any discomfort when removing the dressing?	
	Any pain during removal	✓	✓	✓		DE: Causes 'pain', skin is 'tender', 'sore', pulls hairs, sticks to skin)<br>RM: 'Pain on removal of the dressing'	Q11 Was there any pain when removing the dressing?	
**Wound protection**	Dressings protecting the wound				✓		Q12 Has the wound felt protected? (i.e. from catching on anything or being knocked)	
	Whether dressing/wound rubs on clothes	✓	✓	✓		LR: Awkwardness of wearing clothes over dressing		
	Whether dressing/wound catches on other things	✓	✓	✓		CM: bedsheets		
**Impact on daily activities**	Ability to get back to work	✓	✓				Q13: Have you been able to perform everyday tasks? (i.e. showering/ bathing, getting dressed)	
	Ability to shower/bathe	✓	✓	✓	✓	RM: Described as 'Ability to facilitate personal hygiene' in one study<br>RM: Described as 'Appreciation of possibility to shower' in one study. Also 'satisfaction with the possibility to wash oneself'		
	Ease of movement (e.g sitting, walking, stairs)	✓	✓		✓	DE: Standing up, walking<br>RM: Described as 'Ability to facilitate mobility' in the literature. Also 'Does the dressing limit you in movement?'<br>DE and LR: Includes sneezing/coughing		
	Ability to perform everyday tasks (e.g self-care)	✓	✓	✓		DE: Washing/self-care, driving, walking, housework, cooking, exercise		
	Ease of getting dressed	✓						
	Going to the toilet		✓	✓				
	Self-management of wound		✓	✓	✓	RM: 'Ease of managing wound' was a PRO measured in the first 3 weeks after surgery on a 1-10 scale in one study		
	Change to usual clothing		✓	✓				
	Overall recovery				✓	RM: not a PRO (surgeon rating)		
**Ease of movement**	(Dis)comfort when sitting	✓	✓	✓			Q14: Have you been able to move around easily?	
	(Dis)comfort when lying	✓	✓					

For peer review only

	(Dis)comfort whilst sleeping/sleep quality	✓	✓					
	(Dis)comfort whilst moving	✓	✓	✓		CM: related to tightness of dressing		
**Anxiety about the wound**	Feeling of security/safeness (in relation to the wound?)	✓	✓	✓			Q15: Have you felt any anxiety about your wound?	
	Feeling of vulnerability		✓	✓				
	Not having to worry about wound/dressing	✓	✓	✓		DE: 'You can just forget about it, you don't have to think about		
	Feeling protected	✓	✓	✓				
	Stress levels/psychological discomfort/anxiety	✓	✓					
	Feeling constricted by dressing	✓	✓					
	Cleanliness of environment	✓	✓	✓		DE: Described as 'hygiene', 'coming into contact with bugs'		
	Fear of infection	✓	✓	✓				
	Anxiety about bodily contents spilling out/wound bursting open	✓	✓	✓		LR: Described as 'coming open', 'split apart', 'rupture'		
**Satisfaction with dressing/ not having a dressing**	Satisfaction with (appearance of) dressing	✓	✓		✓	LR: 'Neatness', 'prominence'<br>RM: Measured as 'How satisfied overall do you feel with your dressing?'	Q16: Have you felt satisfied with having/not having a dressing?	
	Whether patient would prefer to see the wound	✓	✓			DE: Anxiety/reassurance/fear/discomfort<br>RM: Described as 'how well the incision could be seen under the dressing' and 'transparency'		
	Degree dressing fits contours of the skin/clothing	✓	✓		✓	RM: Described as 'Conformability of the dressing to the wound' in one study		
	Dignity			✓		CM: Having a dressing gives more dignity		
	Confidence			✓		CM: Confidence to walk around without having to worry		
	Appreciation of absence of bandage				✓			
	How long dressing stays on	✓	✓	✓	✓	CM: 'the longer you leave a dressing on the harder it is to get off'		
	Ease of removal	✓	✓	✓	✓	RM: 'Ease of dressing application' was measured in one study but this was rated by a surgeon not patient<br>LR: Any exudate from removal<br>DE, CM,: Any remnants remaining after removal ('bits of glue')		
	Degree of stickiness	✓	✓	✓	✓	CM: stitches stick to legs when sitting (gynae)		

For peer review only

						DE: Mostly considered to be positive, but sometimes makes removal tricky<br>DE: Described as: 'It stays in place and doesn't wriggle up'<br>RM: Described as 'Dressing integrity' in one study also 'How much the dressing had loosened'<br>CM: dressings don't stick well because of body hair		
	Awareness of dressing/wound	✓	✓	✓	✓			
	If patient reapplies, ease of reapplying dressing	✓	✓		✓			
	Whether additional support or materials provided	✓	✓	✓				
	Whether patient uses own dressing	✓	✓	✓				
	If patient reapplies, ease of reapplying dressing	✓	✓		✓			
	Overall satisfaction/satisfaction with overall experience				✓			
**Wound appearance**	Perceptions of healing	✓	✓	✓	✓	DE: Described as 'healing very nicely', 'rate of healing'<br>LR: Sub-codes – 'quality of healing', 'speed of healing', 'whether healed or not', 'suggested as main outcome'. Patients often discuss scab formation when talking about healing.<br>RM: Measured as 'Effectiveness (wound healing)' in one study 1= well healed, 3=poorly healed, not a PRO. Also measured as a PRO in another study 'Has your wound healed?'<br>CM: Healing could be a standalone category (one of the outcomes): definition of healing, healing time		Relevant to longer term outcomes of wound healing (not relevant within first days of surgery)<br>Patients' wounds not visible if dressing applied
	Bruising	✓	✓			DE: Described as 'black and blue'		
	Colour	✓	✓	✓	✓	DE: Described as 'purple'/'pink'/'grey'/'red' (see inflammation')<br>LR: 'Black and bluish' (also fit under 'bruising', as above)<br>RM: Redness<br>CM: Colour was talked about more in the context of healing, ie as an indicator (for the patients) of whether or not the wound was healing		
	Cosmesis/aesthetics		✓		✓	RM: Described as 'cosmesis', 'cosmetic outcome' and 'cosmetic result' in the literature		

For peer review only

	Scars	✓	✓	✓	✓	DE: A long term measure<br>RM: Pigmentation, scar colour, prescence of inflammation, suppleness or pliability, scar height or evenness with the surrounding skin, using modified Vancouver Burn Assessment Scale		
	Size	✓	✓	✓				
	Scabbing	✓	✓	✓		DE: Associated with healing		
	Inflammation/swellin g	✓	✓	✓		DE: Described as 'burning'<br>CM: Described by patients as an outcome		
	Overall appearance of wound	✓	✓	✓		DE: Described as 'unsightly', 'neat', 'tidy', 'messy' LR: 'ugly'<br>CM: 'Smooth'		
	Maceration of the skin				✓	RM: not a PRO		
	Satisfaction with the appearance of the wound				✓			
	Whether patient would prefer to see the wound	✓	✓					

For peer review only

Outcome as described by author	Wording used to measure outcome (where reported)	Who reported the outcome	Rating/measurement scale	Assessment time point	Study reference	Study source *
Pain	-	Patient reported	0 to 10 cm VAS	Day 1, Day 10	Amin 2009	2,3
Cosmesis of wound	Cosmesis of wound	Patient reported	Poor 0 5 Excellent 10	3 months post-op	Amin 2009	2,3
Ability to shower same day	Ability to shower same day	Patient reported	Poor (0) Satisfactory (1) Excellent(2)	3 months post-op	Amin 2009	2,3
Need visit GP for wound care	Need visit GP for wound care	Patient reported	"Could have done without; Didn't mind; N/A"	3 months post-op	Amin 2009	2,3
Pain on removing clips	Pain on removing clips	Patient reported	Yes/No	3 months post-op	Amin 2009	2,3
Pain/Tightness of wound after 3 months	Pain/Tightness of wound after 3 months	Patient reported	Slight(0)Moderate(1)Significant(2)	3 months post-op	Amin 2009	2,3
Overall comfort with wound	Overall comfort with wound	Patient reported	Poor(0)Satisfactory(1)Excellent(2)	3 months post-op	Amin 2009	2,3
Allergic reactions	Allergic reactions	Patient reported	Yes(0)No(1)	3 months post-op	Amin 2009	2,3
Overall satisfaction	Overall satisfaction	Patient reported	Poor 0; 5; 10 Excellent	3 months post-op	Amin 2009	2,3
Cosmetic appearance	How would you evaluate your skin stitches after the operation?	Patient reported	1 (very poor) 2 (poor) 3 (medium) 4 (good) 5 (very good)	Day 40 post-op	Asvar 2009	2
Satisfaction	What is your satisfaction?	Patient reported	1 (very poor) 2 (poor) 3 (medium) 4 (good) 5 (very good)	Day 40 post-op	Asvar 2009	2
Perceived patient satisfaction	-	Physician reported	"Ratings"	Day 10	Blondeel 2004	2,3
Cosmesis	-	Patient reported	"Ratings" – unspecified categories but included "outstanding"	Day 10	Blondeel 2004	2,3
Overall comfort	-	Patient reported	"Ratings"	Day 10	Blondeel 2004	2,3

* 1= Cochrane 2011 dressings review; 2= Cochrane 2014 tissue adhesive review; 3=Chow 2010 tissue adhesive review; 4=additional studies provided by authors of the Cochrane dressings review update

For peer review only

Outcome as described by author	Wording used to measure outcome (where reported)	Who reported the outcome	Rating/measurement scale	Assessment time point	Study reference	Study source *
Ability to shower	-	Patient reported	"Ratings"	Day 10	Blondeel 2004	2,3
Dressing changes	-	Patient reported	"Ratings"	Day 10	Blondeel 2004	2,3
Tension at the wound	-	Patient reported	"Ratings"	Day 10	Blondeel 2004	2,3
Hygiene problems	-	Patient reported	"Ratings"	Day 10	Blondeel 2004	2,3
Allergic reaction	-	Patient reported	"Ratings"	Day 10	Blondeel 2004	2,3
Overall satisfaction	-	Patient reported	"Ratings"	Day 10	Blondeel 2004	2,3
Cosmesis	-	Physician reported	validated Modified Hollander Instrument	Day 30	Blondeel 2004	2,3
Dressing changes	-	Observer reported	n/a	unspecified	Burke 2012	4
Incidence of blistering	-	Observer reported	n/a	unspecified	Burke 2012	4
Cosmetic outcome	-	Unspecified	Scale, 1-10	Unclear when specific outcomes were recorded – several follow ups at Day 1, 3, 5-7 and 2 weeks, 1, 3, 6, 12 months	Chibbaro 2009	2,3
Wound management by the patients	-	Unspecified	Unspecified	Unclear when specific outcomes were recorded – several follow ups at Day 1, 3, 5-7 and 2 weeks, 1, 3, 6, 12 months	Chibbaro 2009	2,3
Satisfaction	-	Patient reported	Scale, 1-10	Unclear when specific outcomes were recorded – several follow ups at Day 1, 3,	Chibbaro 2009	2,3

* 1= Cochrane 2011 dressings review; 2= Cochrane 2014 tissue adhesive review; 3=Chow 2010 tissue adhesive review; 4=additional studies provided by authors of the Cochrane dressings review update

For peer review only

Outcome as described by author	Wording used to measure outcome (where reported)	Who reported the outcome	Rating/measurement scale	Assessment time point	Study reference	Study source *
				5-7 and 2 weeks, 1, 3, 6, 12 months		
Appreciation of possibility to shower	-	Unspecified	Unspecified	Unclear when specific outcomes were recorded – several follow ups at Day 1, 3, 5-7 and 2 weeks, 1, 3, 6, 12 months	Chibbaro 2009	2,3
Appreciation of absence of head bandage	-	Unspecified	Unspecified	Unclear when specific outcomes were recorded – several follow ups at Day 1, 3, 5-7 and 2 weeks, 1, 3, 6, 12 months	Chibbaro 2009	2,3
Number of dressing changes	-	Collected by surgeons, nurses and junior medical staff	n/a	during hospital stay	Cosker 2005	1
Blistering	-	Collected by surgeons, nurses and junior medical staff	n/a	during hospital stay	Cosker 2005	1
When the dressing required changing	-	Collected by surgeons, nurses and junior medical staff	n/a	during hospital stay	Cosker 2005	1
Reason for dressing change	-	Collected by surgeons, nurses and junior medical staff	n/a	during hospital stay	Cosker 2005	1
Number of post operative days	-	Collected by surgeons, nurses and junior medical staff	n/a	during hospital stay	Cosker 2005	1

* 1= Cochrane 2011 dressings review; 2= Cochrane 2014 tissue adhesive review; 3=Chow 2010 tissue adhesive review; 4=additional studies provided by authors of the Cochrane dressings review update

For peer review only

Outcome as described by author	Wording used to measure outcome (where reported)	Who reported the outcome	Rating/measurement scale	Assessment time point	Study reference	Study source *
Volume of wound exudate	-	Collected by surgeons, nurses and junior medical staff	Amount of seepage through the dressing and the apparent volume found on the wound after the dressing was removed	during hospital stay	Cosker 2005	1
Satisfaction with wound closure method	-	Patient reported	Satisfied / Dissatisfied	24 to 48 hrs, 4 to 6 weeks, 3 months post-op	Dowson 2006	2,3
Satisfaction with appearance of the wound	-	Patient reported	Satisfied / Dissatisfied	24 to 48 hrs, 4 to 6 weeks, 3 months post-op	Dowson 2006	2,3
Degree of pain	-	Patient reported	Scale, 1-10	First 3 weeks after surgery	Gennari 2004	3
Ease of managing wound	-	Patient reported	Scale, 1-10	First 3 weeks after surgery	Gennari 2004	3
Ability to take a shower	-	Patient reported	Scale, 1-10	First 3 weeks after surgery	Gennari 2004	3
Postoperative visits	-	Patient reported	Scale, 1-10	First 3 weeks after surgery	Gennari 2004	3
Use of dressings	-	Patient reported	Scale, 1-10	First 3 weeks after surgery	Gennari 2004	3
Comfort	-	Interview with physician	Unspecified	1, 2, & 4 weeks post-op	Greene 1999	2,3
Presence of a pulling sensation	-	Interview with physician	Unspecified	1, 2, & 4 weeks post-op	Greene 1999	2,3
Appreciation of the lack of suture removal	-	Interview with physician	Unspecified	1, 2, & 4 weeks post-op	Greene 1999	2,3
Discomfort in connection with removal of dressing	-	Unspecified	Unspecified	during hospital stay	Holm 1998	1
Number of dressing changes	-	Observer reported	n/a	during hospital stay	Holm 1998	1

* 1= Cochrane 2011 dressings review; 2= Cochrane 2014 tissue adhesive review; 3=Chow 2010 tissue adhesive review; 4=additional studies provided by authors of the Cochrane dressings review update

For peer review only

Outcome as described by author	Wording used to measure outcome (where reported)	Who reported the outcome	Rating/measurement scale	Assessment time point	Study reference	Study source *
Adhesion of dressing to the skin	-	Observer reported	Unspecified	daily inspection until discharge	Holm 1998	1
Cosmetic result	-	Observer reported	1 to 5 (higher=better)	3 months post-op	Holm 1998	1
Width of the scar	-	Observer reported	1 to 5 (higher=better)	3 months post-op	Holm 1998	1
Downbinding of the scar	-	Observer reported	1 to 5 (higher=better)	3 months post-op	Holm 1998	1
Colour of the scar	-	Observer reported	1 to 5 (higher=better)	3 months post-op	Holm 1998	1
Elevation of the scar	-	Observer reported	1 to 5 (higher=better)	3 months post-op	Holm 1998	1
Cosmetic outcome	-	Observer reported	1 to 5 (higher=better)	3 months post-op	Holm 1998	1
Supposed inconvenience of the scar	-	Observer reported	1 to 5 (higher=better)	3 months post-op	Holm 1998	1
Exudate	-	Observer reported	Unspecified	daily inspection until discharge	Holm 1998	1
Leakage	-	Observer reported	Unspecified	daily inspection until discharge	Holm 1998	1
Transparency	-	Observer reported	Unspecified, but included milky and slightly milky	daily inspection until discharge	Holm 1998	1
Dressing changes	-	Observer reported	No. of days stayed in place	unspecified	Holm 1998	1
Reasons for dressing changes	-	Observer reported	Unspecified	unspecified	Holm 1998	1
Maceration of the skin	-	Observer reported	Unspecified	unspecified	Holm 1998	1
Post operative wound infection	-	Observer reported	Unspecified	unspecified	Holm 1998	1
Comfort	-	Observer reported	Yes/No	1 week and 1 month	Keng 1989	2
Cosmesis	-	Observer reported	1 (poor) to 5 (excellent)	1 week and 1 month	Keng 1989	2

* 1= Cochrane 2011 dressings review; 2= Cochrane 2014 tissue adhesive review; 3=Chow 2010 tissue adhesive review; 4=additional studies provided by authors of the Cochrane dressings review update

For peer review only

For peer review only - http://bmjopen.bmj.com/site/about/guidelines.xhtml

Outcome as described by author	Wording used to measure outcome (where reported)	Who reported the outcome	Rating/measurement scale	Assessment time point	Study reference	Study source *
Satisfaction with the incision closure	-	Patient verbal report; response recorded by staff	Either “satisfied” or “dissatisfied”	3 month follow up	Kent 2014	2
Overall appearance	-	Patient verbal report; response recorded by staff	Either “satisfied” or “dissatisfied”	3 month follow up	Kent 2014	2
Satisfaction with the techniques of skin closure	-	Patient reported	VAS between 0 and 100, where 100 represented maximal satisfaction.	Between 8 and 12 weeks post-op	Khan 2006	2,3
Dressing or superficial wound discomfort	-	Unspecified	Linear analogue scale	5 days post -op	Law 1987	1
Dressing preference	-	Unspecified	Unspecified	unspecified	Law 1987	1
Wound infection	-	Unspecified	Discharge of purulent material	unspecified	Law 1987	1
Number of dressing changes	-	Unspecified	n/a	unspecified	Law 1987	1
Quality of the final scar	-	Unspecified	Unspecified	unspecified	Law 1987	1
Blisters	-	Observer reported	Defined as any lifting of the epidermis with underlying fluid	48 hours post-op and 5 days	Lawrentschuk 2002	1
Wound condition	-	Observer reported	Unspecified	5 days	Lawrentschuk 2002	1
Swelling	-	Observer reported	Measured thigh girth	48 hours post-op and 5 days	Lawrentschuk 2002	1
Wound infection	-	Observer reported	Unspecified	unspecified	Lawrentschuk 2002	1
Cosmetic appearance	-	Patient reported	100mm VAS (0 worst outcome, 100 best outcome)	3 months	Livesey 2009	2,3
Satisfaction with the scar	-	Patient reported	100mm VAS (0 extreme dissatisfaction, 100 complete satisfaction)	3 months	Livesey 2009	2,3

* 1= Cochrane 2011 dressings review; 2= Cochrane 2014 tissue adhesive review; 3=Chow 2010 tissue adhesive review; 4=additional studies provided by authors of the Cochrane dressings review update

For peer review only

Outcome as described by author	Wording used to measure outcome (where reported)	Who reported the outcome	Rating/measurement scale	Assessment time point	Study reference	Study source *
Appearance of the wound	-	Patient reported	5 point Likert scale (1=much better than expected, 2= better than expected, 3= as expected, 4= worse than expected, 5=much worse than expected)	3 months	Livesey 2009	2,3
Discomfort (pain in the past 48 hrs)	-	Patient reported	0 to 10 cm VAS	2-3 & 7-10 days post-op	Michie 1994	1
Discomfort (pain on removal of the dressing	-	Patient reported	0 to 10 cm VAS	2-3 & 7-10 days post-op	Michie 1994	1
Overall comfort	-	Patient reported	0 to 10 cm VAS	2-3 & 7-10 days post-op	Michie 1994	1
Wound itching (in the past 48hrs)	-	Patient reported	0 to 10 cm VAS	2-3, 7-10 days, 4 weeks, 7 months post-op	Michie 1994	1
Wound pulling (in the past 48 hrs)	-	Patient reported	0 to 10 cm VAS	2-3, 7-10 days, 4 weeks, 7 months post-op	Michie 1994	1
Conformability of the dressing to the wound	-	Surgeon reported	4 point rating scale (excellent, good, fair, poor)	7-10 days post-op	Michie 1994	1
Ability to contain exudate	-	Surgeon reported	4 point rating scale (excellent, good, fair, poor)	7-10 days post-op	Michie 1994	1
Ability to protect the wound	-	Surgeon reported	4 point rating scale (excellent, good, fair, poor)	7-10 days post-op	Michie 1994	1
Ability to facilitate mobility	-	Surgeon reported	4 point rating scale (excellent, good, fair, poor)	7-10 days post-op	Michie 1994	1
Ability to facilitate personal hygiene	-	Surgeon reported	4 point rating scale (excellent, good, fair, poor)	7-10 days post-op	Michie 1994	1
Overall impression of the incision	-	Patient reported	0 to 10 cm VAS	2-3 & 7-10 days post-op	Michie 1994	1
Evaluation of resulting scar	Pigmentation, scar colour, prescence of inflammation, suppleness/pliability,	Patient and surgeon reported	modified Vancouver Burn Assessment Scale (0 to 3 score)	4 weeks and 7 months post-op	Michie 1994	1

* 1= Cochrane 2011 dressings review; 2= Cochrane 2014 tissue adhesive review; 3=Chow 2010 tissue adhesive review; 4=additional studies provided by authors of the Cochrane dressings review update

For peer review only - http://bmjopen.bmj.com/site/about/guidelines.xhtml

Outcome as described by author	Wording used to measure outcome (where reported)	Who reported the outcome	Rating/measurement scale	Assessment time point	Study reference	Study source *
	scar height/evenness with the surrounding skin					
Ease of dressing application	-	Surgeon reported	Yes/No	2-3 & 7-10 days post-op	Michie 1994	1
Ease of dressing removal	-	Surgeon reported	Yes/somewhat difficult	2-3 & 7-10 days post-op	Michie 1994	1
Cosmetic result	-	Surgeon reported	4 point rating scale (excellent, good, fair, poor)	7-10 days post-op	Michie 1994	1
Infection	-	Surgeon reported	Unspecified	2-3 & 7-10 days post-op	Michie 1994	1
Pain upon palpation of the wound	-	Surgeon reported	3 point scale including Somewhat/no	7-10 days post-op	Michie 1994	1
Overall wound aspect	-	Surgeon reported	3 point scale including Excellent/good	7-10 days post-op	Michie 1994	1
Overall recovery	-	Surgeon reported	3 point scale including Excellent	7-10 days post-op	Michie 1994	1
Presence of small stitch abscess	-	Surgeon reported	Yes/no	7-10 days post-op	Michie 1994	1
Satisfaction with wound cosmesis	-	Parent reported	100mm VAS	2 to 3 weeks and 3 months follow up assessment (although unable to complete latter assessment as only 9 patients returned)	Ong 2002	2,3
Level of satisfaction with early postoperative management of the wound (regarding requirement of a return visit for medications, the possibility to wash oneself, the stuture removal)	-	Patient reported (verbal)	Numerical scale 0 to 10 (0-4, poor; 5-6, mild;7-8, good; 9-10, excellent)	15 days, 1, 3, 6 and 12 months	Pronio 2011	2

* 1= Cochrane 2011 dressings review; 2= Cochrane 2014 tissue adhesive review; 3=Chow 2010 tissue adhesive review; 4=additional studies provided by authors of the Cochrane dressings review update

For peer review only - http://bmjopen.bmj.com/site/about/guidelines.xhtml

Outcome as described by author	Wording used to measure outcome (where reported)	Who reported the outcome	Rating/measurement scale	Assessment time point	Study reference	Study source *
Discomfort	-	Unspecified	Unspecified	15 days, 1, 3, 6 and 12 months	Pronio 2011	2
Pain	-	Unspecified	Unspecified	15 days, 1, 3, 6 and 12 months	Pronio 2011	2
Pain	Pain resulting from dressing usage and not including mobilisation	Patient reported	Yes/no, 10 point VAS (0=no problems, 10=unbearable problems)	At dressing removal (mean 6 and 7 days)	Ravnskog 2001	4
Itching	-	Patient reported	Yes/no, 10 point VAS (0=no problems, 10=unbearable problems)	At dressing removal (mean 6 and 7 days)	Ravnskog 2001	4
Burning	Burning pain refering solely to a dressing-related sensation felt under the dressing	Patient reported	Yes/no, 10 point VAS (0=no problems, 10=unbearable problems)	At dressing removal (mean 6 and 7 days)	Ravnskog 2001	4
Discomfort during use of dressing	-	Patient reported	Yes/no, 10 point VAS (0=no problems, 10=unbearable problems)	At dressing removal (mean 6 and 7 days)	Ravnskog 2001	4
Pain at dressing removal	-	Patient reported	Yes/no, 10 point VAS (0=no problems, 10=unbearable problems)	At dressing removal (mean 6 and 7 days)	Ravnskog 2001	4
Skin damage (erythema, blisters or skin injury)		Observer reported	Small; 1-2cm, medium; 2-5cm, large<5cm	during hospital stay	Ravnskog 2001	4
Satisfaction with the cosmetic result	-	Patient reported	Dichotomous (satisfaied/dissatisfied)	Day 10 and day 90	Romero 2011	2
Pain at port sites	-	Surgeon reported	Unspecified	Day 10 and day 90	Romero 2011	2
Satisfaction with cosmetic result	-	Patients were asked by the senior dermatologist	1 (very satisfied) to 5 (not satisfied)	1 year post-op	Shamiyeh 2001	2,3
Degree of pain	-	Patient reported	Scale, 1-10	3 months post-op	Sniezek 2007	2,3
Ease of managing the surgical wound	-	Patient reported	Scale, 1-10	3 months post-op	Sniezek 2007	2,3

* 1= Cochrane 2011 dressings review; 2= Cochrane 2014 tissue adhesive review; 3=Chow 2010 tissue adhesive review; 4=additional studies provided by authors of the Cochrane dressings review update

For peer review only

Outcome as described by author	Wording used to measure outcome (where reported)	Who reported the outcome	Rating/measurement scale	Assessment time point	Study reference	Study source *
Ability to take a shower	-	Patient reported	Scale, 1-10	3 months post-op	Sniezek 2007	2,3
Overall satisfaction	-	Patient reported	Scale, 1-10	3 months post-op	Sniezek 2007	2,3
Cosmetic appearance	-	Patient reported	Scale, 1-10	3 months post-op	Sniezek 2007	2,3
Comfort of the dressing (discomfort at mobilization)	-	Patient reported	3 point scale (no discomfort at all, minor problems, severe discomfort)	Daily for 4 days after surgery	Vogt 2007	1
Comfort of the dressing (pain at dressing changes)	-	Patient reported	3 point scale (no discomfort at all, minor problems, severe discomfort)	Daily for 4 days after surgery	Vogt 2007	1
Comfort of the dressing (skin problems)	-	Patient reported	3 point scale (no discomfort at all, minor problems, severe discomfort)	Daily for 4 days after surgery	Vogt 2007	1
Signs of infection - redness, tenderness, swelling, exudates	-	Observer reported	Unspecified	2 weeks post-op	Vogt 2007	1
Wound complications - haematoma or persistent lymph oozing, surgical revision	-	Observer reported	Unspecified	2 weeks post-op	Vogt 2007	1
Length of hospital stay	-	Observer reported	n/a	n/a	Vogt 2007	1
Number of dressing changes	-	Observer reported	n/a	during postoperative stay	Vogt 2007	1
Patient comfort (difficulty in removing the dressings)	-	Nurse reported	Unspecified	Day 5	Wikblad 1995	1
Patient comfort (pain at dressing removal)	-	Nurse reported	3 point scale from "no pain at all" to "very painful"	Day 5	Wikblad 1995	1
Number of bandage changes	-	Nurse reported	n/a	Day 1 to day 5	Wikblad 1995	1
Reason for bandage changes	-	Nurse reported	n/a	Day 1 to day 5	Wikblad 1995	1

* 1= Cochrane 2011 dressings review; 2= Cochrane 2014 tissue adhesive review; 3=Chow 2010 tissue adhesive review; 4=additional studies provided by authors of the Cochrane dressings review update

For peer review only

For peer review only - http://bmjopen.bmj.com/site/about/guidelines.xhtml

Outcome as described by author	Wording used to measure outcome (where reported)	Who reported the outcome	Rating/measurement scale	Assessment time point	Study reference	Study source *
Effectiveness (wound healing)	-	Independent raters judging photograph	1=well healed (wound edges well together; a gap of <5% length of the incision allowed with no or slight redness), 2=partially healed (gaps >5% but <20% with slight to excessive redness), 3=poorly healed (gaps>20% with excessive redness)	Day 5 and 4 weeks after surgery	Wikblad 1995	1
Redness	-	Independent raters judging photograph	0=no redness, 1=slight redness, 2= excessive redness	Day 5 and 4 weeks after surgery	Wikblad 1995	1
Wound healing	Do you think the wound is well/partially/poorly healed?	Patient reported	3 point scale	Once a week after discharge for 3 weeks	Wikblad 1995	1
Skin changes	-	Patient reported	Unspecified	Once a week after discharge for 3 weeks	Wikblad 1995	1
Redness	Is the wound red?	Patient reported	Yes/No	Once a week after discharge for 3 weeks	Wikblad 1995	1
Swelling	Does the wound look swollen?	Patient reported	Yes/No	Once a week after discharge for 3 weeks	Wikblad 1995	1
Itching	Does the wound itch?	Patient reported	Yes/No	Once a week after discharge for 3 weeks	Wikblad 1995	1
Skin changes (erythema and blisters)	-	Independent raters judging photograph	n/a	Day 5	Wikblad 1995	1
Clinical utility (ability to allow ongoing evaluation of the incision)	How well the incision could be seen through the dressing	Nurse reported	1=good, 2=partially, 3=not atall	Day 1 to day 5	Wikblad 1995	1
How much the dressing had loosened	-	Nurse reported	graded scale from 1 to 3	Day 1 to day 5	Wikblad 1995	1
Treatment with antibiotics	-	Nurse reported	yes/no	4 weeks after surgery	Wikblad 1995	1

* 1= Cochrane 2011 dressings review; 2= Cochrane 2014 tissue adhesive review; 3=Chow 2010 tissue adhesive review; 4=additional studies provided by authors of the Cochrane dressings review update

For peer review only

Outcome as described by author	Wording used to measure outcome (where reported)	Who reported the outcome	Rating/measurement scale	Assessment time point	Study reference	Study source *
Safety (presence of infection - wound culture)	-	Clinical sample	n/a - lab sample	Day 5	Wikblad 1995	1
Dressing awareness	How aware are you of your dressing most of the time?	Patient reported	10 cm visual analogue scale with three anchors at 0,5 and 10cm	Day 1 to day 5	Wynne 2004	1
Movement limitation	Does the dressing limit you in moving about?	Patient reported	10 cm visual analogue scale with three anchors at 0,5 and 10cm	Day 1 to day 5	Wynne 2004	1
Comfort with removal	How comfortable do you feel during dressing changes?	Patient reported	10 cm visual analogue scale with three anchors at 0,5 and 10cm	Day 1 to day 5	Wynne 2004	1
Overall satisfaction	How satisfied overall do you feel with your dressing?	Patient reported	10 cm visual analogue scale with three anchors at 0,5 and 10cm	Day 1 to day 5	Wynne 2004	1
Wound healing - approximation	-	Observer reported	4 categories (total, partial;<2cm of superficial separation, moderate;>2cm of superficial separation, dehisced; complete separation of layers)	Day 1 to day 5	Wynne 2004	1
Wound healing - skin itegrity	-	Observer reported	3 categories (normal; pink no redness, inflammed; heat redness swelling, macerated within a 2.5cm border of the incision)	Day 1 to day 5	Wynne 2004	1
Wound infection	-	Observer reported	CDC criteria	unspecified	Wynne 2004	1
Dressing integrity	-	Observer reported	3 catergories - suture line exposed, poorly sealed, well sealed	unspecified	Wynne 2004	1
Experience with wound	Has your chest wound healed?	Patient reported	Yes/No	One month post-discharge	Wynne 2004	1
Antibiotic therapy	Has your doctor given you any antibiotics for	Patient reported	Yes/No	One month post-discharge	Wynne 2004	1

* 1= Cochrane 2011 dressings review; 2= Cochrane 2014 tissue adhesive review; 3=Chow 2010 tissue adhesive review; 4=additional studies provided by authors of the Cochrane dressings review update

For peer review only - http://bmjopen.bmj.com/site/about/guidelines.xhtml

Outcome as described by author	Wording used to measure outcome (where reported)	Who reported the outcome	Rating/measurement scale	Assessment time point	Study reference	Study source *
	your chest wound, since you left hospital?					
Experience with wound	Over the past month, has there been any fluid/discharge oozing from the chest wound? If so, how would you describe the fluid: watery, straw coloured; blood stained; pus (think yellow)	Patient reported	Yes/No	One month post-discharge	Wynne 2004	1
Experience with wound	Was a dressing required on your wound?	Patient reported	Yes/No	One month post-discharge	Wynne 2004	1
Experience with wound	Have you had any of the following problems with your chest wound? Redness, swelling, pain, tenderness	Patient reported	Yes/No	One month post-discharge	Wynne 2004	1
Experience with wound	Has your local doctor told you at any time your chest wound was infected?	Patient reported	Yes/No	One month post-discharge	Wynne 2004	1

* 1= Cochrane 2011 dressings review; 2= Cochrane 2014 tissue adhesive review; 3=Chow 2010 tissue adhesive review; 4=additional studies provided by authors of the Cochrane dressings review update

For peer review only

Note: Permission to use these measures must be obtained from the author (daisy.elliott@bristol.ac.uk). Work is being conducted to test the reliability, validity and sensitivity of these measures.

**Bluebelle dressing allocation:**	***Simple dressing***
**Study ID:**	
**Participant name:**	
**Date of surgery:**	
**Date completed:**	

Wound Experience Questionnaire

We are interested in how your wound(s) have healed since your operation and your experience of having a dressing, as part of the Bluebelle study. Please complete this short questionnaire yourself. You can complete the questionnaire as soon as you feel ready, but ideally this will be within four days of having your operation. If there is more than one wound, please respond **thinking about just one wound** – either the main one or another wound if there have been any concerns about how it has been healing.

When you have completed the questionnaire, please return it in the pre-paid envelope provided.

Section 1: Wound comfort

		Not at all	A little	Quite a bit	A lot
1.	Has your wound been itchy?	☐	☐	☐	☐
2.	Has your wound been painful?	☐	☐	☐	☐
3.	Has your wound had a pulling sensation?	☐	☐	☐	☐
4.	Has your wound felt tight?	☐	☐	☐	☐
5.	Has your wound been smelly?	☐	☐	☐	☐

Section 2: Removing the dressing

		Yes	No	
6.	Has the original dressing been removed/come off on its own?	☐	☐	→ *If "No" go to Section 3, Question 7*

If "Yes", how did it come off?

		Yes
a)	A doctor/nurse/other health professional	☐
b)	You/your partner/friend/family member	☐
c)	It came off on its own	☐

		Not at all	A little	Quite a bit	A lot
d)	Did you feel any pain when the dressing was removed?	☐	☐	☐	☐
e)	Did you feel any anxiety when the dressing was removed?	☐	☐	☐	☐

Section 3: Experience of having a dressing

		Not at all	A little	Quite a bit	A lot
7.	Has your dressing prevented you from showering or washing?	☐	☐	☐	☐
8.	Has your wound felt protected?	☐	☐	☐	☐
9.	Have you felt any anxiety about your wound in relation to your dressing(s)?	☐	☐	☐	☐
10.	Are you satisfied with your dressing(s)?	☐	☐	☐	☐

Additional comments:

……

……

……

Thank you for completing this questionnaire.

For peer review only

Note: Permission to use these measures must be obtained from the author (daisy.elliott@bristol.ac.uk). Work is being conducted to test the reliability, validity and sensitivity of these measures.

Bluebelle study

**Bluebelle dressing allocation:**	***Simple dressing***
**Study ID:**	
**Participant name:**	
**Date of surgery:**	
**Date completed:**	
**Completed by *(please tick)*:**	☐ Healthcare professional<br>☐ Participant<br>☐ Other (state):

Wound Management Questionnaire

To be completed by a healthcare professional up to 4 days after surgery

Or

To be completed by the participant up to 4 days after surgery if the participant is discharged before completion by a healthcare professional

If there is more than one wound, please respond **thinking about just one wound** – either the main one or another wound if there have been any concerns about how it has been healing. When you have completed the questionnaire, please return it in the pre-paid envelope provided.

Section 1: Wound leakage

In the past 24 hours…

	**Not at all**	**A little**	**Quite a bit**	**A lot**
1. Has fluid from the wound leaked through the dressing?	☐	☐	☐	☐

If "Not at all", go to Section 2, Question 3

	**Yes**	**No**	If "Yes", how many times?
2. Has the leakage required bedding or clothes to be changed?	☐	☐	____________

Section 2: Dressings

In the past 24 hours…

	**Yes**	**No**	
3. Has the original dressing been replaced?	☐	☐	If "No", questionnaire is complete
If "Yes", how many times?	__________		

4. Why was the dressing replaced?	**Yes** (tick all that apply)	
a) Routine change	☐	
b) The dressing was saturated	☐	
c) The wound was irritated	☐	
d) The wound was blistered	☐	
e) Another reason	☐	If "Yes", please specify what the reason was ______________________________

Additional comments:

……

……

……

Thank you for completing this questionnaire.

For peer review only

Table 1: Consolidated criteria for reporting qualitative research (COREQ)

	No	Item	Guide questions/description	Comment
**Domain 1: Research team and reflexivity**				
**Personal characteristics**	1	Interviewer/facilitator	Which author/s conducted the interview or focus group?	LR, DE, CMM and CM *(see pages 6 and 7)*
	2	Credentials	What were the researcher's credentials? e.*g. PhD, MD*	DE - BSc, PhD<br>LR - BSc, PhD<br>CMM - BA Hons, PgDip, MA PhD<br>CM – MB ChB BSc
	3	Occupation	What was their occupation at the time of the study?	DE - Qualitative Research Associate in Health Services Research<br>LR - Lecturer in Qualitative Health Science<br>CMM - Qualitative Research Fellow<br>CM - Research Fellow
	4	Gender	Was the researcher male or female?	Females
	5	Experience and training	What experience or training did the researcher have?	DE, LR and CMM have several years of experience conducting qualitative research. This has included completing many qualitative projects and attending training courses and workshops.
**Relationship with participants**	6	Relationship established	Was a relationship established prior to study commencement?	No
	7	Participant knowledge of the interviewer	What did the participants know about the researcher? *e.g. personal goals, reasons for doing the research*	The researchers introduced themselves, explained the purpose of the research and provided an information leaflet about the study
	8	Interviewer characteristics	What characteristics were reported about the interviewer/facilitator? *e.g. Bias, assumptions, reasons and interests in the research topic*	The researchers explained how qualitative research related to main Bluebelle trial
**Domain 2: study design**				
**Theoretical framework**	9	Methodological orientation and theory	What methodological orientation was stated to underpin the study? *e.g. grounded theory, discourse analysis, ethnography, phenomenology, content analysis*	Data were analysed thematically using techniques of constant comparison derived from grounded theory methodology *(see page 6)*

For peer review only - http://bmjopen.bmj.com/site/about/guidelines.xhtml

**Participant selection**	10	Sampling	How were participants selected? *e.g. purposive, convenience, consecutive, snowball*	Purposeful *(see pages 6 and 7)*
	11	Method of approach	How were participants approached? e.g. *face-to-face, telephone, mail, email*	Patients were approached face to face by healthcare professionals. Healthcare professionals were contacted by the researchers via email. *(See pages 6 and 7)*
	12	Sample size	How many participants were in the study?	Sixty-four patients and 15 health care professionals from abdominal general surgical specialities and obstetrics *(See Table 1 on pages 7/8)*
	13	Non-participation	How many people refused to participate or dropped out? Reasons?	Two patients were unable to take part due to poor health.
**Setting**	14	Setting of data collection	Where was the data collected? *e.g. home, clinic, workplace*	Patient interviews were conducted whilst patients were in hospital. Health professionals chose a location that was convenient for them (their workplace or a nearby café) or opted to do the interview over the telephone. *(See pages 6 and 7)*
	15	Presence of non-participants	Was anyone else present besides the participants and researchers?	The partners of patients sometimes sat with the patients but spoke very little; their comments were not included in the final analysis.
	16	Description of sample	What are the important characteristics of the sample? *e.g. demographic data, date*	Participants' full details are provided in Table 1, and key information is provided in the results section *(See pages 7/8)*
**Data collection**	17	Interview guide	Were questions, prompts, guides provided by the authors? Was it pilot tested?	A topic guide was developed (based on literature and views of health care professionals in the Bluebelle study team) to ensure that discussions covered the same core issues but with sufficient flexibility to allow new issues of importance to the informants to emerge. Although not piloted, it was adapted as analysis progressed to enable exploration of emerging themes. *(Topic guides are included in Additional File)*
	18	Repeat interviews	Were repeat interviews carried out? *If yes, how many?*	No repeat interviews were carried out
	19	Audio/visual recording	Did the research use audio or visual recording to collect the data?	Interviews were audio-recorded *(see page 6)*
	20	Field notes	Were field notes made during and/or after the	The researchers kept notes throughout data

For peer review only - http://bmjopen.bmj.com/site/about/guidelines.xhtml

			interview or focus group?	collection and analysis *(See page 8)*
	21	Duration	What was the duration of the interviews or focus group?	Interviews lasted an average of 25 minutes (range = 15–50 minutes). *(See page 8)*
	22	Data saturation	Was data saturation discussed?	Data collection continued until the team were confident that saturation had been reached *(See page 7)*
	23	Transcripts returned	Were transcripts returned to participants for comment and/or correction?	Transcripts were not returned to participants for comments or corrections
**Domain 3: analysis and findings**				
**Data analysis**	24	Number of data coders	How many data coders coded the data?	All data were coded by DE or CMM. A subset of approximately half of the interviews (n = 19) was double coded by a third researcher (LR). *(See page 7)*
	25	Description of the coding tree	Did authors provide a description of the coding tree?	A list of issues from the analysis of the interviews and literature search was collated into an item tracking matrix. *(See additional File)*
	26	Derivation of themes	Were themes identified in advance or derived from the data?	Issues which were conceptually similar were organised into categories. For instance, issues such as 'itchiness/irritation', 'presence of pulling sensation', and 'tightness of wound' were mapped into a 'wound comfort' category. *(See Table 2)*
	27	Software	What software, if applicable, was used to manage the data?	NVivo (version 10) was used to analyse the data (See page 6)
	28	Participant checking	Did participants provide feedback on the findings?	Full results were not sent out to all participants to gain respondent validation.
**Reporting**	29	Quotations presented	Were participant quotations presented to illustrate the themes / findings? *Was each quotation identified? e.g. participant number*	The interpretation of each category is supported by illustrative quotes *(See Table 2)*
	30	Data and findings consistent	Was there consistency between the data presented and the findings?	There is consistency between the data presented and the measures developed. The item tracking matrix provides an overview of the key findings and how these were used to develop the initial measure *(See additional file)*
	31	Clarity of major themes	Were major themes clearly presented in the findings?	The themes are clearly presented in the

For peer review only - http://bmjopen.bmj.com/site/about/guidelines.xhtml

				findings *(See pages 8-11)*
	32	Clarity of minor themes	Is there a description of diverse cases or discussion of minor themes?	Yes. Differences between the findings of the interviews and the literature search are discussed, as are the differences in satisfaction between the dressing types *(See pages 9 and 10)*

For peer review only

For peer review only - http://bmjopen.bmj.com/site/about/guidelines.xhtml

BMJ Open

Developing outcome measures assessing wound management and patient experience: A mixed methods study

Journal:	*BMJ Open*
Manuscript ID	bmjopen-2017-016155.R2
Article Type:	Research
Date Submitted by the Author:	13-Jul-2017
Complete List of Authors:	Elliott, Daisy; University of Bristol, School of Social and Community Medicine<br>Andronis, Lazaros; The University of Birmingham, Health Economics Unit, Institute of Applied Health Research<br>Blazeby, Jane; University of Bristol, Department of Social Medicine<br>Blencowe, Natalie; University of Bristol, Centre for Surgical Research, School of Social and Community Medicine<br>Calvert, Melanie; The University of Birmingham, Primary Care and General Practice<br>Coast, Joanna; University of Bristol, School of Social and Community Medicine<br>Draycott, Tim; Obstetrics & Gynaecology, North Bristol NHS Trust<br>Donovan, Jenny; University of Bristol, School of Social and Community Medicine<br>Gooberman-Hill, Rachael; University of Bristol, School of Clinical Sciences<br>Longman, Robert; University Hospitals Bristol, Dept of Coloproctology<br>Magill, Laura ; Queen Elizabeth Hospital<br>Mathers, Jonathan; University of Birmingham, Public Health<br>Pinkney, Thomas ; University of Birmingham, Centre for Patient Reported Outcomes Research; Queen Elizabeth Hospital, Academic Department of Surgery<br>Reeves, Barnaby; University of Bristol, Bristol Heart Institute<br>rogers, chris; University of Bristol, Bristol Heart Institute<br>Rooshenas, Leila; University of Bristol, School of Social and Community Medicine<br>Torrance, Andrew; The University of Birmingham, Institute of Applied Health Research<br>Welton, Nicky; University of Bristol, School of Social and Community Medicine<br>Woodward, Mark; University Hospitals Bristol<br>Young, Trudie; Welsh Wound Innovation Centre<br>Dumville, Jo C.; University of Manchester, School of Nursing, Midwifery & Social Work<br>Flintoff, Louise; University Hospitals Bristol<br>Macefield, Rhiannon; University of Bristol, Department of Social Medicine<br>Messenger, David; University Hospitals Bristol<br>McMullan, Christel; University of Birmingham, Institute of Applied Health Research<br>Murkin, Charlotte; University of Bristol, School of Social and Community Medicine

	van der Nelson, Helen; North Bristol NHS Trust, Obstetrics & Gynaecology
**Primary Subject Heading**:	Surgery
Secondary Subject Heading:	Qualitative research
Keywords:	QUALITATIVE RESEARCH, Questionnaire development, Wounds, Dressings

SCHOLARONE™
Manuscripts

For peer review only

Developing outcome measures assessing wound management and patient experience: A mixed methods study

The Bluebelle Study Group*

Corresponding author: Daisy Elliott [daisy.elliott@bristol.ac.uk]

*The Bluebelle Study Group consists of the following sub-groups:

Bluebelle grant co-applicants: Lazaros Andronis[1], Jane Blazeby[2,3], Natalie Blencowe[2,3,] Melanie Calvert[4], Joanna Coast[2,7], Tim Draycott[6], Jenny L Donovan[2,7], Rachael Gooberman-Hill[8], Robert Longman[3], Laura Magill[9], Jonathan Mathers[4], Thomas Pinkney[5], [9], Barnaby C Reeves[10], Chris A Rogers[10], Leila Rooshenas[2,] Andrew Torrance[4], Nicky J Welton[2], Mark Woodward[3], Trudie Young[11]

Other members of the Bluebelle Study Group: Jo Dumville[12], Daisy Elliott[2], Louise Flintoff[3], Rhiannon Macefield[2], David Messenger[3], Christel McMullan[4], Charlotte Murkin[2,3], Helen van der Nelson[6]

1. Health Economics Unit, Institute of Applied Health Research, University of Birmingham, Birmingham, UK.

2. School of Social and Community Medicine, University of Bristol, Bristol, UK.

3. University Hospitals Bristol NHS Foundation Trust, Bristol, UK.

4. Institute of Applied Health Research, University of Birmingham, Birmingham, UK.

5. Centre for Patient Reported Outcomes Research, University of Birmingham

6. North Bristol NHS Trust, Bristol, UK.

7. NIHR Collaboration for Leadership in Applied Health Research and Care West at University Hospitals Bristol NHS Trust, Bristol, UK

8. Musculoskeletal Research Unit, School of Clinical Sciences, University of Bristol, UK.

9. Academic Department of Surgery, Queen Elizabeth Hospital, University of Birmingham, UK.

For peer review only

10. Clinical Trials and Evaluation Unit, School of Clinical Sciences, University of Bristol, Bristol, UK.

11. Welsh Wound Innovation Centre, Rhodfa Marics, Ynysmaerdy, Pontyclun, Rhondda Cynon Taf, Wales, UK.

12. School of Nursing, Midwifery & Social Work, University of Manchester, Manchester, UK.

For peer review only

Running title: Outcome measures for wound management and patient experience

Keywords: Qualitative research; wounds; dressings; outcome measures; instrument development; patient-reported outcomes; questionnaire development; patient experience

Word count: 3035 (excluding title page, abstract, tables and references)

3

ABSTRACT

Objectives: To develop outcome measures to assess practical management of primary surgical wounds and patient experience.

Design: Mixed methods, including qualitative interviews and data extraction from published RCTs.

Setting: Two university-teaching NHS hospitals and three district NHS hospitals in the South West and Midlands regions of England.

Participants: Sixty-four patients and 15 health care professionals from abdominal general surgical specialities and obstetrics (caesarean section).

Methods: Measures were developed according to standard guidelines to identify issues relevant to patients' experiences of surgical wounds and dressings, including analysis of existing RCT outcomes and interviews. These were written into provisional questionnaire items for a single outcome measure. Cognitive interviews with patients and health care professionals assessed face validity, acceptability and relevance. Findings from interviews were regularly shared with the study team who suggested amendments to modify and reword items to improve understanding before further iterative testing with patients and health care professionals.

Results: Analyses of existing RCT outcomes and interviews produced a total of 69 issues. Pre-testing and iterative revision established the need for two separate measures. One measure addresses health care professionals' experience of wound management in two key areas: exudate and its impact, and allergic reactions to the dressing. The other measure addresses patients' experience of wounds in seven key areas: wound comfort, dressing removal, dressings to protect the wound, impact on daily activities, ease of movement, anxiety about the wound and satisfaction with dressing. Each measure took less than five minutes to complete and were understood and acceptable to patients and health care professionals.

Conclusion: This in-depth study has developed two measures to assess practical management of primary surgical wounds and patient experience. Further work to test their validity and reliability and application to other settings is now required.

STRENGTHS AND LIMITATIONS OF THIS STUDY

- This is the first study to explore the important issues related to the practical management of primary surgical wounds and patient experience immediately following surgery.

For peer review only

- This study used robust methods to identify key issues of outcome that could be used to inform decision-making around dressings. Interviews provided a rich account of the key factors that affected wound management and patient experience while a purposeful sampling strategy ensured that perspectives were captured from a range of participants. Data produced from the interviews were supplemented by analysis of existing RCT outcomes to ensure a comprehensive list of issues was considered.
- Future work is needed to test the reliability, validity and sensitivity of the new measures.

For peer review only

INTRODUCTION

An estimated 234 million major surgical procedures are undertaken worldwide every year (1). It is common practice to apply dressings over the closed wound in adult surgery and many different dressing types are available (2). A recent Cochrane systematic review summarised data relating to wound dressings and risk of surgical site infection (SSI) in primary surgical wounds. No evidence was found to suggest that any type of dressing significantly reduced the risk of developing an SSI compared with leaving wounds exposed; neither was there any benefit associated with particular dressings (3).

Decision-making around dressings may therefore need to be informed by other properties and qualities that dressings can offer, such as absorption of exudate, patient comfort, offering physical protection, facilitating wound observation, and meeting patients' desires for wound coverage (4). Whilst measures for assessment of wound cosmesis (in the longer term) are available (5, 6), there is a lack of well-developed and validated measurement tools relating to practical wound management or patient experience (4, 7, 8). Such an instrument could be used to monitor the care of individual patients (e.g. assessing the ability of dressings to manage specific symptoms), audits (e.g. quality assurance) and research (e.g. comparing patient satisfaction).

The development of patient-reported outcome measures (PROMs) increasingly includes the use of qualitative research methods which provide the opportunity to elicit and characterise patients' experiences of their health conditions and treatment (9, 10). Qualitative methods can also define health professionals' experiences of care and management (11). Data can be supplemented by expert input and studies in published literature (12, 13). This article describes the development of measures to assess practical wound management issues, symptoms and patient experience associated with primary surgical wounds.

METHODS

Study design

Measures were developed according an existing framework for developing PROMs (14, 15), also incorporating guidance on eliciting health domain concepts using qualitative methodologies (12, 13, 16). The study is reported according to qualitative reporting guidelines (See Supplementary File 1). Phase 1 aimed to produce a comprehensive list of potential issues relating to wound and dressing experience and practical management issues.

For peer review only

Phase 2 developed issues identified from Phase 1 into questionnaire items. Phase 3 evaluated the measures for acceptability and relevance using cognitive interviews with patients and health care professionals. The final part of development (phase 4) consisted of psychometric testing and will be reported elsewhere. Ethical approval was provided by the NHS Health Research Authority NRES Committee London – Camden & Kings Cross (14/LO/0640). Written informed consent was provided by all participants.

Phase 1: Generation of relevant issues

1.1 Interviews

Interviews were conducted with patients to explore and characterise experiences of wounds and dressings. Participants were recruited as part of a wider feasibility study to explore whether a trial comparing different types of dressings, and dressing versus no dressings, is possible (The Bluebelle study: a feasibility study of three wound dressing strategies in elective and unplanned surgery, HTA - 12/200/04 (2, 17)). Participants were recruited from two University-teaching NHS hospitals and three district NHS hospitals in the South West and Midlands regions of England. Eligible patients who had undergone, or were scheduled to undergo, an abdominal surgical procedure or caesarean section were identified and approached by research nurses and surgical trainees. The qualitative team contacted interested patients to arrange interviews. A purposeful sampling strategy ensured that perspectives were captured from a range of participants (13). Within this sampling approach, maximum variation was sought in relation to age, gender, ethnicity, type of surgery, dressing type and location. A topic guide was developed (based on literature and views of health care professionals in the Bluebelle study team) to ensure that discussions covered the same core issues but with sufficient flexibility to allow new issues of importance to the informants to emerge (See Supplementary File 2).

Interviews were audio-recorded and transcribed in full. Transcripts were imported into NVivo (version 10). All data relating to outcomes and issues of importance to patients that were relevant to dressing use and the practical management of the wound in the initial time period post-surgery were assigned labels (coded) by two experienced qualitative researchers. Data were analysed using techniques of constant comparison derived from grounded theory methodology, and emerging codes across the dataset were then compared to look for shared or disparate views among participants (18). A subset of approximately half of the interviews (n = 19) was double coded by third experienced researcher to highlight any differences in

For peer review only - http://bmjopen.bmj.com/site/about/guidelines.xhtml

the interpretation of codes (12). Data collection and analysis continued until the team were confident that saturation had been reached i.e. no more patterns or themes emerged from the data (19).

1.2 Extraction of information from three systematic reviews

Systematic reviews were purposefully selected to identify RCTs measuring outcomes relating to patient experience and management of wound healing. Since the wider Bluebelle study was exploring whether a trial comparing different types of dressings (including dressings versus the novel use of tissue glue as a dressing) to no dressing was possible, we selected three recent systematic reviews (4, 20, 21) to identify RCTs which included outcomes relevant to both dressings and the use of tissue adhesive. Although not published at the time of conducting this work, additional references from an updated version of one of the systematic reviews (7) were also provided. Published papers reporting the RCTs included in the systematic reviews were obtained where possible. Relevant data from the RCT reports were then extracted on the outcome (as described by the authors), the verbatim wording to measure outcome, who reported the outcome, the measurement scale and the assessment time point. Attempts were made to contact the authors for more information.

1.3 Synthesis of findings from interviews and data extraction

A list of issues from the analysis of the interviews and analysis of existing RCT outcomes was collated into an item tracking matrix, in line with guidance for developing PROMs (22). This is available in the online appendix (see Supplementary File 3). The study team agreed on a set of words or phrases to reflect each issue and also noted additional phrasing made by participants in a subsequent column (12). Issues which were conceptually similar were organised into categories. For instance, issues such as ‘itchiness/irritation’, ‘presence of pulling sensation’, and ‘tightness of wound’ were mapped into a ‘wound comfort’ category.

Phase 2: ‘Operationalisation’: Construction of a provisional measure

The item tracking matrix was used to determine which issues should be written into questionnaire items. Items featured words and phrases used by patients in the interviews to enhance content validity (13, 23).

Phase 3: Pre-testing

For peer review only - http://bmjopen.bmj.com/site/about/guidelines.xhtml

Participants were recruited from two University-teaching NHS hospitals in the South West and Midlands regions of England. Patients who had undergone abdominal general surgery or caesarean section and health care professionals involved in post-surgical care were approached. As in Phase 1, sampling was purposeful to achieve maximum variation in relation to clinical role, age, gender and geographic location (for health care professionals) and age, gender, ethnicity, type of surgery, dressing type and location (for patients).

Cognitive interviews are used widely in questionnaire development (12) and involve asking respondents to verbalise their thoughts while answering questions (24). This methodology enabled us to explore the acceptability of the measure and coverage of patients and health care professionals' concerns (in terms of language, accuracy, and relevance, as well as layout (13)). During each interview, participants were asked to complete the measure by reading each item aloud and commenting on their understanding. Interviews were guided by a series of probes (e.g. 'What does this item mean to you?', 'Are there other ways you would describe it?'; (24)). Participants' body language (such as nodding or frowning) was also observed and prompted further discussion about specific items (12). A copy of the topic guide is available (See Supplementary File 2).

The qualitative team maintained detailed field notes from each interview, describing suggestions for modifications and improvements to the provisional measures. Operationalisation and modification of the measures was an iterative process. Findings from the interviews and suggestions for amendments were regularly disseminated to the Bluebelle Study Group, which consisted of a multidisciplinary group of health care professionals, including surgeons, health services researchers and research nurses. Each stage of feedback informed amendments to modify and reword items to improve understanding, which was repeated following efforts to revise questions and eliminate problems (24). This process continued until no new issues were identified and no further refinements were believed to be necessary.

RESULTS

Phase 1: Generation of relevant issues

1.1 Interviews

A total of 39 interviews were conducted between July 2014 and July 2015. Interviews were conducted in person (n=10), unless patients preferred to be interviewed via telephone

For peer review only

(n=29). Interviews lasted an average of 25 minutes (range = 15–50 minutes). The sample consisted of 27 women and 12 men, who mostly described themselves as white British (90%). They had a mean age of 56 years (range 22-88 years). Thirty-seven of the 39 participants had either undergone abdominal general surgery (85%) or a caesarean section (15%), with an average of 18 days since their surgery (range =6-40 days). Two of the 39 patients were scheduled to undergo abdominal general surgery and discussed issues that they anticipated would be important to them. Participant demographics for Phase 1 interviews are shown in Table 1.

Table 1: Demographics of participants' interviews in Phases 1 and 3

		**Phase 1: Generation of relevant issues**	**Phase 3: Pre-testing**	
**Qualitative interviews (n = 79)**		39 patients	25 patients	15 health care professionals
**Age (years)**	Range	22-88	19-76	23-60
	Mean	56	54	41
**Sex**	Female	27	12	13
	Male	12	13	2
**Ethnicity**	White	35	22	14
	Asian	1	1	0
	African	2	1	1
	Indian	1	0	0
	Filipino	0	1	0
**Type of surgery**	Abdominal	33	25	15
	Obstetric	6	0	0
**Dressing type**	Tissue adhesive	7	5	-
	Adhesive	32	18	-
	No dressing	0	2	-
**Location**	Bristol	28	15	9
	Birmingham	11	10	6

1.2 Extraction of information from three systematic reviews

Published papers for 26 studies that included outcomes relating to patient experience and management of wound healing were identified from the three systematic reviews (25-50). Only two studies included a validated instrument, or modification of a validated instrument, to assess outcomes (27, 41). These were for long term scarring and cosmesis (5, 6). However, no studies reported using validated measures relating to issues associated with practical wound management and patient experiences in the early post-operative period. Descriptions of outcomes were heterogeneous and often poorly defined. The most common reported outcomes related broadly to cosmetic result (reported in 15/26 studies), dressing changes (e.g. frequency, comfort, ease of application and removal; reported in 11/26

For peer review only

studies), and skin reactions (e.g. itching, blistering; reported in 10/26 studies). Full data extraction from the 26 studies is included in Supplementary File 4.

1.3 Synthesis of findings from interviews and data extraction

When describing experiences in the interviews, patients commented on several factors that affected perceptions of how well their wound was healing, including how it felt (tightness, pain, and itchiness) and whether any fluid had leaked from the wound. Analysis of existing RCT outcomes showed these issues had been captured in some previous (unvalidated) outcomes.

All patients had at least one dressing applied after surgery, although this varied between adhesive coverings (absorptive or non-absorptive) and tissue adhesive as a dressing. Both the interviews and the analysis of existing RCT outcomes highlighted that multiple practical advantages of dressing use (including ability to contain exudate and ease of removal). The interviews also demonstrated that there were psychological factors that affected dressing experience and satisfaction (i.e. anxiety about cleanliness of the wound). Patients with tissue adhesive as a dressing commented that they had been surprised that their wounds had been dressed this way (rather than adhesive dressings which they had had in the past for other wounds). However, these patients stated that compared to past experiences of adhesive dressings, they liked how glue was transparent, waterproof, did not require multiple applications and came off naturally.

The interviews and the analysis of existing RCT outcomes produced a total of 69 issues. These were grouped into ten broad categories: wound comfort, exudate and its impact, allergic reactions to the dressing, dressing removal, dressings to protect the wound, impact on daily activities, ease of movement, anxiety about the wound, satisfaction with dressing and wound appearance. Table 2 provides illustrative quotes for the categories identified.

Table 2: Categories identified

Category	Example quote
**Wound comfort**	*"I've now got really itchy where the plaster goes. Which is uncomfortable."* (Patient, adhesive dressing)
**Exudate and its impact**	*"If I walked around it would get really damp. I mean it would soak my pyjamas and drip down my legs. It was quite manky really…Then they would put a sort of big, well, like a big plaster on top of that, and then they put a kind of absorbent pad over that, to absorb some of that liquid."* (Patient, adhesive dressing)
**Reactions to the dressing**	*"I was allergic to the surgical tape."* (Patient, adhesive dressing)

For peer review only

11

**Dressing removal**	*"I just completely soaked it [adhesive dressing] in the shower then my husband just took it off for me. But it was, it was really easy. Much easier than I thought."* (Patient, adhesive dressing)
**Wound protection**	*"I'd be worried about catching it [the wound], knocking it, or something getting in so that it became infected."* (Patient, adhesive dressing)
**Impact on daily activities**	*"With the glue [dressing] it's easy to shower. With a [adhesive] dressing it wouldn't be so easy to shower and you'd be worried."* (Patient, tissue adhesive dressing)
**Ease of movement**	*"What I do find is the dressings are a bit constricting, especially as I get a bit better because they don't turn with your body so easily and then I feel that it makes me feel more constricted."* (Patient, tissue adhesive dressing)
**Anxiety about the wound**	*"You could catch things just from the air. That made me think, "Well, you'd need something to kind of protect it."* (Patient, adhesive dressing)
**Satisfaction with dressing**	*"Glue [as a dressing] requires no maintenance. I was very pleased. You don't have to change it you just leave it alone … I think that helps with the healing process physically and mentally."* (Patient, tissue adhesive dressing)
**Wound appearance**	*"If it was red and inflamed I would have thought, "Something has gone wrong with it."* (Patient, adhesive dressing)

Phase 2: 'Operationalisation': Construction of a provisional measure

A provisional measure was designed based on the findings from Phase 1. Nine key categories were included: wound comfort, exudate and its impact, allergic reactions to the dressing, dressing removal, dressings to protect the wound, impact on daily activities, ease of movement, anxiety about the wound and satisfaction with dressing. Issues relating to the appearance of the wound were not included as they were only relevant to longer term outcomes of wound healing (not within first days of surgery). Additionally, since most patients reported having an adhesive dressing, many had not seen their wound within this timeframe. The first version of the measure included 16 items, and was provisionally called the Practical Wound Management Questionnaire.

Phase 3: Pre-testing

Cognitive interviews (n = 40) were conducted between July 2015 and March 2016. All interviews were conducted face-to-face. This consisted of 25 patients who were in hospital and had undergone abdominal general surgery, and 15 health care professionals involved in surgical wound care. Demographics are shown in Table 1.

Interviews highlighted issues with content in the initial measure. For example, items regarding the colour of the wound exudate were removed. Questions were rephrased to focus on the experience of having a dressing rather than general recovery after surgery (i.e. '*Have you been able to perform everyday tasks? (i.e. showering/bathing)*' was changed to '*Has your dressing prevented you from showering/washing?*'). Additionally, since four patients

For peer review only

commented that the smell of their wound was missing on the measure, an item was added to capture this.

The measure had intended to be administered two days after surgery, although feedback suggested that this needed to be completed up to day four as the patient may be disorientated from surgery in the first few days. However, since there were clear differences in recovery with caesarean section and abdominal surgery patients, a timeframe of within four days of surgery was set, and the measures recorded the date of surgery and date completed to determine context of responses.

Feedback from patients suggested it was difficult to respond to questions about exudate, since a health care professional cared for their wound whilst they were in hospital. If their dressing had been changed, they were also uncertain about the reason why (i.e. simply as part of standard practice or for other reasons). Therefore, the study team decided to separate the measure into two separate measures. The first related to the practical aspects of wound management and the second related to the patient's experience of the wound/dressing and the psychological aspects (anxiety, satisfaction etc). The two measures were named the Wound Management Questionnaire and the Wound Experience Questionnaire.

Seven versions of measures were modified throughout the pre-testing phase. Pre-testing continued until no new issues were identified and no further refinements were believed to be necessary. The final version of The Wound Management Questionnaire contains four items, whilst The Wound Experience Questionnaire contains 10 items (See Supplementary File 5). Overall, the final versions of the measures were well received. In addition, 96% of participants stated that each measure took less than five minutes to complete.

DISCUSSION

This paper describes the development of two measures for assessing wound management and experience. The Wound Management Questionnaire assesses practical issues early after surgery for completion by health care professionals and The Wound Experience Questionnaire assesses symptoms and patient satisfaction with their wound and dressings. These measures were developed using a mixed methods approach, including data extraction from 26 published RCTs and interviews with 64 patients and 15 health care professionals. Final versions of the measures were easily completed and acceptable to patients and health

care professionals. Further work is needed to examine their reliability and validity in a wider group of patients.

Given the absence of evidence supporting the effectiveness of dressings for the prevention of SSIs, decision-making around dressings needs to be informed by issues such as managing wound exudate, offering physical protection and meeting patients' desires for wound coverage. However, systematic reviews have highlighted a lack of meaningful outcome data on wound symptom management and patient experiences of primary surgical wounds and acceptability of dressings (4, 7, 8). To our knowledge, this is the first study to explore these important issues in patients with closed primary surgical wounds. The measures are intended to be used in future studies including a wide variety of primary abdominal wounds such as those created during elective or acute surgery, surgery for benign or malignant disease and bowel resection of obstetric procedures. Such studies will always record the patient group which will be important to consider when looking at the results of the measures.

True patient-centred outcome measures require full consideration of patients' experiences and views' (9, 10). The main strength of this research, therefore, is the use of qualitative research methods to provide important insights into the under researched area of early issues related primary surgical wounds relating to practical wound management and patient experience. We adopted a purposeful sampling strategy to ensure that perspectives were captured from a range of participants in relation to their primary surgical abdominal wound (12). Data produced from the interviews were supplemented by an analysis of existing RCT outcomes to ensure a comprehensive list of issues were initially generated, and therefore acted as a method of triangulation to increase the plausibility and dependability of the interview data (13).

However, it is important to note that these measures have only been pre-tested in relation to primary surgical wounds. Wounds that are intentionally left open or wounds that have developed problems are likely to require dressings that have advanced practical properties that are tailored to the wound requirements (2). Although participants were purposefully sampled, most had had a dressing of some kind (94%). A prospective real-time survey of dressings has demonstrated that this reflects current practice (17). In addition, these measures have only been pre-tested in relation to abdominal surgical wounds. However, characteristics of wound healing in this area are likely to be consistent with many other parts of the body. Furthermore, these measures focus specifically on the experience of dressings - methods of wound closure

For peer review only

(i.e. potentially leading to differential ease of removal of sutures or staples) may also affect patient experience, although this would require further investigation.

In summary, our measures can be completed both by patients and by health care professionals responsible for post-operative wound care. These measures will now be further developed to ensure they are appropriate and psychometrically-tested instruments, with a view to informing decision-making around dressings.

ACKNOWLEDGEMENTS

We would like to thank all participants for giving up their time to take part in this study, and members of the Bluebelle study team for their helpful comments on the earlier drafts of the measures.

CONTRUBITIONS

All members of the Bluebelle Study Group read and commented on the final version of the paper. Other roles are described below.

Lazaros Andronis co-applicant in the Bluebelle study grant (Health Economics); **Jane Blazeby** Chief investigator of the Bluebelle study grant (NIHR Bluebelle grant HTA - 12/200/04), led the whole study including chairing of management and executive meetings, supervised development of the questionnaires; **Natalie Blencowe** co-applicant in the Bluebelle study grant, provided feedback on earlier versions of the questionnaires and recruited patients/health care professionals for interviews; **Melanie Calvert** co-applicant in the Bluebelle study grant (patient-reported outcomes expertise); **Joanna Coast** co-applicant in the Bluebelle study grant (Health Economics lead); **Jenny L Donovan** co-applicant in the Bluebelle study grant (Qualitative research co-lead), co-designed and supervised the qualitative research; **Tim Draycott** co-applicant in the Bluebelle study grant (Obstetric surgical expertise and leadership) principal investigator and lead advisor for obstetric surgery; **Jo Dumville** provided feedback on earlier versions of the questionnaires; **Daisy Elliott** wrote the first draft of the manuscript and edited/finalised the paper, contributed to all aspects of qualitative data collection and qualitative data analysis and led development of the questionnaires; **Louise Flintoff** recruited patients/health care professionals for interviews; **Rachel Gooberman-Hill** co-applicant in the Bluebelle study grant (patient and public

For peer review only

involvement lead); **Robert Longman** co-applicant in the Bluebelle study grant (Surgical expertise and leadership); **Rhiannon Macefield** conducted literature reviews and provided feedback on earlier versions of the questionnaires; **Laura Magill** co-applicant in the Bluebelle study grant, trial manager in Birmingham; **Jonathan Mathers** co-applicant in the Bluebelle study grant (Qualitative research co-lead); **Christel McMullan** contributed to all aspects of qualitative data collection and qualitative data analysis, provided feedback on earlier versions of the questionnaires; **David Messenger** recruited patients/health care professionals for interviews; **Charlotte Murkin** conducted pre-testing interviews; **Helen van der Nelson** recruited patients/health care professionals for interviews; **Tom Pinkney** co-applicant in the Bluebelle study grant (Surgical expertise and leadership); **Barnaby C Reeves** co-applicant in the Bluebelle study grant (Methodological lead), contributed to overall study design, and provided feedback on earlier versions of the questionnaires; **Chris A Rogers** co-applicant in the Bluebelle study grant (Statistical lead); **Leila Rooshenas** contributed to all aspects of qualitative data collection and qualitative data analysis in Phase I and provided feedback on earlier versions of the questionnaires; **Andrew Torrance** co-applicant in the Bluebelle study grant; **Nicky J Welton** co-applicant in the Bluebelle study extension grant; **Mark Woodward** co-applicant in the Bluebelle study grant (Paediatric surgical expertise and leadership) and advisor on paediatric surgery; **Trudie Young** co-applicant in the Bluebelle study grant (Wound nursing specialist) and advisor on wound care.

COMPETING INTERESTS

None declared.

DISCLAIMER

The views expressed are those of the authors and not necessarily those of the MRC, NHS, NIHR or the Department of Health.

FUNDING

The Bluebelle study (Phase A) is funded by the National Institute for Health Research (NIHR) Health Technology Assessment (HTA) Programme (HTA - 12/200/04). JLD and JMB are NIHR Senior Investigators. JLD is also supported by the NIHR Collaboration for Leadership in Applied Health Research and Care (CLAHRC) West at University Hospitals Bristol NHS Foundation Trust. The Bluebelle study was undertaken with the support of the MRC

ConDuCT-II Hub (Collaboration and innovation for Difficult and Complex randomised controlled Trials In Invasive procedures - MR/K025643/1).

ETHICAL APPROVAL

Ethical approval for this work was granted by the Camden and King's Cross Research Ethics Committee (14/LR/0640) on the 10th April 2014.

DATA SHARING STATEMENT

No additional data are available.

REFERENCES

1. Weiser TG, Regenbogen SE, Thompson KD, Haynes AB, Lipsitz SR, Berry WR, et al. An estimation of the global volume of surgery: a modelling strategy based on available data. The Lancet.372(9633):139-44.
2. Andronis L, Blazeby J, Blencowe N, Calvert M, Coast J, Draycott T, et al. Bluebelle study (phase A): a mixed-methods feasibility study to inform an RCT of surgical wound dressing strategies. BMJ open. 2016;6(9):e012635.
3. Dumville JC, Gray TA, Walter CJ, Sharp CA, Page T, Macefield R, et al. Dressings for the prevention of surgical site infection. Cochrane Database of Systematic Reviews. 2016(12).
4. Dumville JC, Coulthard P, Worthington HV, Riley P, Patel N, Darcey J, et al. Tissue adhesives for closure of surgical incisions. The Cochrane Library. 2014.
5. Sullivan Ta, Smith J, Kermode J, Mclver E, Courtemanche D. Rating the burn scar. Journal of Burn Care & Research. 1990;11(3):256-60.
6. Hollander JE, Singer AJ, Valentine S, Henry MC. Wound registry: development and validation. Ann Emerg Med. 1995;25(5):675-84.
7. Dumville JC, Gray TA, Walter CJ, Sharp CA, Page T. Dressings for the prevention of surgical site infection. The Cochrane Library. 2014.
8. Walter C, Dumville J, Sharp C, Page T. Systematic review and meta-analysis of wound dressings in the prevention of surgical-site infections in surgical wounds healing by primary intention. Br J Surg. 2012;99(9):1185-94.
9. Wilburn J, McKenna SP, Twiss J, Kemp K, Campbell S. Assessing quality of life in Crohn's disease: development and validation of the Crohn's Life Impact Questionnaire (CLIQ). Qual Life Res. 2015;24(9):2279-88.
10. Bausewein C, Simon ST, Benalia H, Downing J, Mwangi-Powell FN, Daveson BA, et al. Implementing patient reported outcome measures (PROMs) in palliative care-users' cry for help. Health and quality of life outcomes. 2011;9(1):27.
11. Trujols J, Portella MJ, Iraurgi I, Campins MJ, Siñol N, Cobos JPdL. Patient-reported outcome measures: Are they patient-generated, patient-centred or patient-valued? Journal of Mental Health. 2013;22(6):555-62.
12. Patrick DL, Burke LB, Gwaltney CJ, Leidy NK, Martin ML, Molsen E, et al. Content Validity—Establishing and Reporting the Evidence in Newly Developed Patient-Reported Outcomes (PRO) Instruments for Medical Product Evaluation: ISPOR PRO Good Research Practices Task Force Report: Part 1—Eliciting Concepts for a New PRO Instrument. Value Health. 2011;14(8):967-77.
13. Patrick DL, Burke LB, Gwaltney CJ, Leidy NK, Martin ML, Molsen E, et al. Content Validity—Establishing and Reporting the Evidence in Newly Developed Patient-Reported Outcomes (PRO)

For peer review only

Instruments for Medical Product Evaluation: ISPOR PRO Good Research Practices Task Force Report: Part 2—Assessing Respondent Understanding. Value Health. 2011;14(8):978-88.
14. Sprangers M, Cull A, Bjordal K, Groenvold M, Aaronson NK. The European Organization for Research and Treatment of Cancer approach to quality of life assessment: guidelines for developing questionnaire modules. Qual Life Res. 1993;2(4):287-95.
15. Sprangers M, Cull A, Groenvold M, Bjordal K, Blazeby J, Aaronson NK. The European Organization for Research and Treatment of Cancer approach to developing questionnaire modules: an update and overview. Qual Life Res. 1998;7(4):291-300.
16. Lasch KE, Marquis P, Vigneux M, Abetz L, Arnould B, Bayliss M, et al. PRO development: rigorous qualitative research as the crucial foundation. Qual Life Res. 2010;19(8):1087-96.
17. Andronis L, Calvert M, Magill L, Mathers J, Pinkney T, Torrance A, et al. Feasibility work to inform the design of a randomized clinical trial of wound dressings in elective and unplanned abdominal surgery. The British Journal of Surgery. 2016;103(12):1738.
18. Glaser BG, Strauss AL. The Discovery of Grounded Theory. Aldine Transaction: USA; 1967.
19. Braun V, Clarke V. Using thematic analysis in psychology. Qualitative Research in Psychology. 2006;3(2):77-101.
20. Dumville JC, Walter CJ, Sharp CA, Page T. Dressings for the prevention of surgical site infection. Cochrane Database of Systematic Reviews. 2011(7).
21. Chow A, Marshall H, Zacharakis E, Paraskeva P, Purkayastha S. Use of tissue glue for surgical incision closure: a systematic review and meta-analysis of randomized controlled trials. J Am Coll Surg. 2010;211(1):114-25.
22. Health UDo, Evaluation HSFCfD, Research, Health UDo, Evaluation HSFCfB, Research, et al. Guidance for industry: patient-reported outcome measures: use in medical product development to support labeling claims: draft guidance. Health and Quality of Life Outcomes. 2006;4:1-20.
23. Leidy NK, Vernon M. Perspectives on patient-reported outcomes. Pharmacoeconomics. 2008;26(5):363-70.
24. Beatty PC, Willis GB. Research Synthesis: The Practice of Cognitive Interviewing. Public Opin Q. 2007;71(2):287-311.
25. Amin M, Glynn F, Timon C. Randomized trial of tissue adhesive vs staples in thyroidectomy integrating patient satisfaction and Manchester score. Otolaryngology--Head and Neck Surgery. 2009;140(5):703-8.
26. Avşar AF, Üstüner I, Keskin HL, Öztürk Ö, Taş EE. 2-octylcyanoacrylate tissue adhesive versus polypropylene suture for skin closure of Pfannenstiel incision. J Turk Soc Obstet Gynecol. 2009;6:117-22.
27. Blondeel PN, Murphy JW, Debrosse D, Nix JC, Puls LE, Theodore N, et al. Closure of long surgical incisions with a new formulation of 2-octylcyanoacrylate tissue adhesive versus commercially available methods. The American journal of surgery. 2004;188(3):307-13.
28. Burke NG, Green C, McHugh G, McGolderick N, Kilcoyne C, Kenny P. A prospective randomised study comparing the jubilee dressing method to a standard adhesive dressing for total hip and knee replacements. Journal of tissue viability. 2012;21(3):84-7.
29. Chibbaro S, Tacconi L. Use of skin glue versus traditional wound closure methods in brain surgery: A prospective, randomized, controlled study. J Clin Neurosci. 2009;16(4):535-9.
30. Cosker T, Elsayed S, Gupta S, Mendonca A, Tayton K. Choice of dressing has a major impact on blistering and healing outcomes in orthopaedic patients. J Wound Care. 2005;14(1):27-9.
31. Dowson CC, Gilliam AD, Speake WJ, Lobo DN, Beckingham IJ. A prospective, randomized controlled trial comparing n-butyl cyanoacrylate tissue adhesive (LiquiBand) with sutures for skin closure after laparoscopic general surgical procedures. Surgical Laparoscopy Endoscopy & Percutaneous Techniques. 2006;16(3):146-50.
32. Gennari R, Rotmensz N, Ballardini B, Scevola S, Perego E, Zanini V, et al. A prospective, randomized, controlled clinical trial of tissue adhesive (2-octylcyanoacrylate) versus standard wound closure in breast surgery. Surgery. 2004;136(3):593-9.

For peer review only

18

33. Greene D, Koch RJ, Goode RL. Efficacy of octyl-2-cyanoacrylate tissue glue in blepharoplasty: a prospective controlled study of wound-healing characteristics. Arch Facial Plast Surg. 1999;1(4):292-6.
34. Holm C, Petersen JS, Grønbœk F, Gottrup F. Effects of occlusive and conventional gauze dressings on incisional healing after abdominal operations. Eur J Surg. 1998;164(3):179-83.
35. Kent A, Liversedge N, Dobbins B, McWhinnie D, Jan H. A prospective, randomized, controlled, double-masked, multi-center clinical trial of medical adhesives for the closure of laparoscopic incisions. J Minim Invasive Gynecol. 2014;21(2):252-8.
36. Keng TM, Bucknall TE. A clinical trial of tissue adhesive (histoacryl) in skin closure of groin wounds. The Medical journal of Malaysia. 1989;44(2):122-8.
37. Khan R, Fick D, Yao F, Tang K, Hurworth M, Nivbrant B, et al. A comparison of three methods of wound closure following arthroplasty. Bone & Joint Journal. 2006;88(2):238-42.
38. Law N, Ellis H. Exposure of the wound-a safe economy in the NHS. Postgrad Med J. 1987;63(735):27-8.
39. Lawrentschuk N, Falkenberg MP, Pirpiris M. Wound blisters post hip surgery: A prospective trial comparingdressings. ANZ J Surg. 2002;72(10):716-9.
40. Livesey C, Wylde V, Descamps S, Estela C, Bannister G, Learmonth I, et al. Skin closure after total hip replacement. Bone & Joint Journal. 2009;91(6):725-9.
41. Michie DD, Hugill JV. Influence of occlusive and impregnated gauze dressings on incisional healing: a prospective, randomized, controlled study. Ann Plast Surg. 1994;32(1):57-64.
42. Ong C, Jacobsen A, Joseph V. Comparing wound closure using tissue glue versus subcuticular suture for pediatric surgical incisions: a prospective, randomised trial. Pediatr Surg Int. 2002;18(5-6):553-5.
43. Pronio A, Di Filippo A, Narilli P, Caporillli D, Vestri A, Ciamberlano B, et al. Closure of cutaneous incision after thyroid surgery: a comparison between metal clips and cutaneous octyl-2-cyanoacrylate adhesive. A prospective randomized clinical trial. Eur J Plast Surg. 2011;34(2):103-10.
44. Ravnskog FA, Espehaug B, Indrekvam K. Randomised clinical trial comparing Hydrofiber and alginate dressings post-hip replacement. J Wound Care. 2011;20(3):136-42.
45. Romero P, Frongia G, Wingerter S, Holland-Cunz S. Prospective, randomized, controlled trial comparing a tissue adhesive (Dermabond™) with adhesive strips (Steri-Strips™) for the closure of laparoscopic trocar wounds in children. Eur J Pediatr Surg. 2011;21(03):159-62.
46. Shamiyeh A, Schrenk P, Stelzer T, Wayand WU. Prospective Randomized Blind Controlled Trial Comparing Sutures, Tape, and Octylcyanoacrylate Tissue Adhesive for Skin Closure After Phlebectomy. Dermatol Surg. 2001;27(10):877-80.
47. Sniezek PJ, Walling HW, Debloom JR, Messingham MJ, Vanbeek MJ, Kreiter CD, et al. A Randomized Controlled Trial of High-Viscosity 2-Octyl Cyanoacrylate Tissue Adhesive versus Sutures in Repairing Facial Wounds Following Mohs Micrographic Surgery. Dermatol Surg. 2007;33(8):966-71.
48. Vogt KC, Uhlyarik M, Schroeder TV. Moist wound healing compared with standard care of treatment of primary closed vascular surgical wounds: A prospective randomized controlled study. Wound Repair Regen. 2007;15(5):624-7.
49. Wikblad K, Anderson B. A comparison of three wound dressings in patients undergoing heart surgery. Nurs Res. 1995;44(5):312.
50. Wynne R, Botti M, Stedman H, Holsworth L, Harinos M, Flavell O, et al. Effect of three wound dressings on infection, healing comfort, and cost in patients with sternotomy wounds: a randomized trial. CHEST Journal. 2004;125(1):43-9.

For peer review only

Interview Topic Guide: Phase 1: Generation of relevant issues

Background, interviewee details and ice breaker

- Interviewee background and details of procedure that the interviewee has had (type of procedure elective or emergency? have you had before?, when, where, length of stay in hospital, recovery period).
    - *Just to give me some background, can you just tell me a little bit about the procedure that you had, the surgical procedure?*
    - *How have you been, in terms of recovery? Length of stay? When came home?*
    - *Is this your first?*

Expectations/experiences of wound care

- *Did/do you have any expectations about whether or not you would have a dressing? Where do you think these expectations came from?*
- *What kind of dressing? When did they take it off? [home/hospital?] Same one? How did you find having the dressing? Why do you think you had/did not have a dressing? Do you think it had any impact on your wound healing?*
- *Why do you think they do/don't use dressings?*
- *Thoughts/reactions to **alternative** wound management methods (i.e. dressing or no dressing in relation to what patient has experienced/what patient expects). (Explore patient thoughts on impact this may have on previously mentioned issues (e.g. recovery, symptoms, practicalities).*

Patient perspectives on a trial of dressing type

Okay, the basis of this study is like I said, we're trying to find out if dressing the wounds is helpful or not. In a study, patients would be randomly allocated by chance to either receive dressings or receive no dressings. The doctor wouldn't decide and the patient wouldn't decide. What we're trying to find out is, in patients who have had a similar type of surgery, if we have one group of patients that have them on so and another group of patients that don't, then can we compare them to see if there's any difference between the groups.

- *Do you think you would participate? (Reasons why/why not? Any reservations?)*
- *Can you imagine your family and/or friends would participate in a study like this?*
- *What kinds of things do you think you might consider, when deciding whether or not to take part? Do you think you would have any questions about the study? What kind of things might you want to know about in advance?*
- *How do you think you would feel about random allocation to dressing type, specifically the possibility of receiving no wound dressing? [explain randomisation to patients, then ask…] If you were in a group that didn't receive a dressing, do you think you would have thought about your care differently?*
- *In paediatrics, they don't use dressings. Changes impression?*
- *What about a glue dressing?*
- *Can you think of any potential problems we might come across if we were doing this study, any practical problems or any difficulties we might come across?*
- *Perspectives on important outcomes to include in a trial of dressing use. We would like this study to help us answer the question of whether dressings should be used in patients such as yourself (and if so, what type of dressing is best). What do you think are the important factors we should consider when making any future recommendations on dressing use? Satisfaction? Infection? Healing times?*

Closing

- Summarise key points
- Any further questions?
- Thank patient for their time and explain how they have helped.

For peer review only

Interview Topic Guide: Phase 3 – Pre-testing

Introduction

- As you know, we are going to audio-record you whilst you complete the questionnaire that we have developed. During this, we would like to understand your opinion of the questions, in terms of the language used and the relevance to your post-operative experience. It is important to remember that there are no right or wrong answers, but that this will help us to understand how the questionnaire should be developed. This should take no longer than an hour, although you can stop the discussion at any time. The information that you provide is not going to be fed back to your clinical team.
- Complete consent form and data collection form
- Any questions?

Background (procedure and recovery)

- Could you tell me a little bit about what you had done? *(Probe: When? Where? Elective/emergency?)*
- How has recovery been for you so far? *(Probe: How feeling? Length of stay? When expected to go home?)*
- How has the wound been healing? *(Probe: How does wound feel? Any problems?)*

PWMQ: Can we work through the questionnaire as you complete it, with you explaining how you understand the items and what you will put for this question.

- DT to not clarify meaning, but just repeat question.
- If P uncertain, try to understand why.
- Note any potential issues to be discussed after completion.

PWMQ: Understanding responses to each question

- Can you tell me in your own words what that question was asking? *(Probe: What does the word XX mean to you? Are there any other ways you would describe it?)*
- What does [not at all/a little/quite a bit/very much] mean to you?
- Was it easy to choose an answer?
- Explore where participants have indicated confusion

After completion:

- What did you think of the questionnaire?
- Were the questions relevant to your experience? *(Probe: Are there any others that should be included?)*
- Any difficulties filling in questions?
- Do you think it captures your experience of your wound in the past 24 hours?
- What does the word "practical wound management" mean to you?
- Do you have any other suggestions on how the questionnaire could be improved? *(Probe: alternative wording, additional questions, response categories, layout/presentation, length of questionnaire?)*
- You have given your answers based on the past 24 hours. Would your answers differ if you were thinking about your entire recovery from surgery? *(Probe: When do you think would be the best time to complete questionnaires like these?)*

Closing

- Summarise key points
- Any further questions?
- Thank patient for their time and explain how they have helped.

For peer review only

Table 1: Item tracking matrix of all issues identified

Category	Issue	Identified by: Interviews DE (n=28)	Identified by: Interviews LR (n=19)	Identified by: Interviews CM (n=11)	Identified by: Literature (n=26 RCTs)	Additional comments	Included in questionnaire? If yes, questionnaire item	Included in questionnaire? Why not included
Wound comfort	Itchiness	✓	✓	✓	✓	CM: Described by patients as an outcome (looking at the wound to check if it is irritated, inflamed, etc)<br>DE: Similar to inflammation ('burning'	Q1: Has the wound been itchy?	
	Pain	✓	✓	✓	✓	DE: Described as 'sore', 'hurt', 'tender', 'uncomfortable'.<br>DE: Sometimes mentioned in the context of a numerical scale.<br>RM: Described as 'burning pain referring to a dressing-related sensation felt under the dressing' in one study<br>RM: Also 'tenderness'<br>CM: 'sore', 'painful' – discussed in terms of dressing removal	Q2: Has the wound been painful?	
	Presence of pulling sensation	✓			✓	LR: Described as wound 'being able to breathe', 'stuffiness'	Q3: Has the wound had a pulling sensation?	
	Tightness of wound	✓	✓		✓		Q4: Has the wound felt tight?	
	Wound comfort (overall or unspecified)				✓	RM: Also measured as 'Discomfort' in the literature<br>RM: Includes 'Discomfort with skin problems'		Excluded as covered in Q1 – Q4
Exudate and its impact	Whether there was any exudate	✓	✓	✓	✓		Q5: Has the wound leaked?	
	Type of exudate (blood, other)	✓	✓	✓	✓	DE: Described as 'mess', 'manky', 'leaking', 'gunge', 'oozing', 'soaking', 'brown mess'<br>LR: 'moistness', Ooziness', 'dampness'<br>RM: Described as 'discharge' 'fluid' 'oozing'<br>CM: 'Seeping'	Q5: If so, was it: clear fluid? cloudy fluid? Blood-stained fluid? thick and yellow/green fluid?	
	Whether exudate marks bedding/clothing	✓	✓	✓		DE: Described as 'stains', LR: 'manked up clothing'	Q6: Has the leakage resulted in changed bedding/ clothes?	
	Degree dressing absorbs exudate	✓	✓	✓	✓		Q7: How would you describe the wettest dressing?	
	Whether additional dressing required	✓	✓	✓	✓	RM: Includes reasons for dressing changes<br>CM: use two dressings when oozing is important, don't want to take original dressing off	Q8: Has a dressing or glue been put on the wound (or replaced)?	
	Anxiety associated with exudate	✓	✓	✓				Excluded as captured in Q15: "Have you felt any anxiety about your wound?"
Allergic reactions to the dressing	Any allergic reactions to dressing/blistering	✓	✓	✓	✓	RM: Also include skin damage/injury	b) has the wound blistered?	
Dressing removal	Whether dressing comes off	✓	✓	✓			Q9: Has the dressing or glue come off or been removed	
	Whether dressing needs to be taken off	✓	✓	✓		CM: patient or partner	Q9 Q9: Has the dressing or glue come off or been removed?	

For peer review only

	(if so, by patient or professional)						If "Yes", was it taken off by a doctor/nurse/other health specialist?	
	Whether travel is required to change/remove dressing (i.e. seeing nurse or GP/post op visits)	✓	✓					Not relevant to early post-operative period
	Any discomfort during removal	✓	✓	✓	✓		Q10 Was there any discomfort when removing the dressing?	
	Any pain during removal	✓	✓	✓		DE: Causes 'pain', skin is 'tender', 'sore', pulls hairs, sticks to skin)<br>RM: 'Pain on removal of the dressing'	Q11 Was there any pain when removing the dressing?	
**Wound protection**	Dressings protecting the wound				✓		Q12 Has the wound felt protected? (i.e. from catching on anything or being knocked)	
	Whether dressing/wound rubs on clothes	✓	✓	✓		LR: Awkwardness of wearing clothes over dressing		
	Whether dressing/wound catches on other things	✓	✓	✓		CM: bedsheets		
**Impact on daily activities**	Ability to get back to work	✓	✓				Q13: Have you been able to perform everyday tasks? (i.e. showering/ bathing, getting dressed)	
	Ability to shower/bathe	✓	✓	✓	✓	RM: Described as 'Ability to facilitate personal hygiene' in one study<br>RM: Described as 'Appreciation of possibility to shower' in one study. Also 'satisfaction with the possibility to wash oneself'		
	Ease of movement (e.g sitting, walking, stairs)	✓	✓		✓	DE: Standing up, walking<br>RM: Described as 'Ability to facilitate mobility' in the literature. Also 'Does the dressing limit you in movement?'<br>DE and LR: Includes sneezing/coughing		
	Ability to perform everyday tasks (e.g self-care)	✓	✓	✓		DE: Washing/self-care, driving, walking, housework, cooking, exercise		
	Ease of getting dressed	✓						
	Going to the toilet		✓	✓				
	Self-management of wound		✓	✓	✓	RM: 'Ease of managing wound' was a PRO measured in the first 3 weeks after surgery on a 1-10 scale in one study		
	Change to usual clothing		✓	✓				
	Overall recovery				✓	RM: not a PRO (surgeon rating)		
**Ease of movement**	(Dis)comfort when sitting	✓	✓	✓			Q14: Have you been able to move around easily?	
	(Dis)comfort when lying	✓	✓					

For peer review only

	(Dis)comfort whilst sleeping/sleep quality	✓	✓					
	(Dis)comfort whilst moving	✓	✓	✓		CM: related to tightness of dressing		
**Anxiety about the wound**	Feeling of security/safeness (in relation to the wound?)	✓	✓	✓			Q15: Have you felt any anxiety about your wound?	
	Feeling of vulnerability		✓	✓				
	Not having to worry about wound/dressing	✓	✓	✓		DE: 'You can just forget about it, you don't have to think about		
	Feeling protected	✓	✓	✓				
	Stress levels/psychological discomfort/anxiety	✓	✓					
	Feeling constricted by dressing	✓	✓					
	Cleanliness of environment	✓	✓	✓		DE: Described as 'hygiene', 'coming into contact with bugs'		
	Fear of infection	✓	✓	✓				
	Anxiety about bodily contents spilling out/wound bursting open	✓	✓	✓		LR: Described as 'coming open', 'split apart', 'rupture'		
**Satisfaction with dressing/ not having a dressing**	Satisfaction with (appearance of) dressing	✓	✓		✓	LR: 'Neatness', 'prominence'<br>RM: Measured as 'How satisfied overall do you feel with your dressing?'	Q16: Have you felt satisfied with having/not having a dressing?	
	Whether patient would prefer to see the wound	✓	✓			DE: Anxiety/reassurance/fear/discomfort<br>RM: Described as 'how well the incision could be seen under the dressing' and 'transparency'		
	Degree dressing fits contours of the skin/clothing	✓	✓		✓	RM: Described as 'Conformability of the dressing to the wound' in one study		
	Dignity			✓		CM: Having a dressing gives more dignity		
	Confidence			✓		CM: Confidence to walk around without having to worry		
	Appreciation of absence of bandage				✓			
	How long dressing stays on	✓	✓	✓	✓	CM: 'the longer you leave a dressing on the harder it is to get off'		
	Ease of removal	✓	✓	✓	✓	RM: 'Ease of dressing application' was measured in one study but this was rated by a surgeon not patient<br>LR: Any exudate from removal<br>DE, CM,: Any remnants remaining after removal ('bits of glue')		
	Degree of stickiness	✓	✓	✓	✓	CM: stitches stick to legs when sitting (gynae)		

For peer review only

						DE: Mostly considered to be positive, but sometimes makes removal tricky<br>DE: Described as: 'It stays in place and doesn't wriggle up'<br>RM: Described as 'Dressing integrity' in one study also 'How much the dressing had loosened'<br>CM: dressings don't stick well because of body hair		
	Awareness of dressing/wound	✓	✓	✓	✓			
	If patient reapplies, ease of reapplying dressing	✓	✓		✓			
	Whether additional support or materials provided	✓	✓	✓				
	Whether patient uses own dressing	✓	✓	✓				
	If patient reapplies, ease of reapplying dressing	✓	✓		✓			
	Overall satisfaction/satisfaction with overall experience				✓			
**Wound appearance**	Perceptions of healing	✓	✓	✓	✓	DE: Described as 'healing very nicely', 'rate of healing'<br>LR: Sub-codes – 'quality of healing', 'speed of healing', 'whether healed or not', 'suggested as main outcome'. Patients often discuss scab formation when talking about healing.<br>RM: Measured as 'Effectiveness (wound healing)' in one study 1= well healed, 3=poorly healed, not a PRO. Also measured as a PRO in another study 'Has your wound healed?'<br>CM: Healing could be a standalone category (one of the outcomes): definition of healing, healing time		Relevant to longer term outcomes of wound healing (not relevant within first days of surgery)<br>Patients' wounds not visible if dressing applied
	Bruising	✓	✓			DE: Described as 'black and blue'		
	Colour	✓	✓	✓	✓	DE: Described as 'purple'/'pink'/'grey'/'red' (see inflammation')<br>LR: 'Black and bluish' (also fit under 'bruising', as above)<br>RM: Redness<br>CM: Colour was talked about more in the context of healing, ie as an indicator (for the patients) of whether or not the wound was healing		
	Cosmesis/aesthetics		✓		✓	RM: Described as 'cosmesis', 'cosmetic outcome' and 'cosmetic result' in the literature		

For peer review only

	Scars	✓	✓	✓	✓	DE: A long term measure<br>RM: Pigmentation, scar colour, prescence of inflammation, suppleness or pliability, scar height or evenness with the surrounding skin, using modified Vancouver Burn Assessment Scale		
	Size	✓	✓	✓				
	Scabbing	✓	✓	✓		DE: Associated with healing		
	Inflammation/swelling	✓	✓	✓		DE: Described as 'burning'<br>CM: Described by patients as an outcome		
	Overall appearance of wound	✓	✓	✓		DE: Described as 'unsightly', 'neat', 'tidy', 'messy' LR: 'ugly'<br>CM: 'Smooth'		
	Maceration of the skin				✓	RM: not a PRO		
	Satisfaction with the appearance of the wound				✓			
	Whether patient would prefer to see the wound	✓	✓					

For peer review only

Outcome as described by author	Wording used to measure outcome (where reported)	Who reported the outcome	Rating/measurement scale	Assessment time point	Study reference	Study source *
Pain	-	Patient reported	0 to 10 cm VAS	Day 1, Day 10	Amin 2009	2,3
Cosmesis of wound	Cosmesis of wound	Patient reported	Poor 0 5 Excellent 10	3 months post-op	Amin 2009	2,3
Ability to shower same day	Ability to shower same day	Patient reported	Poor (0) Satisfactory (1) Excellent(2)	3 months post-op	Amin 2009	2,3
Need visit GP for wound care	Need visit GP for wound care	Patient reported	"Could have done without; Didn't mind; N/A"	3 months post-op	Amin 2009	2,3
Pain on removing clips	Pain on removing clips	Patient reported	Yes/No	3 months post-op	Amin 2009	2,3
Pain/Tightness of wound after 3 months	Pain/Tightness of wound after 3 months	Patient reported	Slight(0)Moderate(1)Significant(2)	3 months post-op	Amin 2009	2,3
Overall comfort with wound	Overall comfort with wound	Patient reported	Poor(0)Satisfactory(1)Excellent(2)	3 months post-op	Amin 2009	2,3
Allergic reactions	Allergic reactions	Patient reported	Yes(0)No(1)	3 months post-op	Amin 2009	2,3
Overall satisfaction	Overall satisfaction	Patient reported	Poor 0; 5; 10 Excellent	3 months post-op	Amin 2009	2,3
Cosmetic appearance	How would you evaluate your skin stitches after the operation?	Patient reported	1 (very poor) 2 (poor) 3 (medium) 4 (good) 5 (very good)	Day 40 post-op	Asvar 2009	2
Satisfaction	What is your satisfaction?	Patient reported	1 (very poor) 2 (poor) 3 (medium) 4 (good) 5 (very good)	Day 40 post-op	Asvar 2009	2
Perceived patient satisfaction	-	Physician reported	"Ratings"	Day 10	Blondeel 2004	2,3
Cosmesis	-	Patient reported	"Ratings" – unspecified categories but included "outstanding"	Day 10	Blondeel 2004	2,3
Overall comfort	-	Patient reported	"Ratings"	Day 10	Blondeel 2004	2,3

* 1= Cochrane 2011 dressings review; 2= Cochrane 2014 tissue adhesive review; 3=Chow 2010 tissue adhesive review; 4=additional studies provided by authors of the Cochrane dressings review update

For peer review only

Outcome as described by author	Wording used to measure outcome (where reported)	Who reported the outcome	Rating/measurement scale	Assessment time point	Study reference	Study source *
Ability to shower	-	Patient reported	"Ratings"	Day 10	Blondeel 2004	2,3
Dressing changes	-	Patient reported	"Ratings"	Day 10	Blondeel 2004	2,3
Tension at the wound	-	Patient reported	"Ratings"	Day 10	Blondeel 2004	2,3
Hygiene problems	-	Patient reported	"Ratings"	Day 10	Blondeel 2004	2,3
Allergic reaction	-	Patient reported	"Ratings"	Day 10	Blondeel 2004	2,3
Overall satisfaction	-	Patient reported	"Ratings"	Day 10	Blondeel 2004	2,3
Cosmesis	-	Physician reported	validated Modified Hollander Instrument	Day 30	Blondeel 2004	2,3
Dressing changes	-	Observer reported	n/a	unspecified	Burke 2012	4
Incidence of blistering	-	Observer reported	n/a	unspecified	Burke 2012	4
Cosmetic outcome	-	Unspecified	Scale, 1-10	Unclear when specific outcomes were recorded – several follow ups at Day 1, 3, 5-7 and 2 weeks, 1, 3, 6, 12 months	Chibbaro 2009	2,3
Wound management by the patients	-	Unspecified	Unspecified	Unclear when specific outcomes were recorded – several follow ups at Day 1, 3, 5-7 and 2 weeks, 1, 3, 6, 12 months	Chibbaro 2009	2,3
Satisfaction	-	Patient reported	Scale, 1-10	Unclear when specific outcomes were recorded – several follow ups at Day 1, 3,	Chibbaro 2009	2,3

* 1= Cochrane 2011 dressings review; 2= Cochrane 2014 tissue adhesive review; 3=Chow 2010 tissue adhesive review; 4=additional studies provided by authors of the Cochrane dressings review update

For peer review only

Outcome as described by author	Wording used to measure outcome (where reported)	Who reported the outcome	Rating/measurement scale	Assessment time point	Study reference	Study source *
				5-7 and 2 weeks, 1, 3, 6, 12 months		
Appreciation of possibility to shower	-	Unspecified	Unspecified	Unclear when specific outcomes were recorded – several follow ups at Day 1, 3, 5-7 and 2 weeks, 1, 3, 6, 12 months	Chibbaro 2009	2,3
Appreciation of absence of head bandage	-	Unspecified	Unspecified	Unclear when specific outcomes were recorded – several follow ups at Day 1, 3, 5-7 and 2 weeks, 1, 3, 6, 12 months	Chibbaro 2009	2,3
Number of dressing changes	-	Collected by surgeons, nurses and junior medical staff	n/a	during hospital stay	Cosker 2005	1
Blistering	-	Collected by surgeons, nurses and junior medical staff	n/a	during hospital stay	Cosker 2005	1
When the dressing required changing	-	Collected by surgeons, nurses and junior medical staff	n/a	during hospital stay	Cosker 2005	1
Reason for dressing change	-	Collected by surgeons, nurses and junior medical staff	n/a	during hospital stay	Cosker 2005	1
Number of post operative days	-	Collected by surgeons, nurses and junior medical staff	n/a	during hospital stay	Cosker 2005	1

* 1= Cochrane 2011 dressings review; 2= Cochrane 2014 tissue adhesive review; 3=Chow 2010 tissue adhesive review; 4=additional studies provided by authors of the Cochrane dressings review update

For peer review only - http://bmjopen.bmj.com/site/about/guidelines.xhtml

Outcome as described by author	Wording used to measure outcome (where reported)	Who reported the outcome	Rating/measurement scale	Assessment time point	Study reference	Study source *
Volume of wound exudate	-	Collected by surgeons, nurses and junior medical staff	Amount of seepage through the dressing and the apparent volume found on the wound after the dressing was removed	during hospital stay	Cosker 2005	1
Satisfaction with wound closure method	-	Patient reported	Satisfied / Dissatisfied	24 to 48 hrs, 4 to 6 weeks, 3 months post-op	Dowson 2006	2,3
Satisfaction with appearance of the wound	-	Patient reported	Satisfied / Dissatisfied	24 to 48 hrs, 4 to 6 weeks, 3 months post-op	Dowson 2006	2,3
Degree of pain	-	Patient reported	Scale, 1-10	First 3 weeks after surgery	Gennari 2004	3
Ease of managing wound	-	Patient reported	Scale, 1-10	First 3 weeks after surgery	Gennari 2004	3
Ability to take a shower	-	Patient reported	Scale, 1-10	First 3 weeks after surgery	Gennari 2004	3
Postoperative visits	-	Patient reported	Scale, 1-10	First 3 weeks after surgery	Gennari 2004	3
Use of dressings	-	Patient reported	Scale, 1-10	First 3 weeks after surgery	Gennari 2004	3
Comfort	-	Interview with physician	Unspecified	1, 2, & 4 weeks post-op	Greene 1999	2,3
Presence of a pulling sensation	-	Interview with physician	Unspecified	1, 2, & 4 weeks post-op	Greene 1999	2,3
Appreciation of the lack of suture removal	-	Interview with physician	Unspecified	1, 2, & 4 weeks post-op	Greene 1999	2,3
Discomfort in connection with removal of dressing	-	Unspecified	Unspecified	during hospital stay	Holm 1998	1
Number of dressing changes	-	Observer reported	n/a	during hospital stay	Holm 1998	1

* 1= Cochrane 2011 dressings review; 2= Cochrane 2014 tissue adhesive review; 3=Chow 2010 tissue adhesive review; 4=additional studies provided by authors of the Cochrane dressings review update

For peer review only

For peer review only - http://bmjopen.bmj.com/site/about/guidelines.xhtml

Outcome as described by author	Wording used to measure outcome (where reported)	Who reported the outcome	Rating/measurement scale	Assessment time point	Study reference	Study source *
Adhesion of dressing to the skin	-	Observer reported	Unspecified	daily inspection until discharge	Holm 1998	1
Cosmetic result	-	Observer reported	1 to 5 (higher=better)	3 months post-op	Holm 1998	1
Width of the scar	-	Observer reported	1 to 5 (higher=better)	3 months post-op	Holm 1998	1
Downbinding of the scar	-	Observer reported	1 to 5 (higher=better)	3 months post-op	Holm 1998	1
Colour of the scar	-	Observer reported	1 to 5 (higher=better)	3 months post-op	Holm 1998	1
Elevation of the scar	-	Observer reported	1 to 5 (higher=better)	3 months post-op	Holm 1998	1
Cosmetic outcome	-	Observer reported	1 to 5 (higher=better)	3 months post-op	Holm 1998	1
Supposed inconvenience of the scar	-	Observer reported	1 to 5 (higher=better)	3 months post-op	Holm 1998	1
Exudate	-	Observer reported	Unspecified	daily inspection until discharge	Holm 1998	1
Leakage	-	Observer reported	Unspecified	daily inspection until discharge	Holm 1998	1
Transparency	-	Observer reported	Unspecified, but included milky and slightly milky	daily inspection until discharge	Holm 1998	1
Dressing changes	-	Observer reported	No. of days stayed in place	unspecified	Holm 1998	1
Reasons for dressing changes	-	Observer reported	Unspecified	unspecified	Holm 1998	1
Maceration of the skin	-	Observer reported	Unspecified	unspecified	Holm 1998	1
Post operative wound infection	-	Observer reported	Unspecified	unspecified	Holm 1998	1
Comfort	-	Observer reported	Yes/No	1 week and 1 month	Keng 1989	2
Cosmesis	-	Observer reported	1 (poor) to 5 (excellent)	1 week and 1 month	Keng 1989	2

* 1= Cochrane 2011 dressings review; 2= Cochrane 2014 tissue adhesive review; 3=Chow 2010 tissue adhesive review; 4=additional studies provided by authors of the Cochrane dressings review update

For peer review only

For peer review only - http://bmjopen.bmj.com/site/about/guidelines.xhtml

Outcome as described by author	Wording used to measure outcome (where reported)	Who reported the outcome	Rating/measurement scale	Assessment time point	Study reference	Study source *
Satisfaction with the incision closure	-	Patient verbal report; response recorded by staff	Either "satisfied" or "dissatisfied"	3 month follow up	Kent 2014	2
Overall appearance	-	Patient verbal report; response recorded by staff	Either "satisfied" or "dissatisfied"	3 month follow up	Kent 2014	2
Satisfaction with the techniques of skin closure	-	Patient reported	VAS between 0 and 100, where 100 represented maximal satisfaction.	Between 8 and 12 weeks post-op	Khan 2006	2,3
Dressing or superficial wound discomfort	-	Unspecified	Linear analogue scale	5 days post -op	Law 1987	1
Dressing preference	-	Unspecified	Unspecified	unspecified	Law 1987	1
Wound infection	-	Unspecified	Discharge of purulent material	unspecified	Law 1987	1
Number of dressing changes	-	Unspecified	n/a	unspecified	Law 1987	1
Quality of the final scar	-	Unspecified	Unspecified	unspecified	Law 1987	1
Blisters	-	Observer reported	Defined as any lifting of the epidermis with underlying fluid	48 hours post-op and 5 days	Lawrentschuk 2002	1
Wound condition	-	Observer reported	Unspecified	5 days	Lawrentschuk 2002	1
Swelling	-	Observer reported	Measured thigh girth	48 hours post-op and 5 days	Lawrentschuk 2002	1
Wound infection	-	Observer reported	Unspecified	unspecified	Lawrentschuk 2002	1
Cosmetic appearance	-	Patient reported	100mm VAS (0 worst outcome, 100 best outcome)	3 months	Livesey 2009	2,3
Satisfaction with the scar	-	Patient reported	100mm VAS (0 extreme dissatisfaction, 100 complete satisfaction)	3 months	Livesey 2009	2,3

* 1= Cochrane 2011 dressings review; 2= Cochrane 2014 tissue adhesive review; 3=Chow 2010 tissue adhesive review; 4=additional studies provided by authors of the Cochrane dressings review update

For peer review only

Outcome as described by author	Wording used to measure outcome (where reported)	Who reported the outcome	Rating/measurement scale	Assessment time point	Study reference	Study source *
Appearance of the wound	-	Patient reported	5 point Likert scale (1=much better than expected, 2= better than expected, 3= as expected, 4= worse than expected, 5=much worse than expected)	3 months	Livesey 2009	2,3
Discomfort (pain in the past 48 hrs)	-	Patient reported	0 to 10 cm VAS	2-3 & 7-10 days post-op	Michie 1994	1
Discomfort (pain on removal of the dressing	-	Patient reported	0 to 10 cm VAS	2-3 & 7-10 days post-op	Michie 1994	1
Overall comfort	-	Patient reported	0 to 10 cm VAS	2-3 & 7-10 days post-op	Michie 1994	1
Wound itching (in the past 48hrs)	-	Patient reported	0 to 10 cm VAS	2-3, 7-10 days, 4 weeks, 7 months post-op	Michie 1994	1
Wound pulling (in the past 48 hrs)	-	Patient reported	0 to 10 cm VAS	2-3, 7-10 days, 4 weeks, 7 months post-op	Michie 1994	1
Conformability of the dressing to the wound	-	Surgeon reported	4 point rating scale (excellent, good, fair, poor)	7-10 days post-op	Michie 1994	1
Ability to contain exudate	-	Surgeon reported	4 point rating scale (excellent, good, fair, poor)	7-10 days post-op	Michie 1994	1
Ability to protect the wound	-	Surgeon reported	4 point rating scale (excellent, good, fair, poor)	7-10 days post-op	Michie 1994	1
Ability to facilitate mobility	-	Surgeon reported	4 point rating scale (excellent, good, fair, poor)	7-10 days post-op	Michie 1994	1
Ability to facilitate personal hygiene	-	Surgeon reported	4 point rating scale (excellent, good, fair, poor)	7-10 days post-op	Michie 1994	1
Overall impression of the incision	-	Patient reported	0 to 10 cm VAS	2-3 & 7-10 days post-op	Michie 1994	1
Evaluation of resulting scar	Pigmentation, scar colour, prescence of inflammation, suppleness/pliability,	Patient and surgeon reported	modified Vancouver Burn Assessment Scale (0 to 3 score)	4 weeks and 7 months post-op	Michie 1994	1

* 1= Cochrane 2011 dressings review; 2= Cochrane 2014 tissue adhesive review; 3=Chow 2010 tissue adhesive review; 4=additional studies provided by authors of the Cochrane dressings review update

For peer review only

Outcome as described by author	Wording used to measure outcome (where reported)	Who reported the outcome	Rating/measurement scale	Assessment time point	Study reference	Study source *
	scar height/evenness with the surrounding skin					
Ease of dressing application	-	Surgeon reported	Yes/No	2-3 & 7-10 days post-op	Michie 1994	1
Ease of dressing removal	-	Surgeon reported	Yes/somewhat difficult	2-3 & 7-10 days post-op	Michie 1994	1
Cosmetic result	-	Surgeon reported	4 point rating scale (excellent, good, fair, poor)	7-10 days post-op	Michie 1994	1
Infection	-	Surgeon reported	Unspecified	2-3 & 7-10 days post-op	Michie 1994	1
Pain upon palpation of the wound	-	Surgeon reported	3 point scale including Somewhat/no	7-10 days post-op	Michie 1994	1
Overall wound aspect	-	Surgeon reported	3 point scale including Excellent/good	7-10 days post-op	Michie 1994	1
Overall recovery	-	Surgeon reported	3 point scale including Excellent	7-10 days post-op	Michie 1994	1
Presence of small stitch abscess	-	Surgeon reported	Yes/no	7-10 days post-op	Michie 1994	1
Satisfaction with wound cosmesis	-	Parent reported	100mm VAS	2 to 3 weeks and 3 months follow up assessment (although unable to complete latter assessment as only 9 patients returned)	Ong 2002	2,3
Level of satisfaction with early postoperative management of the wound (regarding requirement of a return visit for medications, the possibility to wash oneself, the stuture removal)	-	Patient reported (verbal)	Numerical scale 0 to 10 (0-4, poor; 5-6, mild;7-8, good; 9-10, excellent)	15 days, 1, 3, 6 and 12 months	Pronio 2011	2

* 1= Cochrane 2011 dressings review; 2= Cochrane 2014 tissue adhesive review; 3=Chow 2010 tissue adhesive review; 4=additional studies provided by authors of the Cochrane dressings review update

For peer review only

Outcome as described by author	Wording used to measure outcome (where reported)	Who reported the outcome	Rating/measurement scale	Assessment time point	Study reference	Study source *
Discomfort	-	Unspecified	Unspecified	15 days, 1, 3, 6 and 12 months	Pronio 2011	2
Pain	-	Unspecified	Unspecified	15 days, 1, 3, 6 and 12 months	Pronio 2011	2
Pain	Pain resulting from dressing usage and not including mobilisation	Patient reported	Yes/no, 10 point VAS (0=no problems, 10=unbearable problems)	At dressing removal (mean 6 and 7 days)	Ravnskog 2001	4
Itching	-	Patient reported	Yes/no, 10 point VAS (0=no problems, 10=unbearable problems)	At dressing removal (mean 6 and 7 days)	Ravnskog 2001	4
Burning	Burning pain refering solely to a dressing-related sensation felt under the dressing	Patient reported	Yes/no, 10 point VAS (0=no problems, 10=unbearable problems)	At dressing removal (mean 6 and 7 days)	Ravnskog 2001	4
Discomfort during use of dressing	-	Patient reported	Yes/no, 10 point VAS (0=no problems, 10=unbearable problems)	At dressing removal (mean 6 and 7 days)	Ravnskog 2001	4
Pain at dressing removal	-	Patient reported	Yes/no, 10 point VAS (0=no problems, 10=unbearable problems)	At dressing removal (mean 6 and 7 days)	Ravnskog 2001	4
Skin damage (erythema, blisters or skin injury)		Observer reported	Small; 1-2cm, medium; 2-5cm, large<5cm	during hospital stay	Ravnskog 2001	4
Satisfaction with the cosmetic result	-	Patient reported	Dichotomous (satisfaied/dissatisfied)	Day 10 and day 90	Romero 2011	2
Pain at port sites	-	Surgeon reported	Unspecified	Day 10 and day 90	Romero 2011	2
Satisfaction with cosmetic result	-	Patients were asked by the senior dermatologist	1 (very satisfied) to 5 (not satisfied)	1 year post-op	Shamiyeh 2001	2,3
Degree of pain	-	Patient reported	Scale, 1-10	3 months post-op	Sniezek 2007	2,3
Ease of managing the surgical wound	-	Patient reported	Scale, 1-10	3 months post-op	Sniezek 2007	2,3

* 1= Cochrane 2011 dressings review; 2= Cochrane 2014 tissue adhesive review; 3=Chow 2010 tissue adhesive review; 4=additional studies provided by authors of the Cochrane dressings review update

For peer review only - http://bmjopen.bmj.com/site/about/guidelines.xhtml

For peer review only

Outcome as described by author	Wording used to measure outcome (where reported)	Who reported the outcome	Rating/measurement scale	Assessment time point	Study reference	Study source *
Ability to take a shower	-	Patient reported	Scale, 1-10	3 months post-op	Sniezek 2007	2,3
Overall satisfaction	-	Patient reported	Scale, 1-10	3 months post-op	Sniezek 2007	2,3
Cosmetic appearance	-	Patient reported	Scale, 1-10	3 months post-op	Sniezek 2007	2,3
Comfort of the dressing (discomfort at mobilization)	-	Patient reported	3 point scale (no discomfort at all, minor problems, severe discomfort)	Daily for 4 days after surgery	Vogt 2007	1
Comfort of the dressing (pain at dressing changes)	-	Patient reported	3 point scale (no discomfort at all, minor problems, severe discomfort)	Daily for 4 days after surgery	Vogt 2007	1
Comfort of the dressing (skin problems)	-	Patient reported	3 point scale (no discomfort at all, minor problems, severe discomfort)	Daily for 4 days after surgery	Vogt 2007	1
Signs of infection - redness, tenderness, swelling, exudates	-	Observer reported	Unspecified	2 weeks post-op	Vogt 2007	1
Wound complications - haematoma or persistent lymph oozing, surgical revision	-	Observer reported	Unspecified	2 weeks post-op	Vogt 2007	1
Length of hospital stay	-	Observer reported	n/a	n/a	Vogt 2007	1
Number of dressing changes	-	Observer reported	n/a	during postoperative stay	Vogt 2007	1
Patient comfort (difficulty in removing the dressings)	-	Nurse reported	Unspecified	Day 5	Wikblad 1995	1
Patient comfort (pain at dressing removal)	-	Nurse reported	3 point scale from "no pain at all" to "very painful"	Day 5	Wikblad 1995	1
Number of bandage changes	-	Nurse reported	n/a	Day 1 to day 5	Wikblad 1995	1
Reason for bandage changes	-	Nurse reported	n/a	Day 1 to day 5	Wikblad 1995	1

* 1= Cochrane 2011 dressings review; 2= Cochrane 2014 tissue adhesive review; 3=Chow 2010 tissue adhesive review; 4=additional studies provided by authors of the Cochrane dressings review update

For peer review only

For peer review only - http://bmjopen.bmj.com/site/about/guidelines.xhtml

Outcome as described by author	Wording used to measure outcome (where reported)	Who reported the outcome	Rating/measurement scale	Assessment time point	Study reference	Study source *
Effectiveness (wound healing)	-	Independent raters judging photograph	1=well healed (wound edges well together; a gap of <5% length of the incision allowed with no or slight redness), 2=partially healed (gaps >5% but <20% with slight to excessive redness), 3=poorly healed (gaps>20% with excessive redness)	Day 5 and 4 weeks after surgery	Wikblad 1995	1
Redness	-	Independent raters judging photograph	0=no redness, 1=slight redness, 2= excessive redness	Day 5 and 4 weeks after surgery	Wikblad 1995	1
Wound healing	Do you think the wound is well/partially/poorly healed?	Patient reported	3 point scale	Once a week after discharge for 3 weeks	Wikblad 1995	1
Skin changes	-	Patient reported	Unspecified	Once a week after discharge for 3 weeks	Wikblad 1995	1
Redness	Is the wound red?	Patient reported	Yes/No	Once a week after discharge for 3 weeks	Wikblad 1995	1
Swelling	Does the wound look swollen?	Patient reported	Yes/No	Once a week after discharge for 3 weeks	Wikblad 1995	1
Itching	Does the wound itch?	Patient reported	Yes/No	Once a week after discharge for 3 weeks	Wikblad 1995	1
Skin changes (erythema and blisters)	-	Independent raters judging photograph	n/a	Day 5	Wikblad 1995	1
Clinical utility (ability to allow ongoing evaluation of the incision)	How well the incision could be seen through the dressing	Nurse reported	1=good, 2=partially, 3=not atall	Day 1 to day 5	Wikblad 1995	1
How much the dressing had loosened	-	Nurse reported	graded scale from 1 to 3	Day 1 to day 5	Wikblad 1995	1
Treatment with antibiotics	-	Nurse reported	yes/no	4 weeks after surgery	Wikblad 1995	1

* 1= Cochrane 2011 dressings review; 2= Cochrane 2014 tissue adhesive review; 3=Chow 2010 tissue adhesive review; 4=additional studies provided by authors of the Cochrane dressings review update

For peer review only

Outcome as described by author	Wording used to measure outcome (where reported)	Who reported the outcome	Rating/measurement scale	Assessment time point	Study reference	Study source *
Safety (presence of infection - wound culture)	-	Clinical sample	n/a - lab sample	Day 5	Wikblad 1995	1
Dressing awareness	How aware are you of your dressing most of the time?	Patient reported	10 cm visual analogue scale with three anchors at 0,5 and 10cm	Day 1 to day 5	Wynne 2004	1
Movement limitation	Does the dressing limit you in moving about?	Patient reported	10 cm visual analogue scale with three anchors at 0,5 and 10cm	Day 1 to day 5	Wynne 2004	1
Comfort with removal	How comfortable do you feel during dressing changes?	Patient reported	10 cm visual analogue scale with three anchors at 0,5 and 10cm	Day 1 to day 5	Wynne 2004	1
Overall satisfaction	How satisfied overall do you feel with your dressing?	Patient reported	10 cm visual analogue scale with three anchors at 0,5 and 10cm	Day 1 to day 5	Wynne 2004	1
Wound healing - approximation	-	Observer reported	4 categories (total, partial;<2cm of superficial separation, moderate;>2cm of superficial separation, dehisced; complete separation of layers)	Day 1 to day 5	Wynne 2004	1
Wound healing - skin itegrity	-	Observer reported	3 categories (normal; pink no redness, inflammed; heat redness swelling, macerated within a 2.5cm border of the incision)	Day 1 to day 5	Wynne 2004	1
Wound infection	-	Observer reported	CDC criteria	unspecified	Wynne 2004	1
Dressing integrity	-	Observer reported	3 catergories - suture line exposed, poorly sealed, well sealed	unspecified	Wynne 2004	1
Experience with wound	Has your chest wound healed?	Patient reported	Yes/No	One month post-discharge	Wynne 2004	1
Antibiotic therapy	Has your doctor given you any antibiotics for	Patient reported	Yes/No	One month post-discharge	Wynne 2004	1

* 1= Cochrane 2011 dressings review; 2= Cochrane 2014 tissue adhesive review; 3=Chow 2010 tissue adhesive review; 4=additional studies provided by authors of the Cochrane dressings review update

For peer review only

For peer review only - http://bmjopen.bmj.com/site/about/guidelines.xhtml

Outcome as described by author	Wording used to measure outcome (where reported)	Who reported the outcome	Rating/measurement scale	Assessment time point	Study reference	Study source *
	your chest wound, since you left hospital?					
Experience with wound	Over the past month, has there been any fluid/discharge oozing from the chest wound? If so, how would you describe the fluid: watery, straw coloured; blood stained; pus (think yellow)	Patient reported	Yes/No	One month post-discharge	Wynne 2004	1
Experience with wound	Was a dressing required on your wound?	Patient reported	Yes/No	One month post-discharge	Wynne 2004	1
Experience with wound	Have you had any of the following problems with your chest wound? Redness, swelling, pain, tenderness	Patient reported	Yes/No	One month post-discharge	Wynne 2004	1
Experience with wound	Has your local doctor told you at any time your chest wound was infected?	Patient reported	Yes/No	One month post-discharge	Wynne 2004	1

* 1= Cochrane 2011 dressings review; 2= Cochrane 2014 tissue adhesive review; 3=Chow 2010 tissue adhesive review; 4=additional studies provided by authors of the Cochrane dressings review update

For peer review only

Note: Permission to use these measures must be obtained from the author (daisy.elliott@bristol.ac.uk). Work is being conducted to test the reliability, validity and sensitivity of these measures.

Bluebelle study

**Bluebelle dressing allocation:**	***Simple dressing***
**Study ID:**	
**Participant name:**	
**Date of surgery:**	
**Date completed:**	

Wound Experience Questionnaire

We are interested in how your wound(s) have healed since your operation and your experience of having a dressing, as part of the Bluebelle study. Please complete this short questionnaire yourself. You can complete the questionnaire as soon as you feel ready, but ideally this will be within four days of having your operation. If there is more than one wound, please respond **thinking about just one wound** – either the main one or another wound if there have been any concerns about how it has been healing.

When you have completed the questionnaire, please return it in the pre-paid envelope provided.

Section 1: Wound comfort

		Not at all	A little	Quite a bit	A lot
1.	Has your wound been itchy?	☐	☐	☐	☐
2.	Has your wound been painful?	☐	☐	☐	☐
3.	Has your wound had a pulling sensation?	☐	☐	☐	☐
4.	Has your wound felt tight?	☐	☐	☐	☐
5.	Has your wound been smelly?	☐	☐	☐	☐

Section 2: Removing the dressing

		Yes	No	
6.	Has the original dressing been removed/come off on its own?	☐	☐ →	*If "No" go to Section 3, Question 7*

If "Yes", how did it come off?

		Yes
a)	A doctor/nurse/other health professional	☐
b)	You/your partner/friend/family member	☐
c)	It came off on its own	☐

		Not at all	A little	Quite a bit	A lot
d)	Did you feel any pain when the dressing was removed?	☐	☐	☐	☐
e)	Did you feel any anxiety when the dressing was removed?	☐	☐	☐	☐

Section 3: Experience of having a dressing

		Not at all	A little	Quite a bit	A lot
7.	Has your dressing prevented you from showering or washing?	☐	☐	☐	☐
8.	Has your wound felt protected?	☐	☐	☐	☐
9.	Have you felt any anxiety about your wound in relation to your dressing(s)?	☐	☐	☐	☐
10.	Are you satisfied with your dressing(s)?	☐	☐	☐	☐

Additional comments:

....

....

....

Thank you for completing this questionnaire.

For peer review only

Note: Permission to use these measures must be obtained from the author (daisy.elliott@bristol.ac.uk). Work is being conducted to test the reliability, validity and sensitivity of these measures.

Bluebelle study

**Bluebelle dressing allocation:**	***Simple dressing***
**Study ID:**	
**Participant name:**	
**Date of surgery:**	
**Date completed:**	
**Completed by *(please tick)*:**	☐ Healthcare professional<br>☐ Participant<br>☐ Other (state):

Wound Management Questionnaire

To be completed by a healthcare professional up to 4 days after surgery

Or

To be completed by the participant up to 4 days after surgery if the participant is discharged before completion by a healthcare professional

If there is more than one wound, please respond **thinking about just one wound** – either the main one or another wound if there have been any concerns about how it has been healing. When you have completed the questionnaire, please return it in the pre-paid envelope provided.

Section 1: Wound leakage

In the past 24 hours…

	**Not at all**	**A little**	**Quite a bit**	**A lot**
1. Has fluid from the wound leaked through the dressing?	☐	☐	☐	☐

If "Not at all", go to Section 2, Question 3

	**Yes**	**No**	**If "Yes", how many times?**
2. Has the leakage required bedding or clothes to be changed?	☐	☐	______________

Section 2: Dressings

In the past 24 hours…

	**Yes**	**No**	
3. Has the original dressing been replaced?	☐	☐	If "No", questionnaire is complete

If "Yes", how many times? ___________

4. Why was the dressing replaced? **Yes** (tick all that apply)

	Yes (tick all that apply)	
a) Routine change	☐	
b) The dressing was saturated	☐	
c) The wound was irritated	☐	
d) The wound was blistered	☐	
e) Another reason	☐	If "Yes", please specify what the reason was ____

Additional comments:

………

………

………

Thank you for completing this questionnaire.

For peer review only

Table 1: Consolidated criteria for reporting qualitative research (COREQ)

	No	Item	Guide questions/description	Comment
**Domain 1: Research team and reflexivity**				
**Personal characteristics**	1	Interviewer/facilitator	Which author/s conducted the interview or focus group?	LR, DE, CMM and CM *(see pages 6 and 7)*
	2	Credentials	What were the researcher's credentials? e.*g. PhD, MD*	DE - BSc, PhD<br>LR - BSc, PhD<br>CMM - BA Hons, PgDip, MA PhD<br>CM – MB ChB BSc
	3	Occupation	What was their occupation at the time of the study?	DE - Qualitative Research Associate in Health Services Research<br>LR - Lecturer in Qualitative Health Science<br>CMM - Qualitative Research Fellow<br>CM - Research Fellow
	4	Gender	Was the researcher male or female?	Females
	5	Experience and training	What experience or training did the researcher have?	DE, LR and CMM have several years of experience conducting qualitative research. This has included completing many qualitative projects and attending training courses and workshops.
**Relationship with participants**	6	Relationship established	Was a relationship established prior to study commencement?	No
	7	Participant knowledge of the interviewer	What did the participants know about the researcher? *e.g. personal goals, reasons for doing the research*	The researchers introduced themselves, explained the purpose of the research and provided an information leaflet about the study
	8	Interviewer characteristics	What characteristics were reported about the interviewer/facilitator? *e.g. Bias, assumptions, reasons and interests in the research topic*	The researchers explained how qualitative research related to main Bluebelle trial
**Domain 2: study design**				
**Theoretical framework**	9	Methodological orientation<br><br>and theory	What methodological orientation was stated to underpin the study? *e.g. grounded theory, discourse analysis, ethnography, phenomenology, content analysis*	Data were analysed thematically using techniques of constant comparison derived from grounded theory methodology *(see page 6)*

For peer review only

For peer review only - http://bmjopen.bmj.com/site/about/guidelines.xhtml

**Participant selection**	10	Sampling	How were participants selected? *e.g. purposive, convenience, consecutive, snowball*	Purposeful *(see pages 6 and 7)*
	11	Method of approach	How were participants approached? e.*g. face-to-face, telephone, mail, email*	Patients were approached face to face by healthcare professionals. Healthcare professionals were contacted by the researchers via email. *(See pages 6 and 7)*
	12	Sample size	How many participants were in the study?	Sixty-four patients and 15 health care professionals from abdominal general surgical specialities and obstetrics *(See Table 1 on pages 7/8)*
	13	Non-participation	How many people refused to participate or dropped out? Reasons?	Two patients were unable to take part due to poor health.
**Setting**	14	Setting of data collection	Where was the data collected? *e.g. home, clinic, workplace*	Patient interviews were conducted whilst patients were in hospital. Health professionals chose a location that was convenient for them (their workplace or a nearby café) or opted to do the interview over the telephone. *(See pages 6 and 7)*
	15	Presence of non-participants	Was anyone else present besides the participants and researchers?	The partners of patients sometimes sat with the patients but spoke very little; their comments were not included in the final analysis.
	16	Description of sample	What are the important characteristics of the sample? *e.g. demographic data, date*	Participants' full details are provided in Table 1, and key information is provided in the results section *(See pages 7/8)*
**Data collection**	17	Interview guide	Were questions, prompts, guides provided by the authors? Was it pilot tested?	A topic guide was developed (based on literature and views of health care professionals in the Bluebelle study team) to ensure that discussions covered the same core issues but with sufficient flexibility to allow new issues of importance to the informants to emerge. Although not piloted, it was adapted as analysis progressed to enable exploration of emerging themes. *(Topic guides are included in Additional File)*
	18	Repeat interviews	Were repeat interviews carried out? *If yes, how many?*	No repeat interviews were carried out
	19	Audio/visual recording	Did the research use audio or visual recording to collect the data?	Interviews were audio-recorded *(see page 6)*
	20	Field notes	Were field notes made during and/or after the	The researchers kept notes throughout data

For peer review only - http://bmjopen.bmj.com/site/about/guidelines.xhtml

			interview or focus group?	collection and analysis *(See page 8)*
	21	Duration	What was the duration of the interviews or focus group?	Interviews lasted an average of 25 minutes (range = 15–50 minutes). *(See page 8)*
	22	Data saturation	Was data saturation discussed?	Data collection continued until the team were confident that saturation had been reached *(See page 7)*
	23	Transcripts returned	Were transcripts returned to participants for comment and/or correction?	Transcripts were not returned to participants for comments or corrections
**Domain 3: analysis and findings**				
**Data analysis**	24	Number of data coders	How many data coders coded the data?	All data were coded by DE or CMM. A subset of approximately half of the interviews (n = 19) was double coded by a third researcher (LR). *(See page 7)*
	25	Description of the coding tree	Did authors provide a description of the coding tree?	A list of issues from the analysis of the interviews and literature search was collated into an item tracking matrix. *(See additional File)*
	26	Derivation of themes	Were themes identified in advance or derived from the data?	Issues which were conceptually similar were organised into categories. For instance, issues such as 'itchiness/irritation', 'presence of pulling sensation', and 'tightness of wound' were mapped into a 'wound comfort' category. *(See Table 2)*
	27	Software	What software, if applicable, was used to manage the data?	NVivo (version 10) was used to analyse the data (See page 6)
	28	Participant checking	Did participants provide feedback on the findings?	Full results were not sent out to all participants to gain respondent validation.
**Reporting**	29	Quotations presented	Were participant quotations presented to illustrate the themes / findings? *Was each quotation identified? e.g. participant number*	The interpretation of each category is supported by illustrative quotes *(See Table 2)*
	30	Data and findings consistent	Was there consistency between the data presented and the findings?	There is consistency between the data presented and the measures developed. The item tracking matrix provides an overview of the key findings and how these were used to develop the initial measure *(See additional file)*
	31	Clarity of major themes	Were major themes clearly presented in the findings?	The themes are clearly presented in the

For peer review only - http://bmjopen.bmj.com/site/about/guidelines.xhtml

				findings *(See pages 8-11)*
	32	Clarity of minor themes	Is there a description of diverse cases or discussion of minor themes?	Yes. Differences between the findings of the interviews and the literature search are discussed, as are the differences in satisfaction between the dressing types *(See pages 9 and 10)*

For peer review only

For peer review only - http://bmjopen.bmj.com/site/about/guidelines.xhtml
